# Supplementary material for: Bacterially derived synthetic mimetics of mammalian oligomannose prime antibody responses that neutralize HIV infectivity
Source: Nat Commun. 2017 Nov 17;8:1601. doi: 10.1038/s41467-017-01640-y (PMC5693931; doi:10.1038/s41467-017-01640-y)
Supplement: Supplementary file 1 — Supplementary Information [file 41467_2017_1640_MOESM1_ESM.pdf]

SUPPLEMENTARY FIGURES

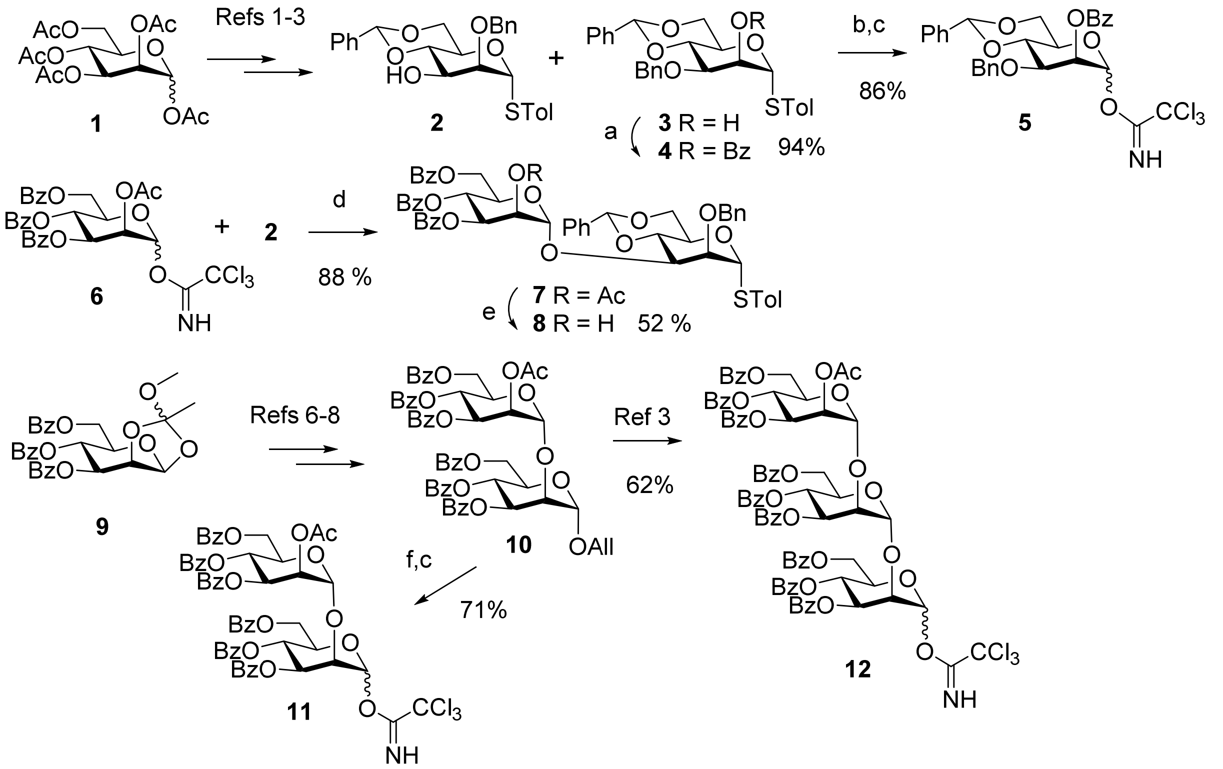

Reagents and conditions: a) BzCl, pyridine; b) NIS, TFA, then piperidine, DCM, 0 °C to RT; c) CCl_3CN, K_2CO_3, DCM; d) TMSOTf, AW MS 4 Å, DCM; e) $H_2NNH_2 \times H_2O$, MeCN; f) $PdCl_2$, MeOH

Supplementary Figure 1 | Synthesis of mannosyl glycosyl donors 5, 11, 12 and acceptor derivative 8. D-Mannose was converted into the thioglycoside acceptor derivatives **2** and **3** according to published procedures[1-3]. The latter product was further transformed into the orthogonally monosaccharide glycosyl donor **5**. Glycosylation of **2** with known donor **6**[3-6] gave the α-(1→3)-linked disaccharide **7**, which produced the disaccharide acceptor **8** upon selective deacetylation. In addition, the α-(1→2)-linked di- and trisaccharide trichloroacetimidate donors **11** and **12** were elaborated from orthoester **9** in a straightforward approach based on published methods[6-8].

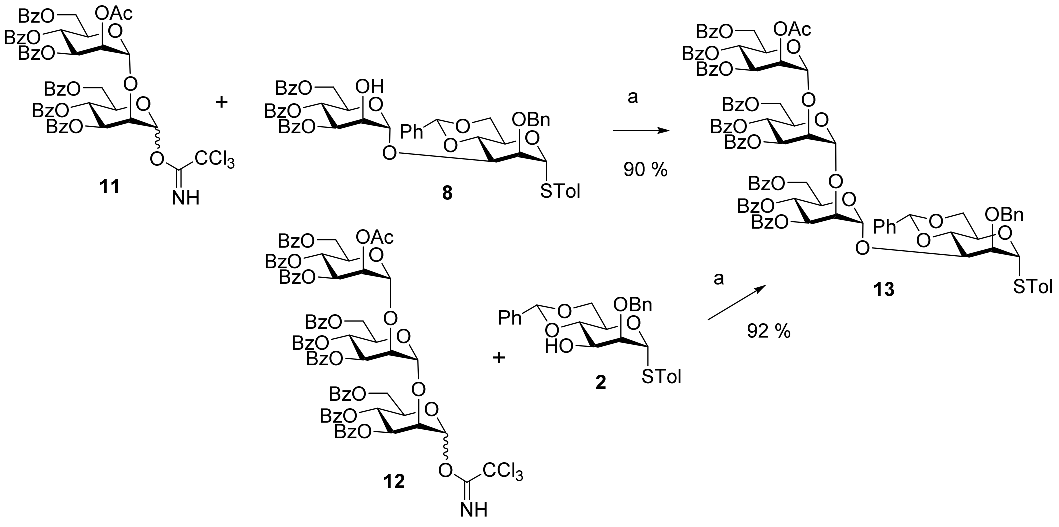

Reagents and conditions: a) TMSOTf, AW MS 4 Å, DCM

Supplementary Figure 2 | Synthesis of tetramannoside 13. Next, the central mannotetraoside **13** was prepared via two routes: A 2+2 coupling of donor **11** and acceptor **8** furnished the thioglycoside **13** in 90 % yield. As an alternative, a 3+1 approach was developed using trisaccharide donor **12** and monosaccharide acceptor **2** (**Supplementary Fig. 1**) to give **13** in excellent yield of 92%.

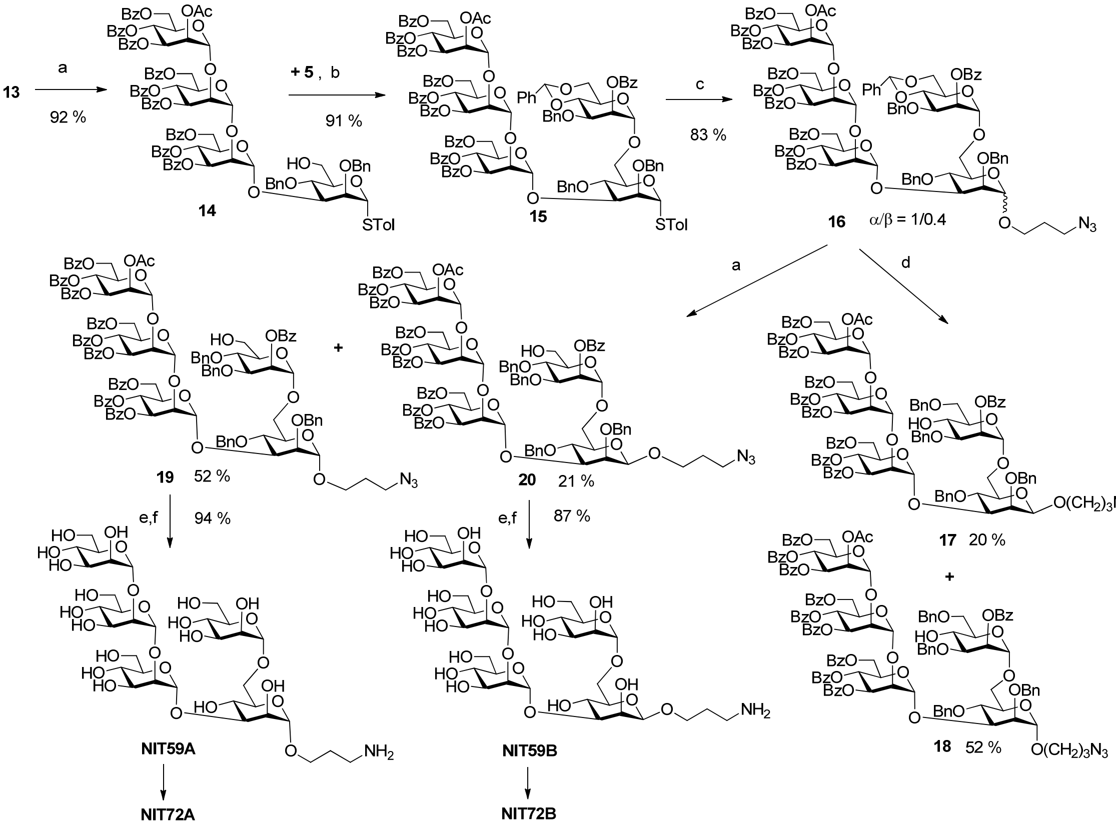

Reagents and conditions: a) Et_3SiH, $BPhCl_2$, AW MS 4 Å; DCM; b) TMSOTf, AW MS 4 Å, DCM; c) 3-azido-1-propanol, NIS, TfOH, AW MS 4 Å, DCM; d) Et_3SiH, TfOH, AW MS 4 Å, DCM; e) NaOMe, MeOH; f) Pd-C, H_2, MeOH/H_2O/AcOH.

Supplementary Figure 3 | Synthesis of mannopentaosides. Regioselective reductive benzylidene opening of the central intermediate **13** afforded the alcohol **14** which was elongated using the 4,6-*O*-benzylidene protected trichloroacetimidate donor **5** (**Supplementary Fig. 1**) to give pentasaccharide **15** in 91% yield. At this stage, the 3-azidopropyl spacer was introduced, which gave an inseparable mixture of anomeric product **16** in low anomeric selectivity, which nonetheless proved beneficial to evaluate the contribution of the anomeric stereochemistry on antibody binding. Separation of the isomers was achieved after reductive opening of the benzylidene group to furnish the 4-OH derivatives **17** and **18** as well as the 6-OH derivatives **19** and **20**, respectively, ready for further chain elongation as well as for global deprotection. In this way, the target pentasaccharides **21** and **22** were obtained as precursors for glycoconjugates NIT72A and NIT72B (**Fig. 2**).

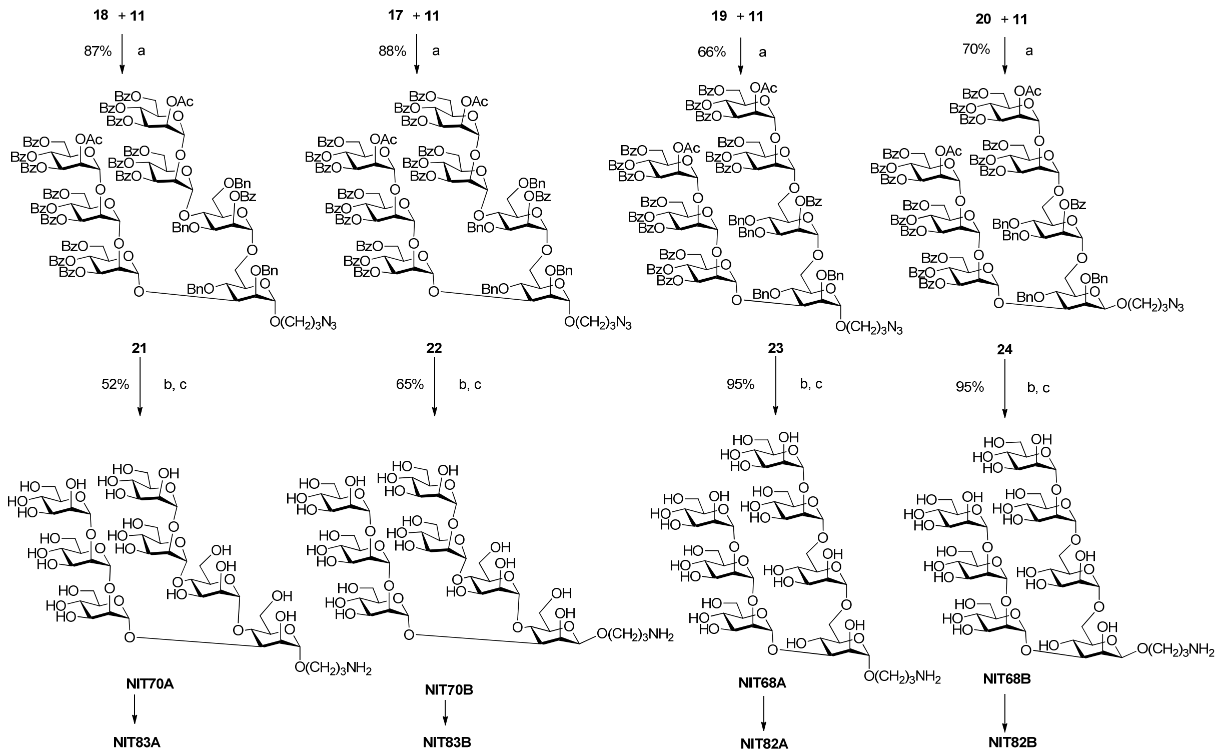

Reagents and conditions: a) TMSOTf, AW MS 4 Å, DCM; b) NaOMe, MeOH; c) Pd-C, H_2, MeOH/H_2O/AcOH.

Supplementary Figure 4 | Synthesis of mannoheptaosides. The pentasaccharide acceptor derivatives **17**, **18**, **19** and **20**, respectively, were then glycosylated using the disaccharide donor **11** (**Supplementary Fig. 1**) in good yields to afford heptasaccharides **21** - **24**, which were then globally deprotected to furnish the aminopropyl derivatives **NIT70A**, **NIT70B**, **NIT68A** and **NIT68B**, respectively, as precursors of the neoglycoconjugates **NIT83A**, **NIT83B**, **NIT82A** and **NIT82B** (**Fig. 2**). All target compounds were purified by LH-20 chromatography and fully characterized by NMR and mass spectrometric analyses (**Supplementary Fig. 5-26**).

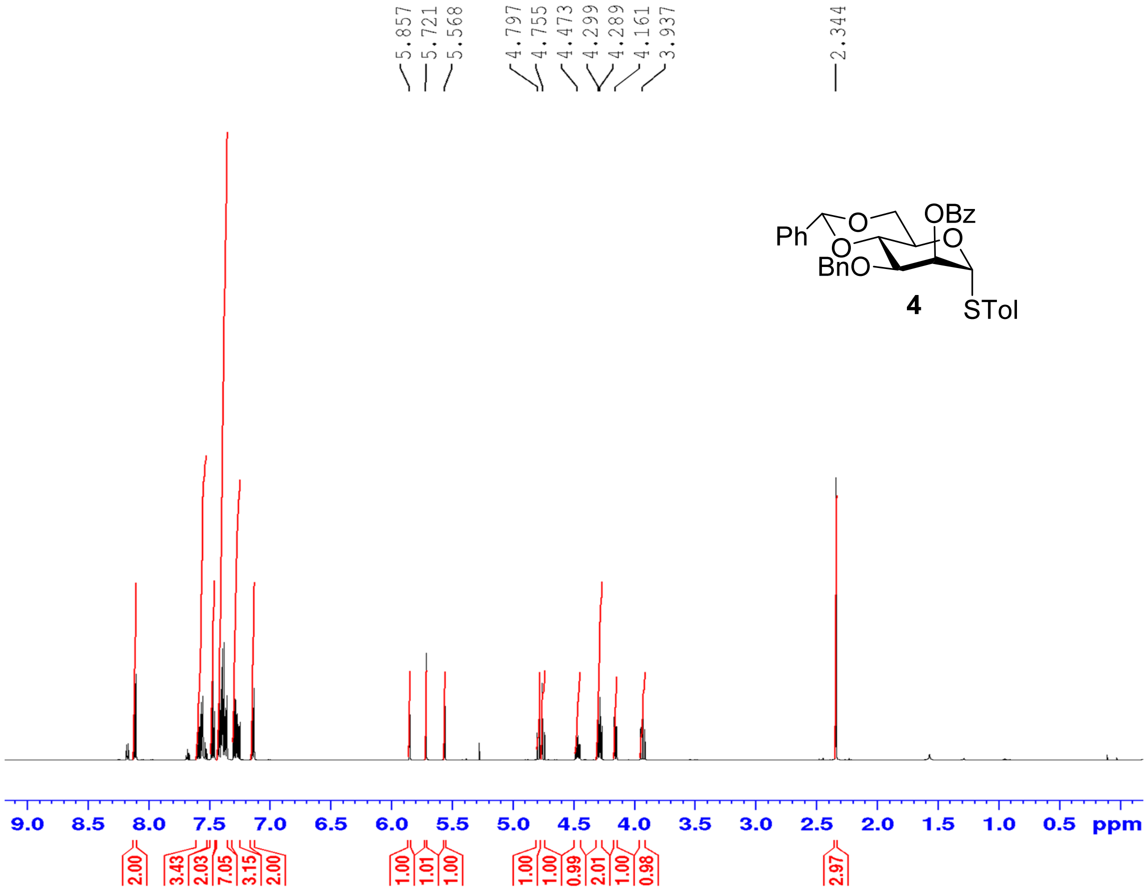

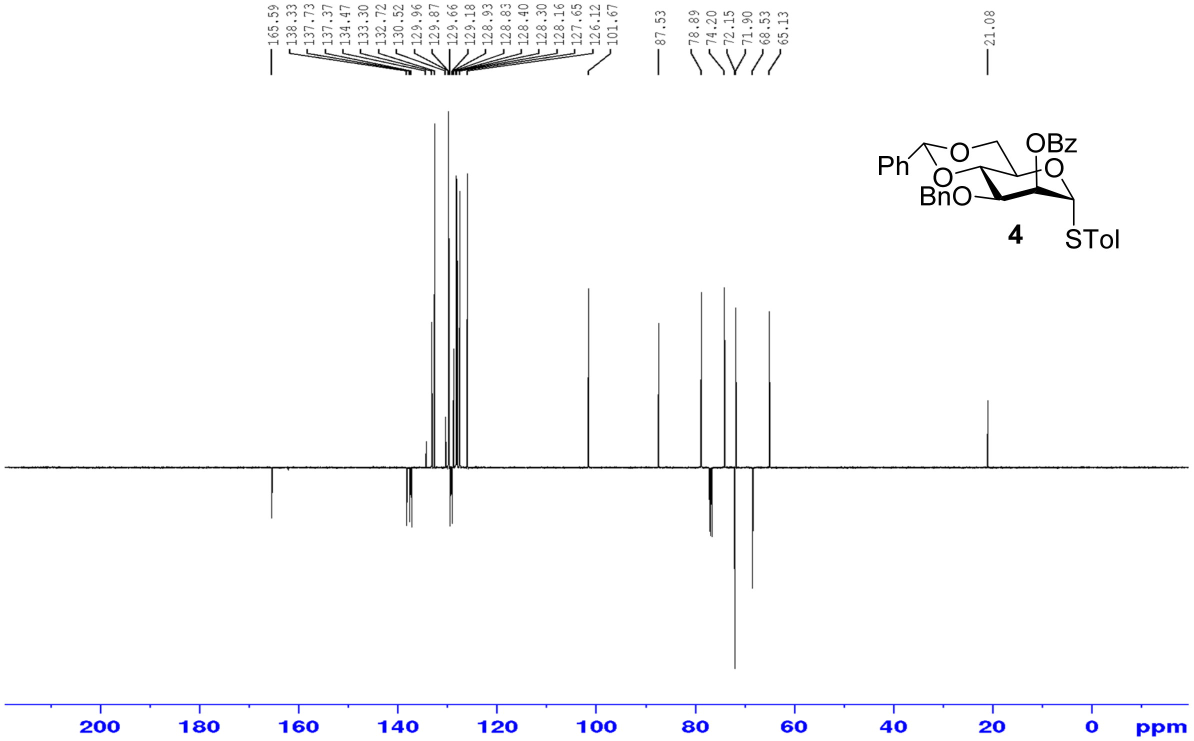

Supplementary Figure 5 | ^{1}H NMR spectrum (top) and ^{13}C NMR spectrum (bottom) of **4**.

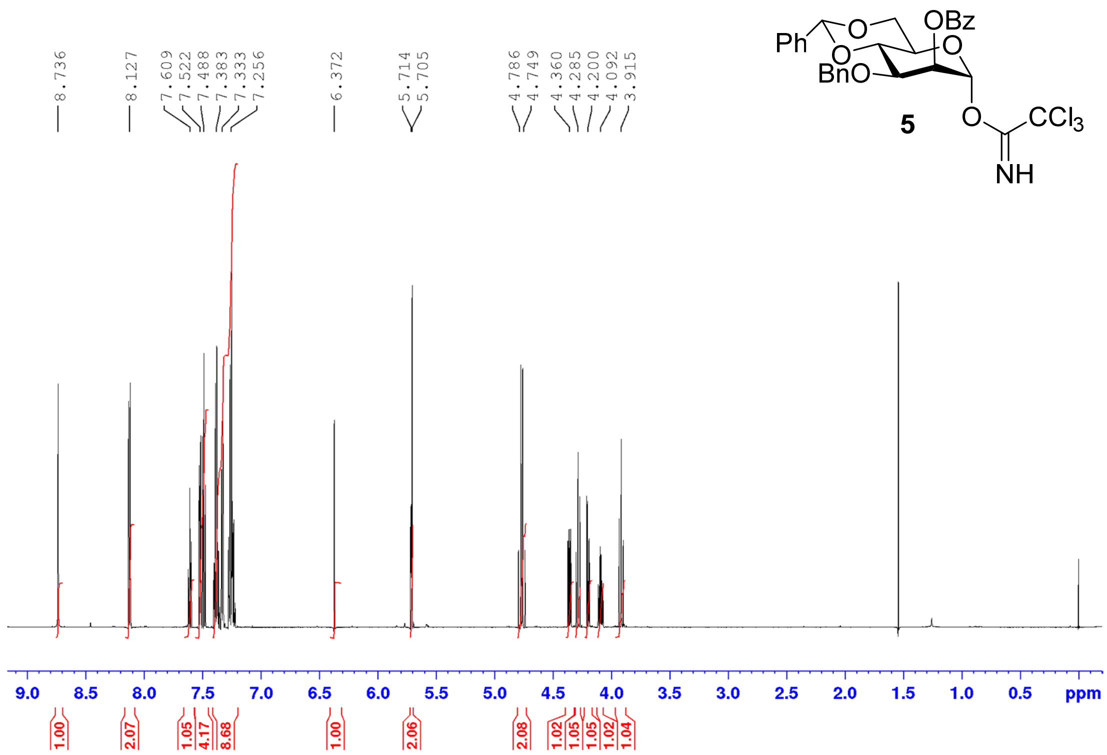

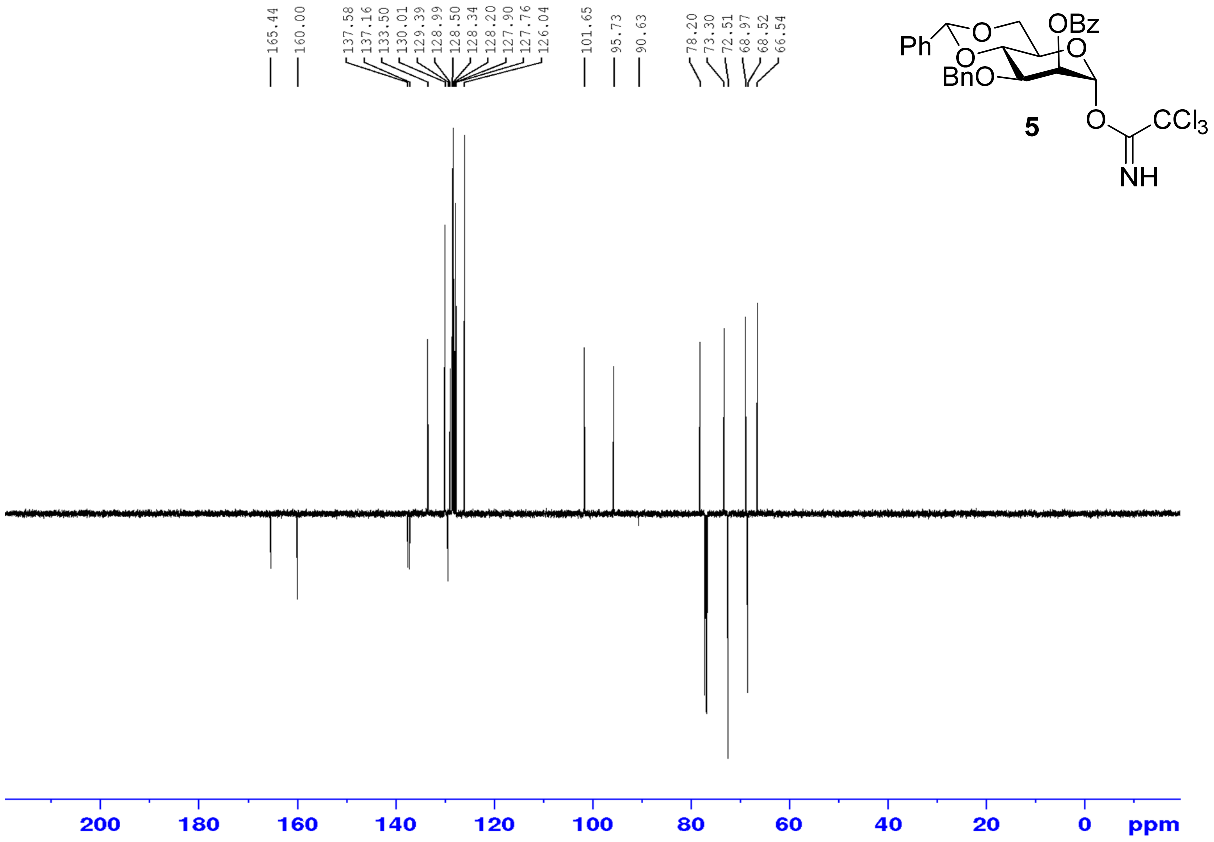

Supplementary Figure 6 | ^{1}H NMR spectrum (top) and ^{13}C NMR spectrum (bottom) of **5**.

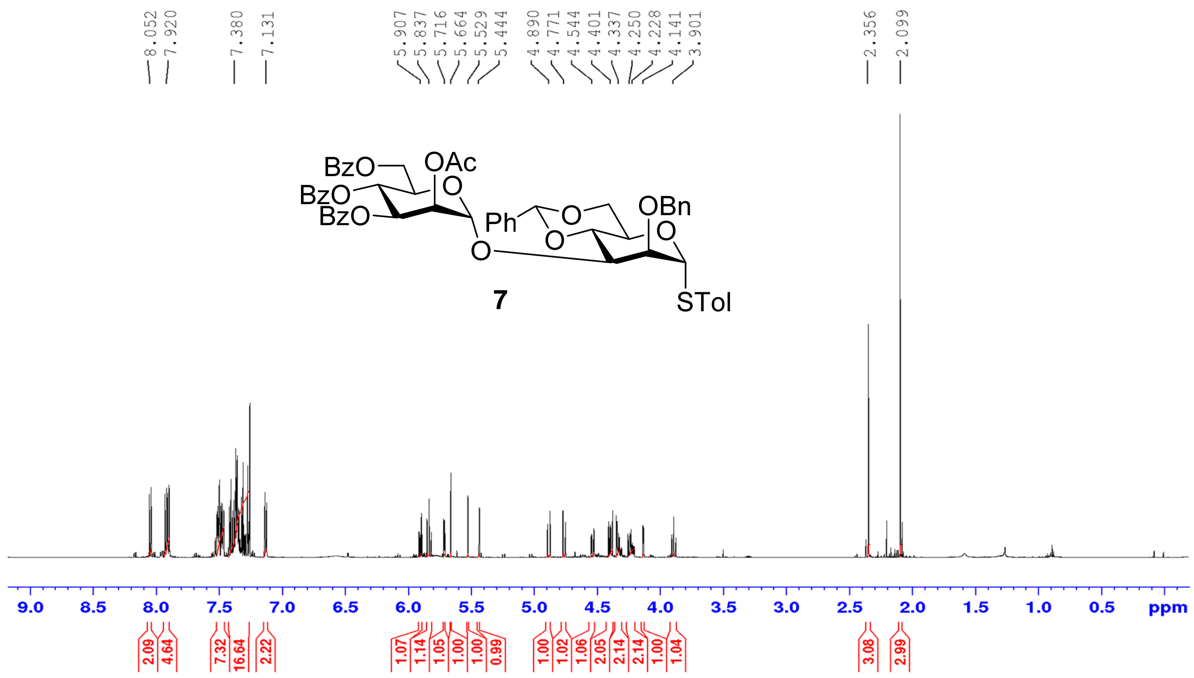

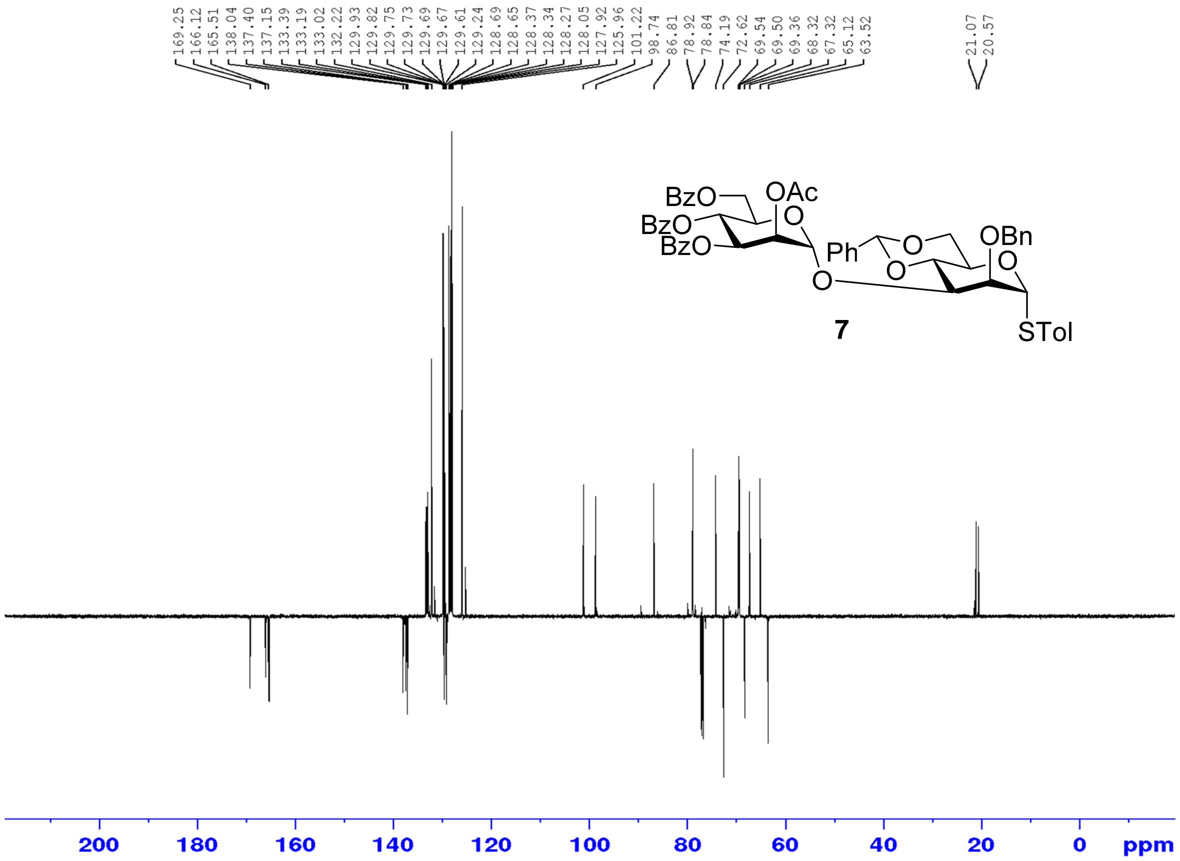

Supplementary Figure 7 | ^{1}H NMR spectrum (top) and ^{13}C NMR spectrum (bottom) of **7**.

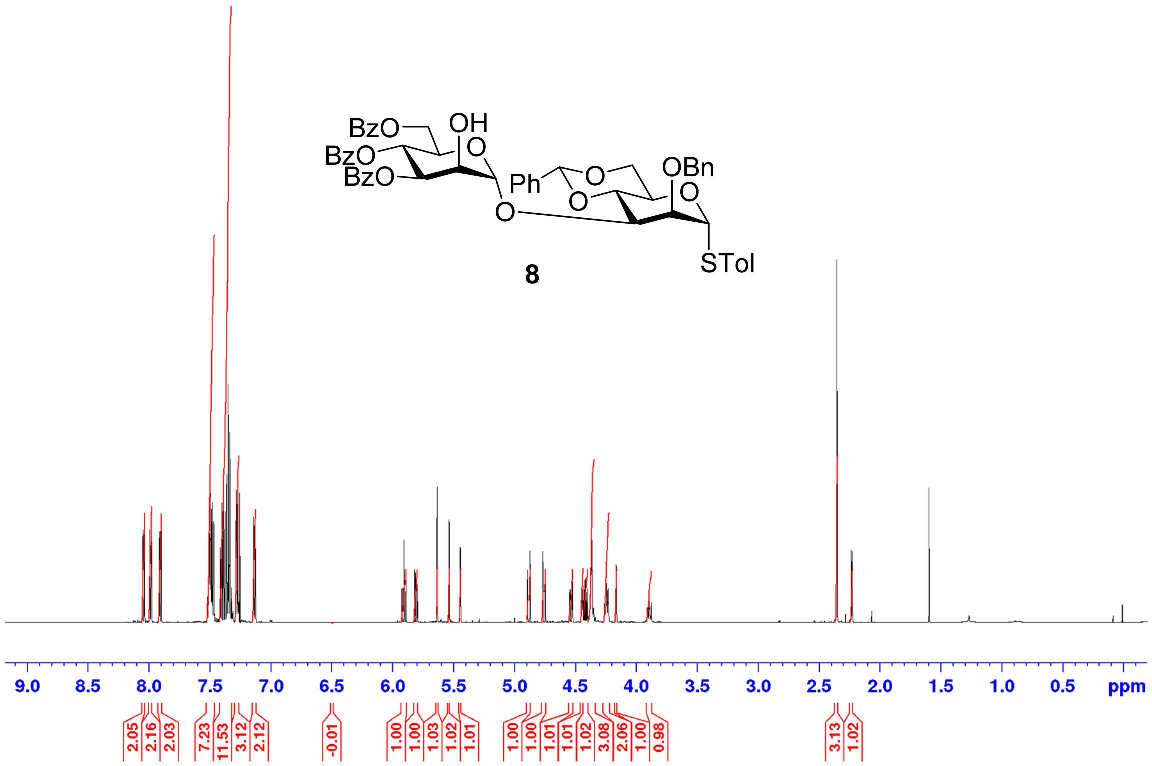

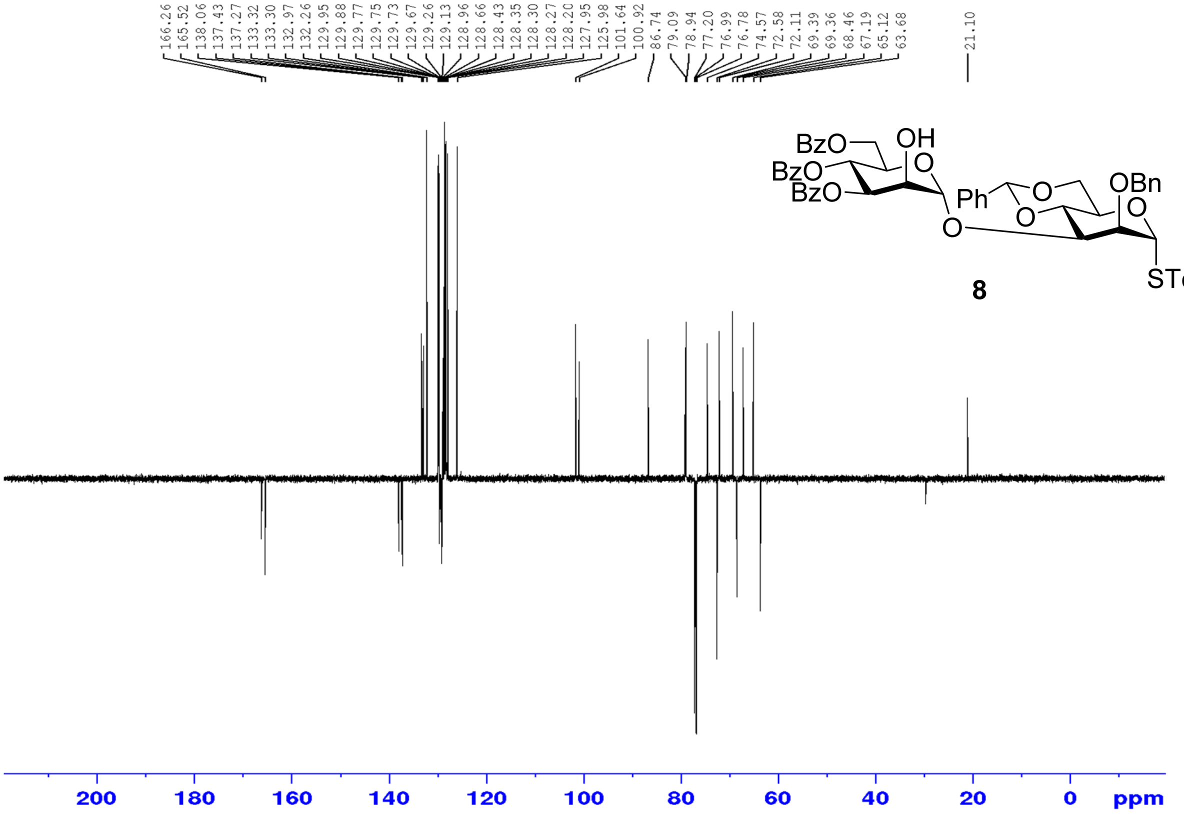

Supplementary Figure 8 | ^{1}H NMR spectrum (top) and ^{13}C NMR spectrum (bottom) of **8**.

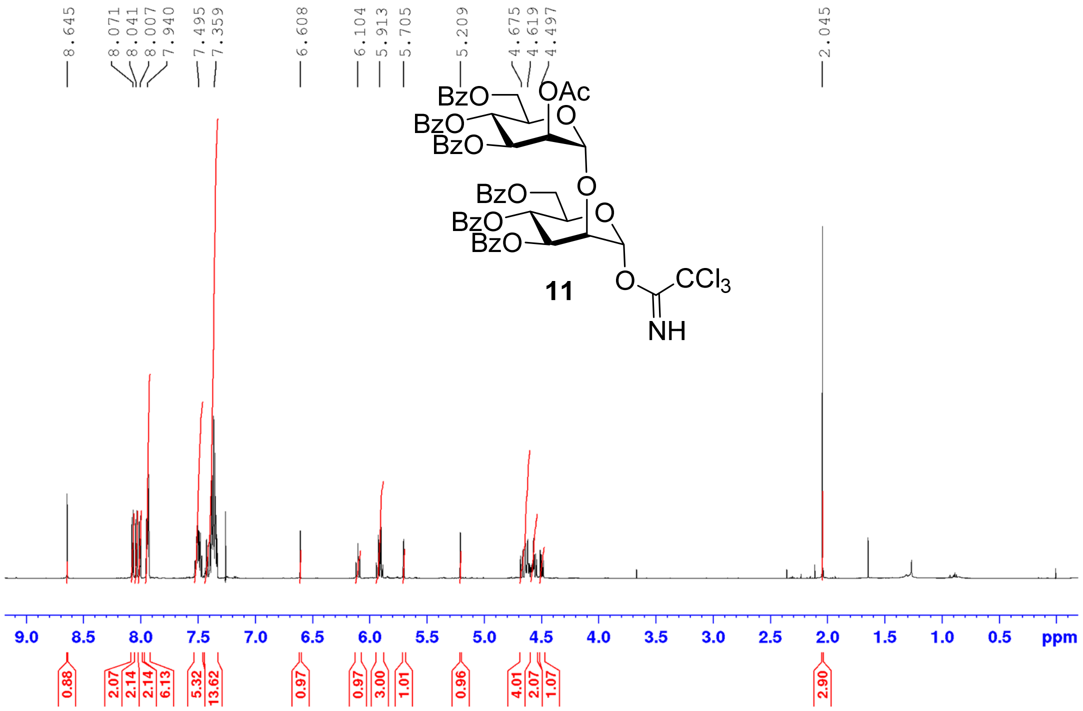

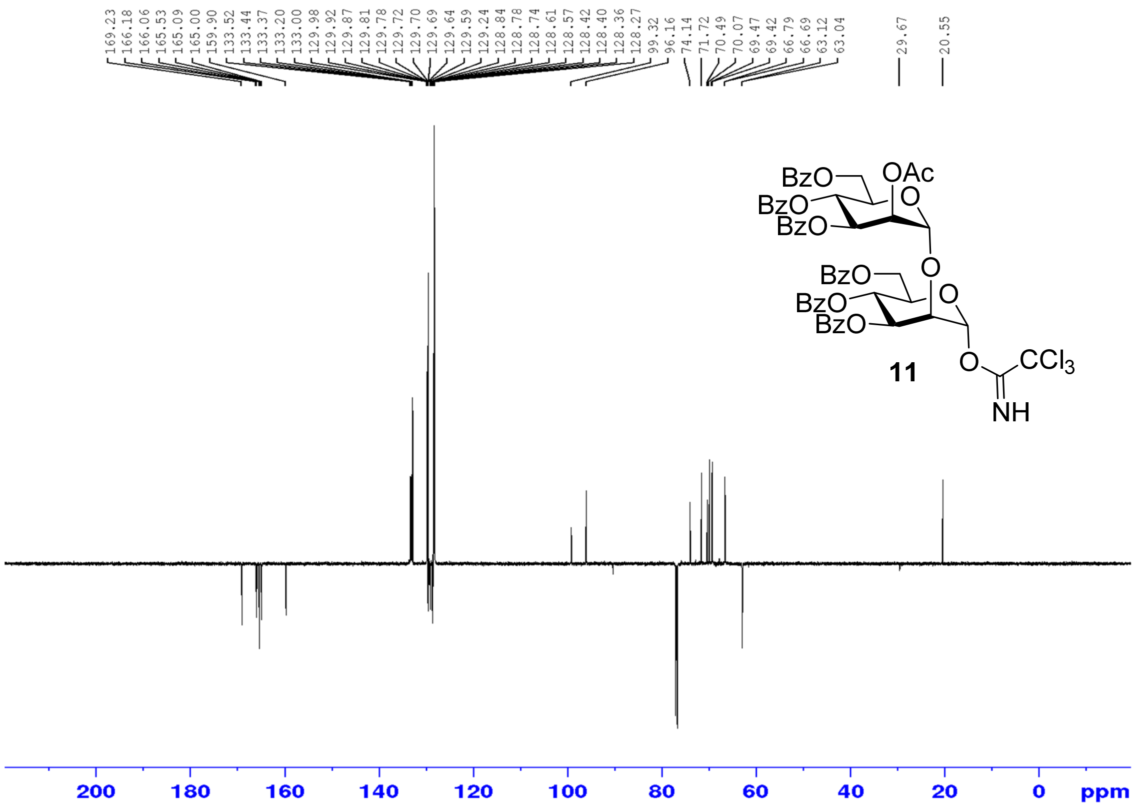

Supplementary Figure 9 | ^{1}H NMR spectrum (top) ^{13}C NMR spectrum (bottom) of **11**.

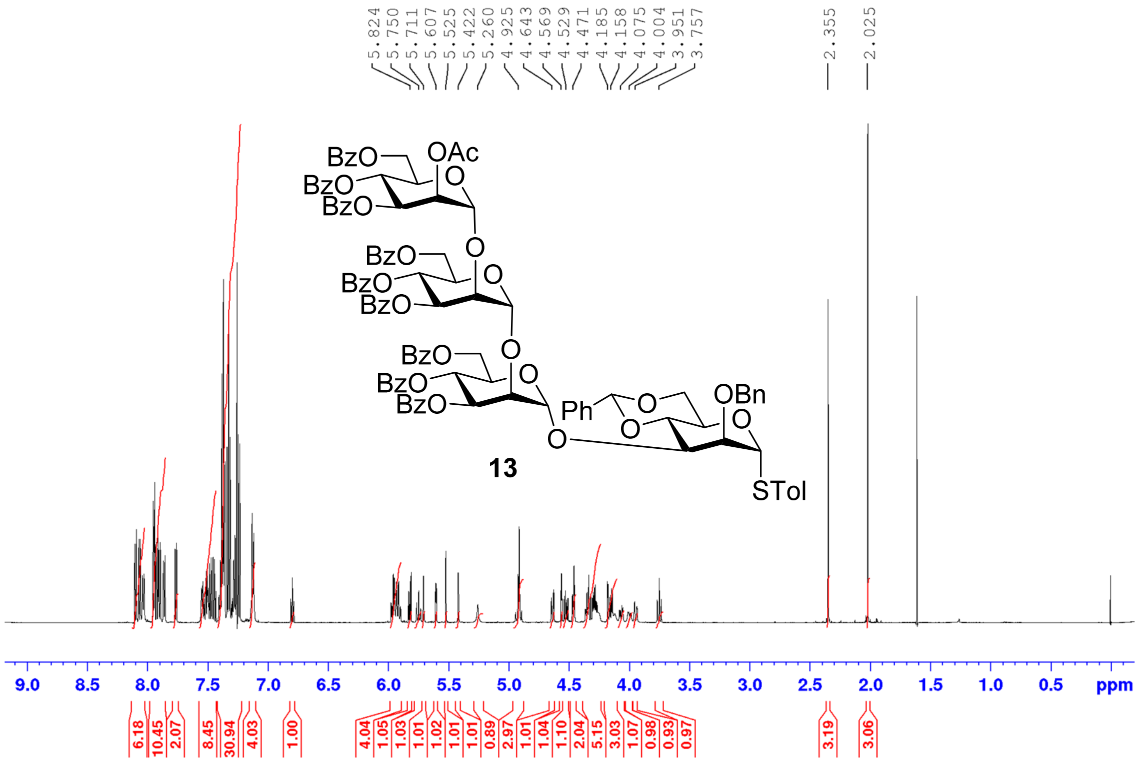

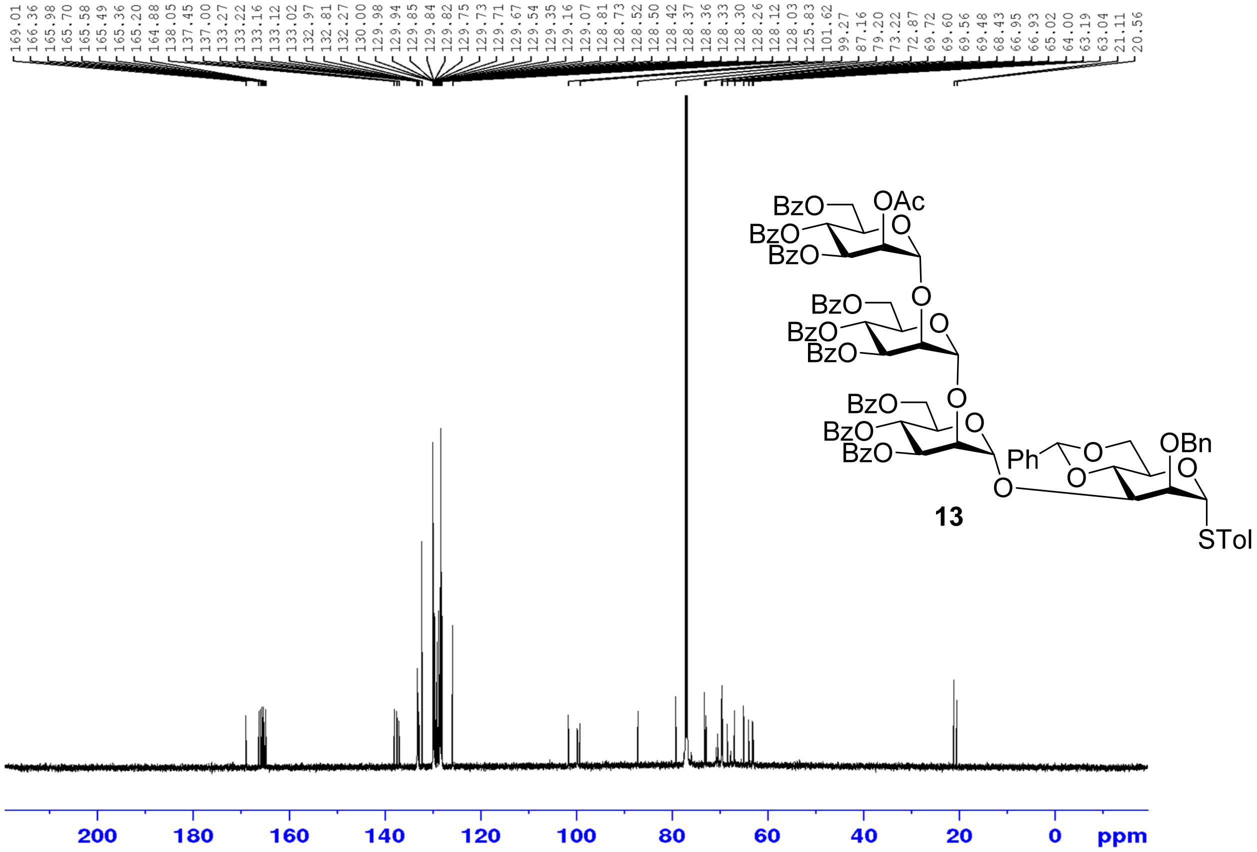

Supplementary Figure 10 | ^{1}H NMR spectrum (top) and ^{13}C NMR spectrum (bottom) of **13**.

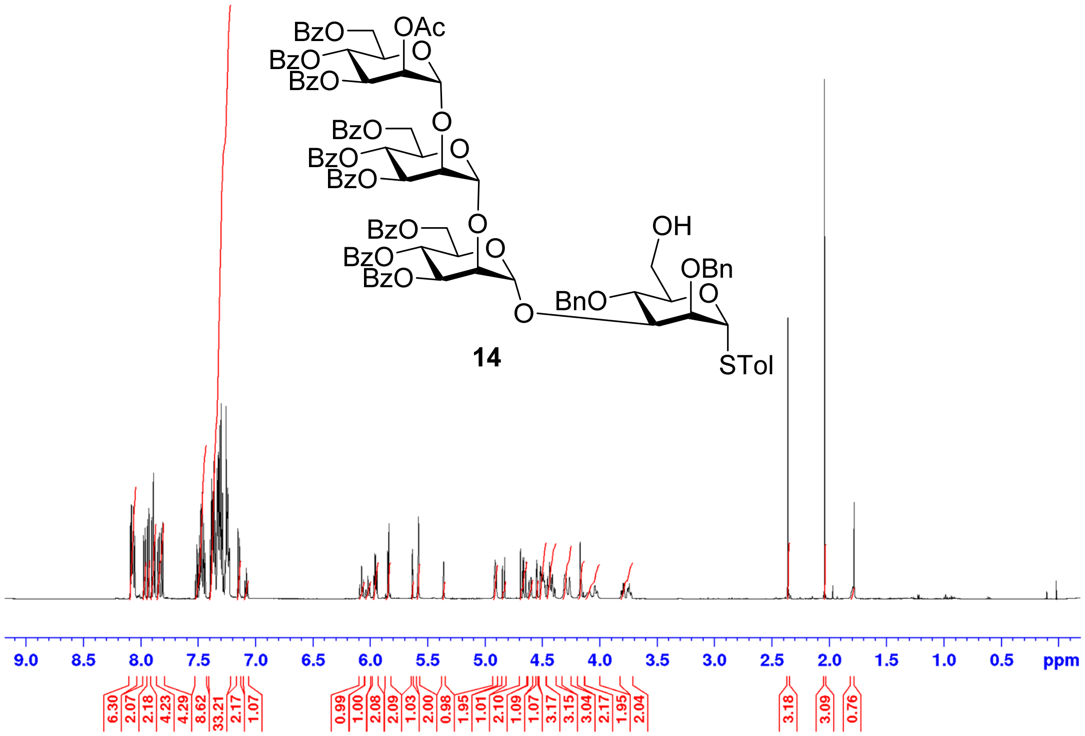

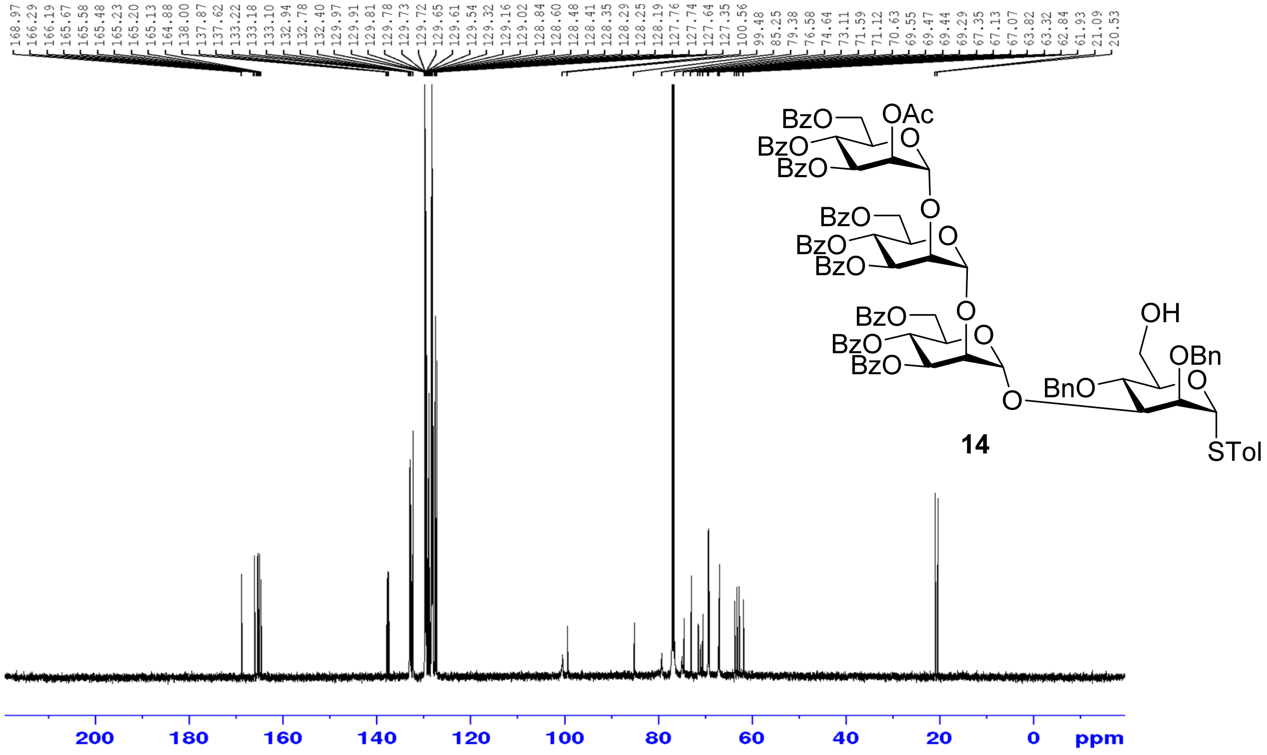

Supplementary Figure 11 | ^{1}H NMR spectrum (top) and ^{13}C NMR spectrum (bottom) of **14**.

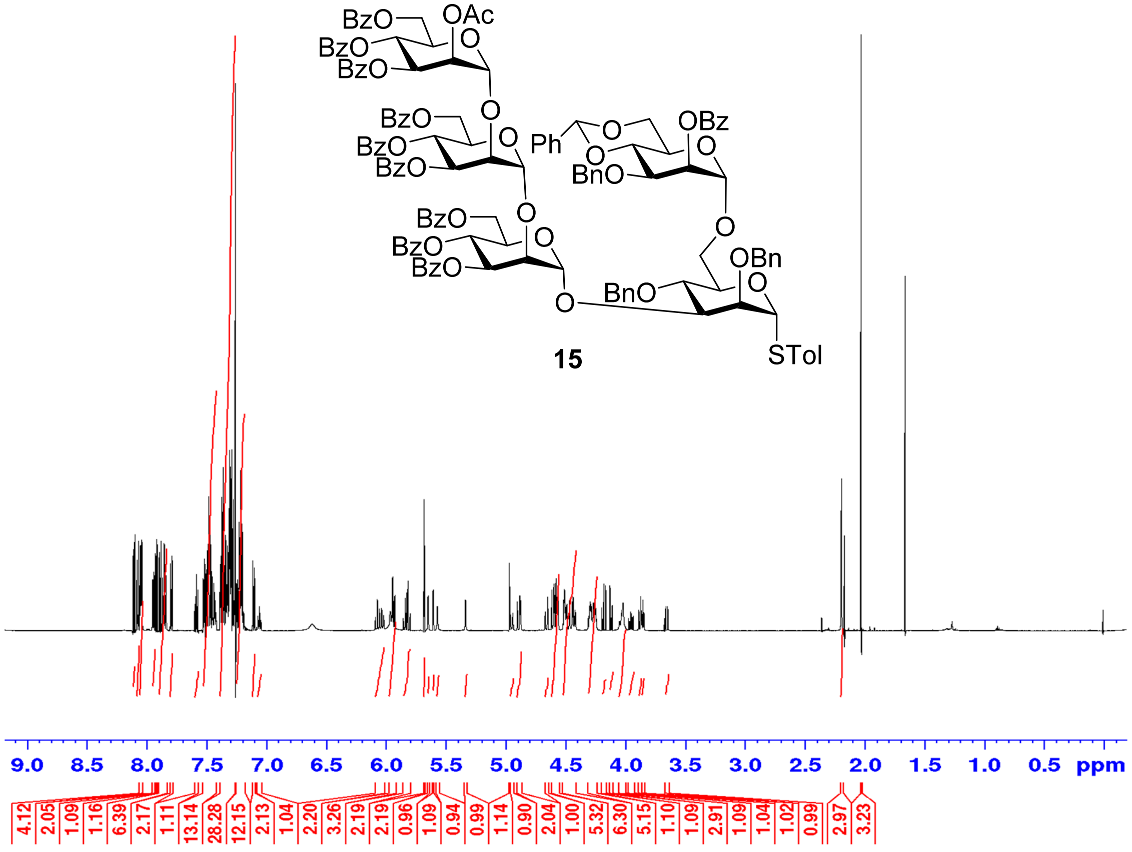

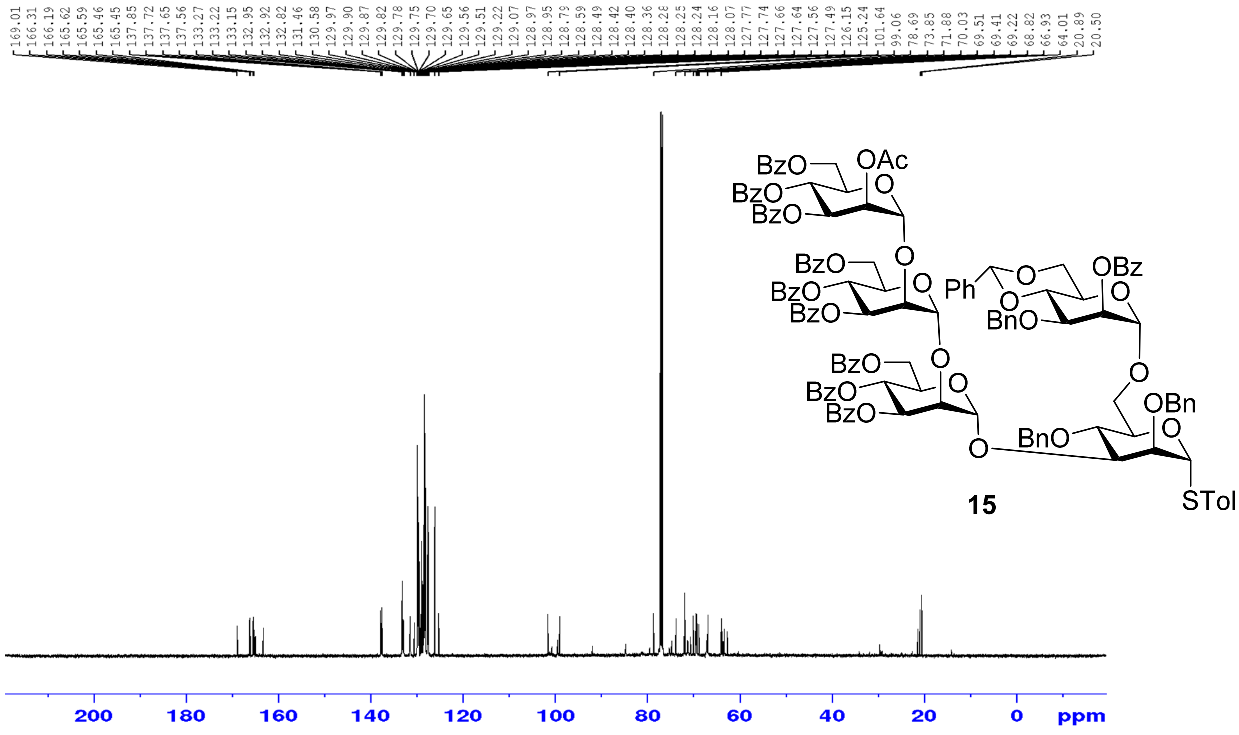

Supplementary Figure 12 | ^{1}H NMR spectrum (top) and ^{13}C NMR spectrum (bottom) of **15**.

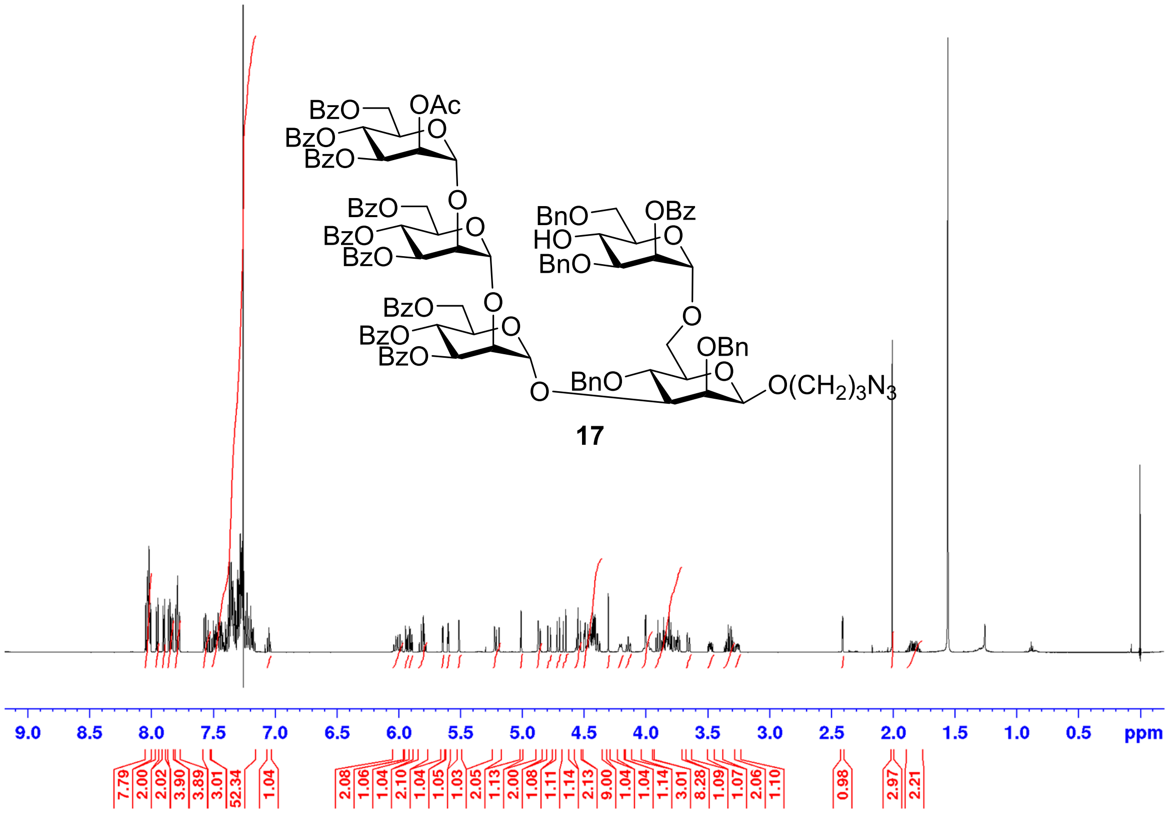

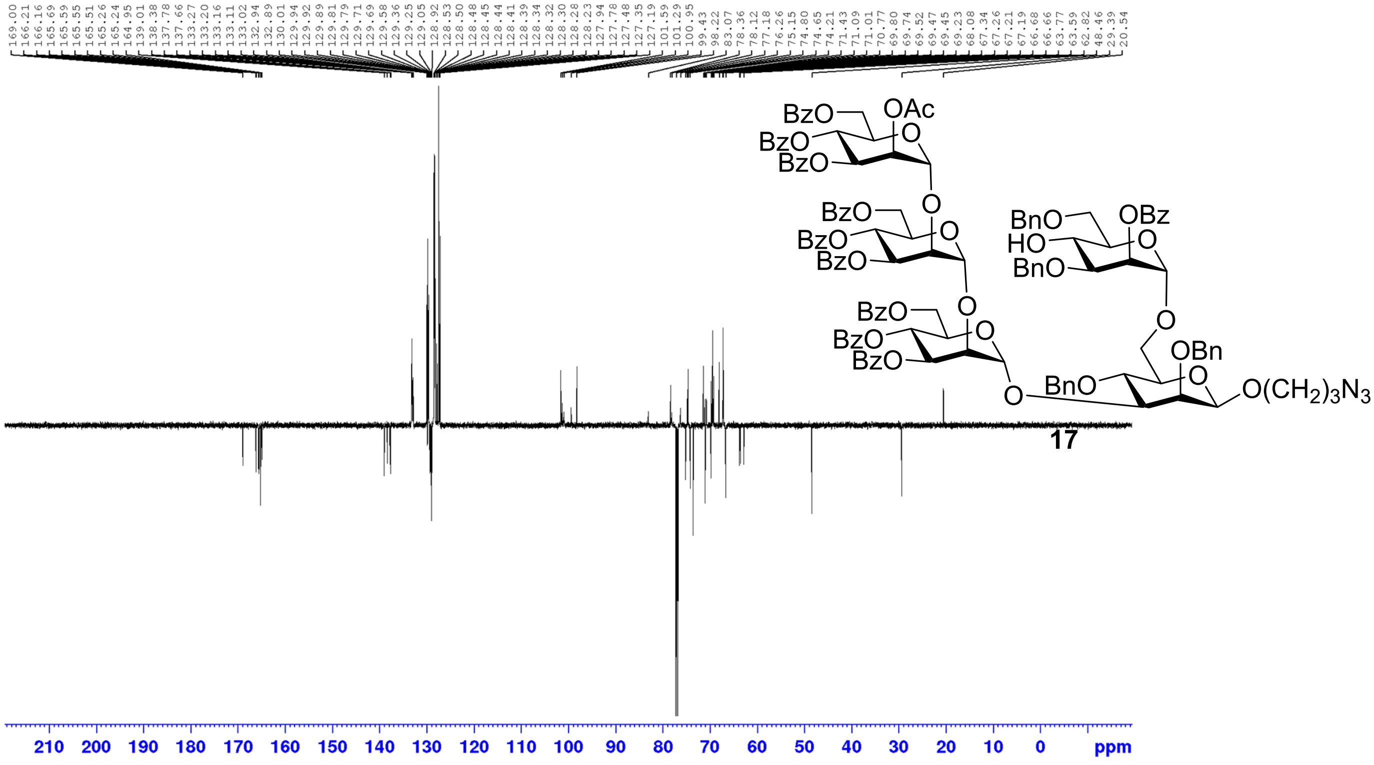

Supplementary Figure 13 | ^{1}H NMR spectrum (top) and ^{13}C NMR spectrum (bottom) of **17**.

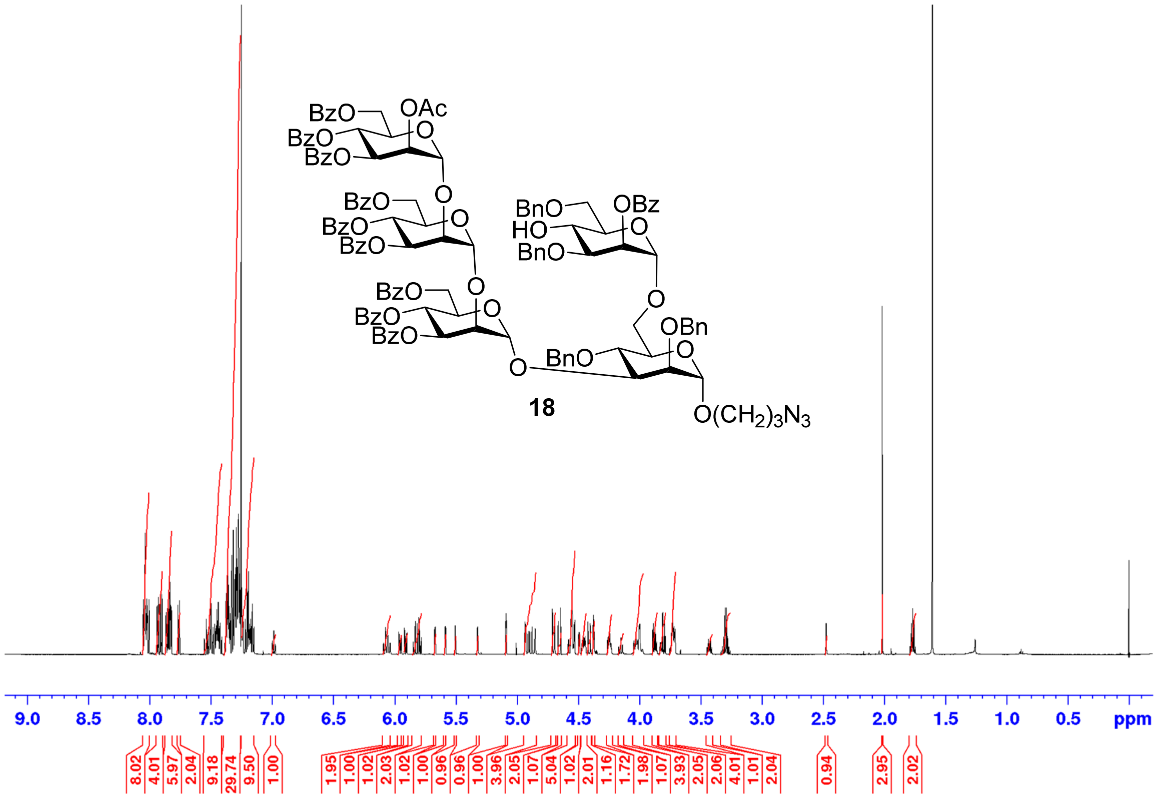

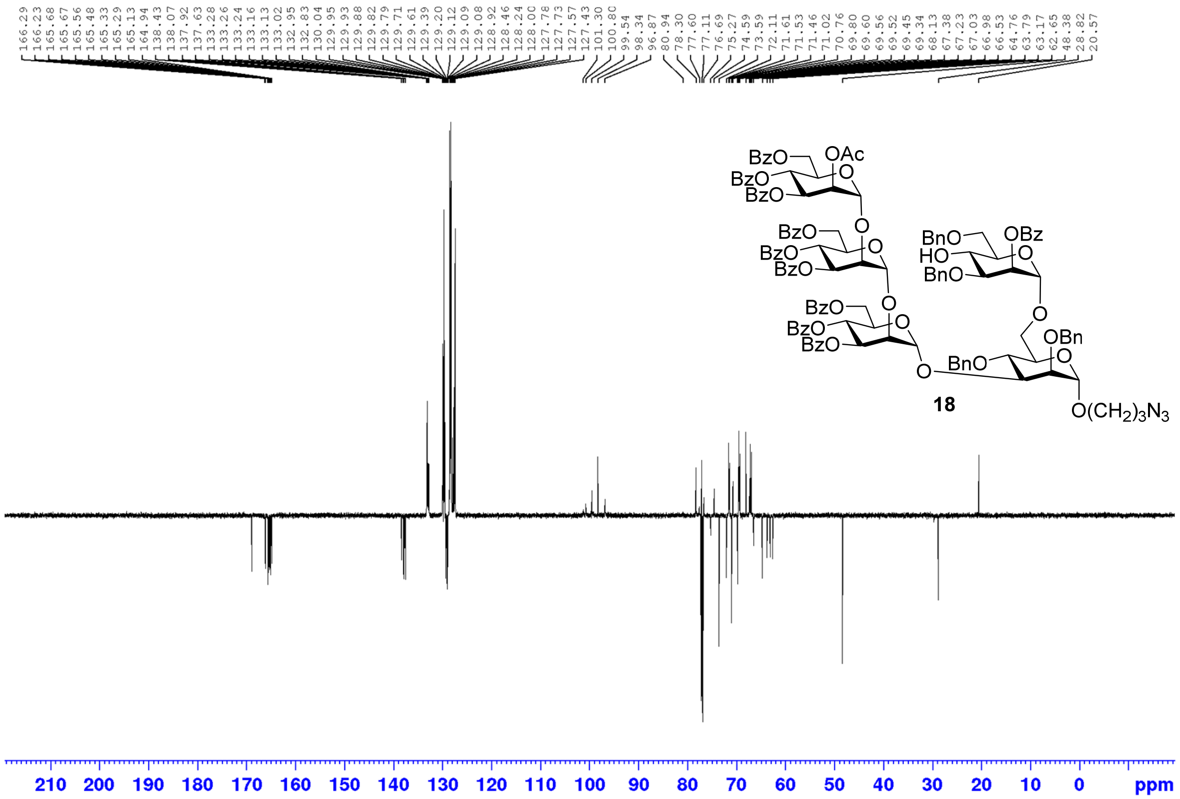

Supplementary Figure 14 | ^{1}H NMR spectrum (top) and ^{13}C NMR spectrum (bottom) of **18**.

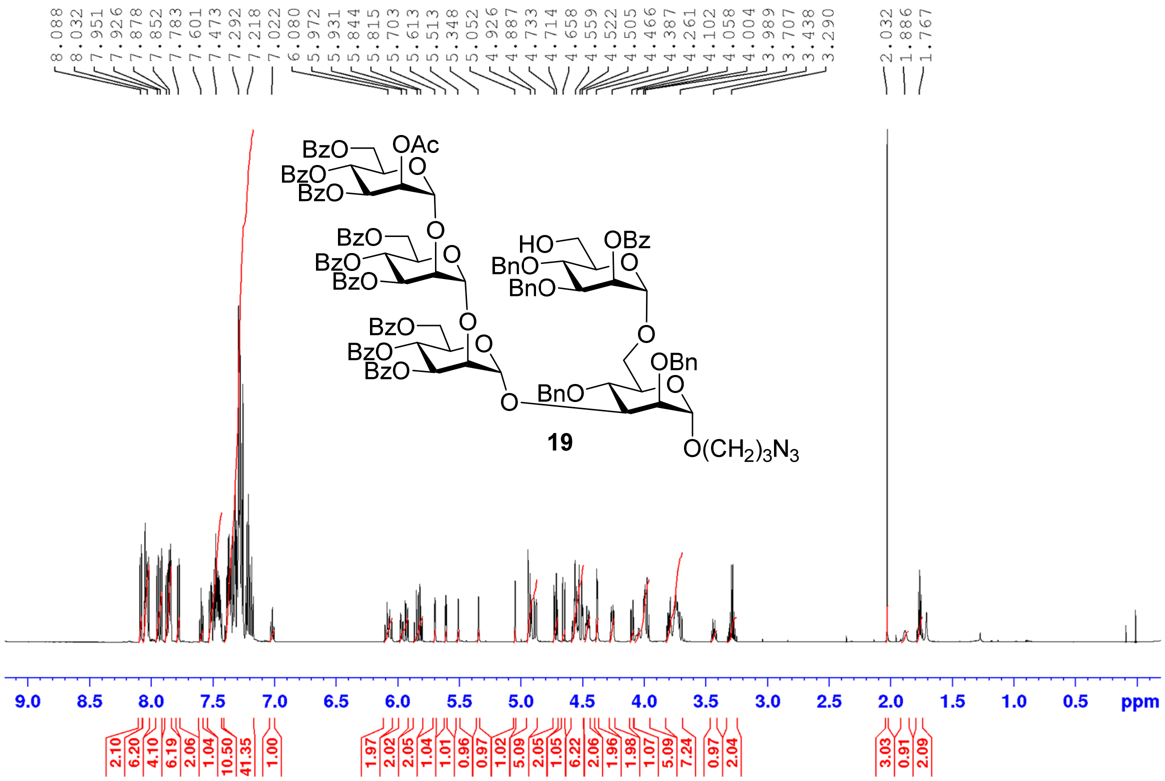

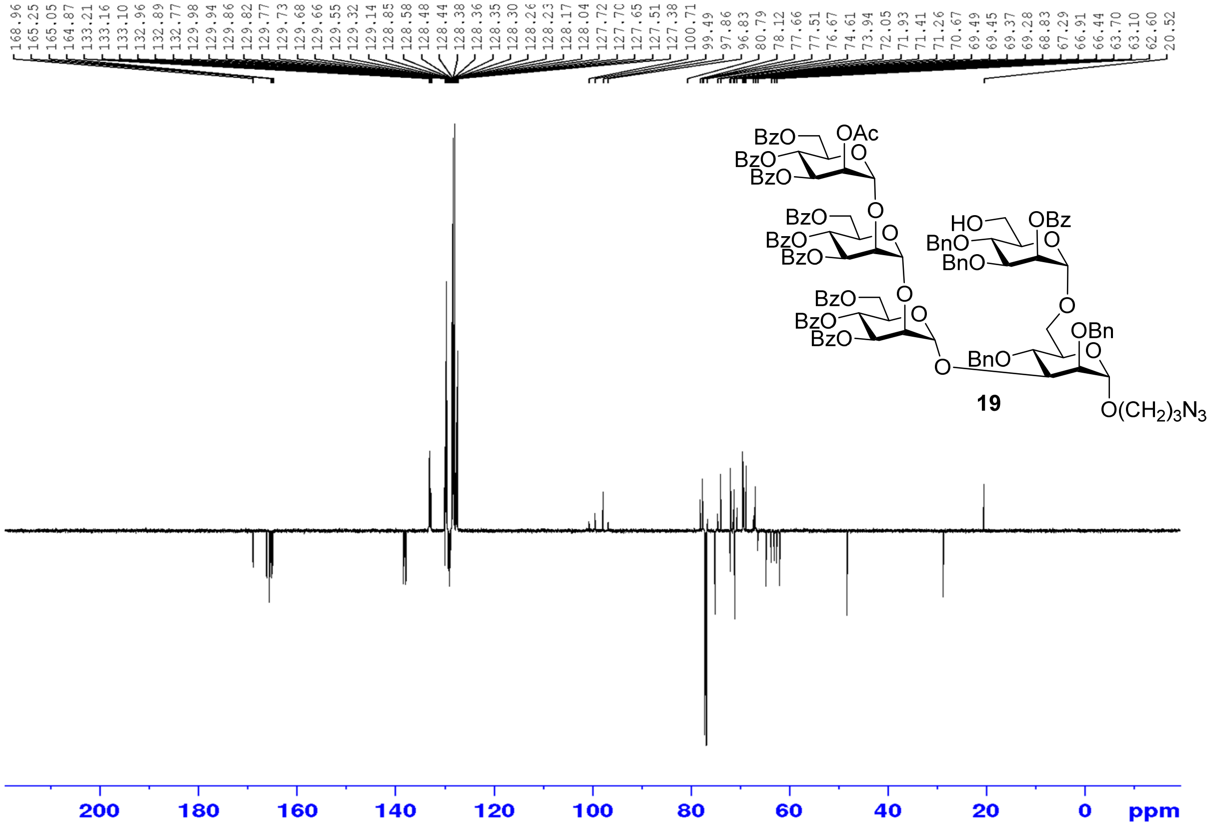

Supplementary Figure 15 | ^{1}H NMR spectrum (top) and ^{13}C NMR spectrum (bottom) of **19**.

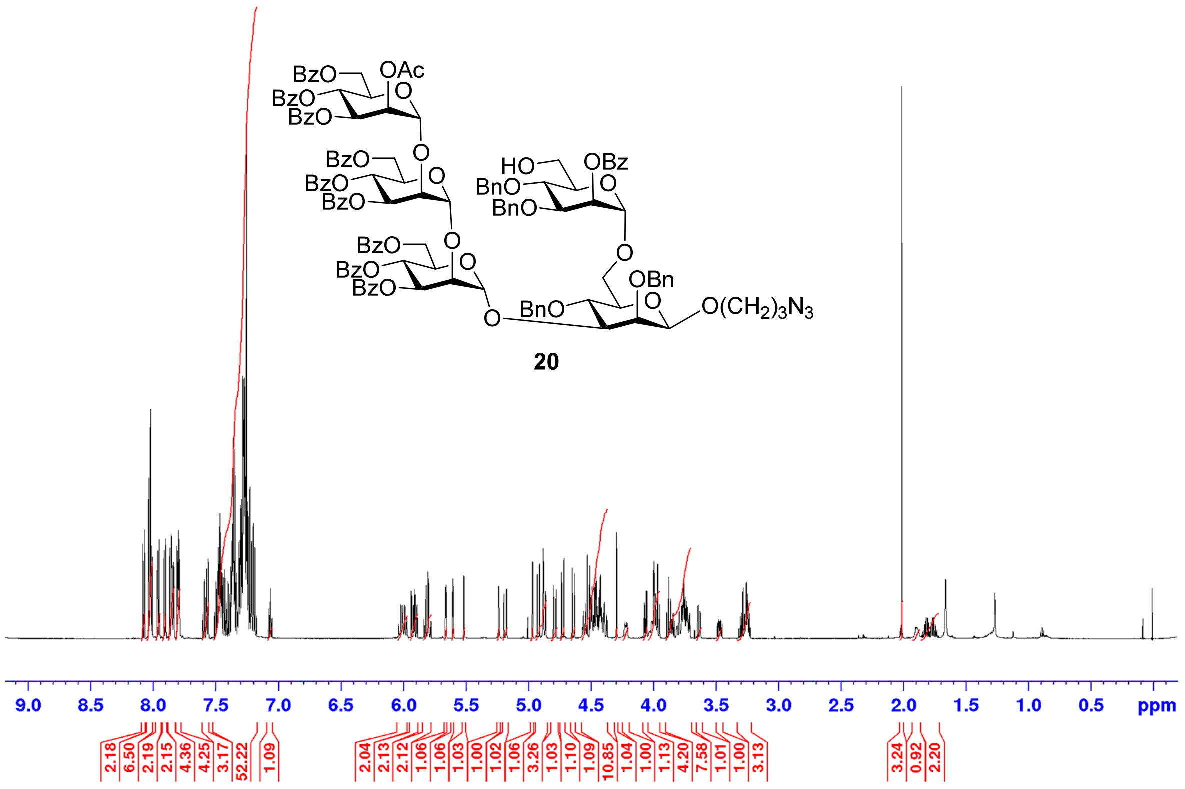

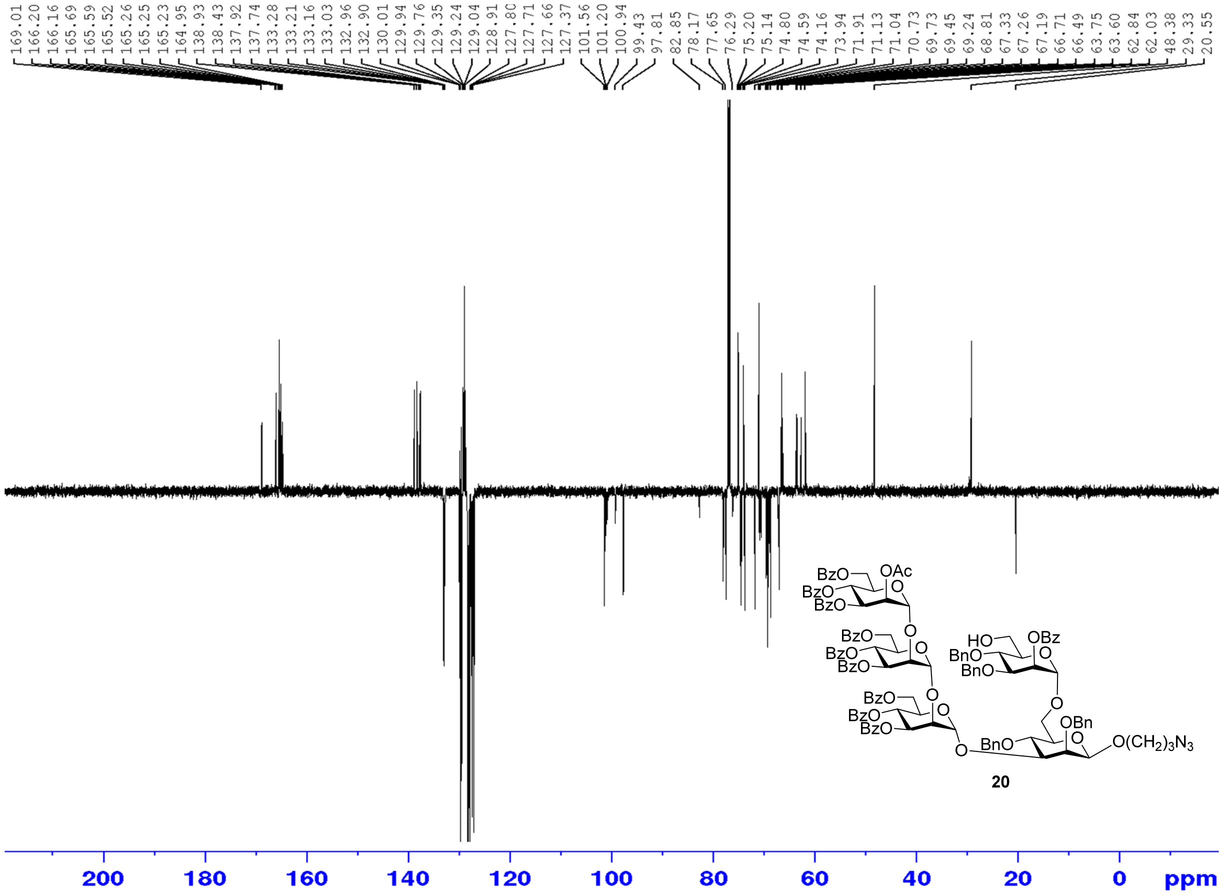

Supplementary Figure 16 | ^{1}H NMR spectrum (top) and ^{13}C NMR spectrum (bottom) of **20**.

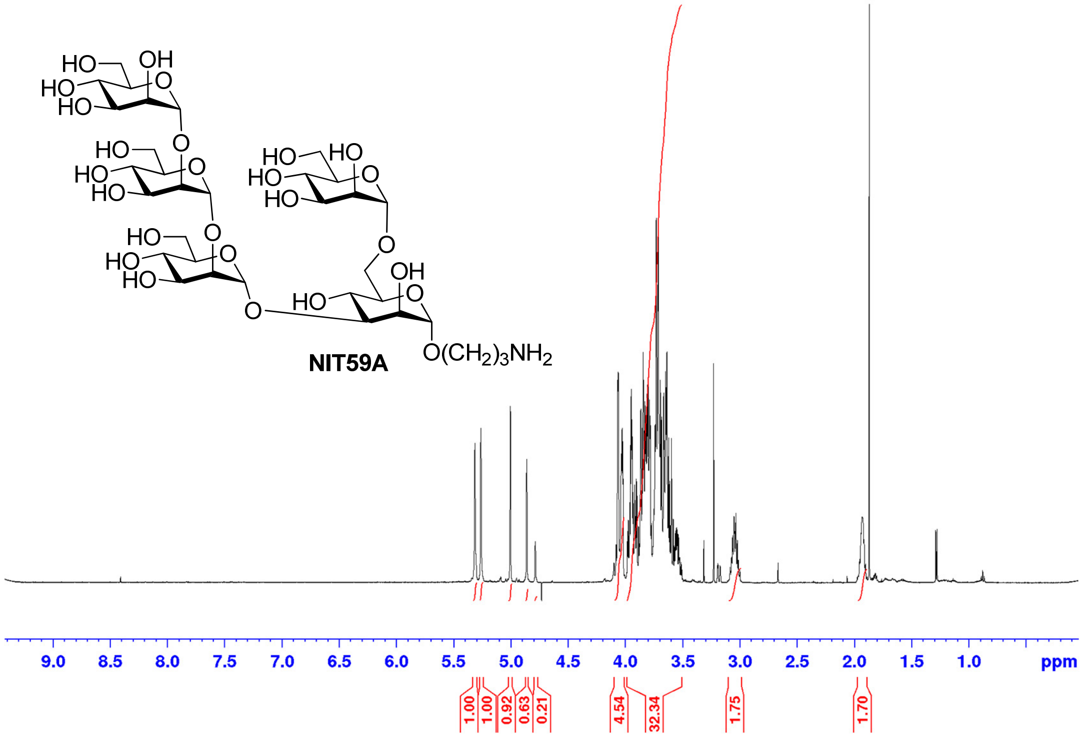

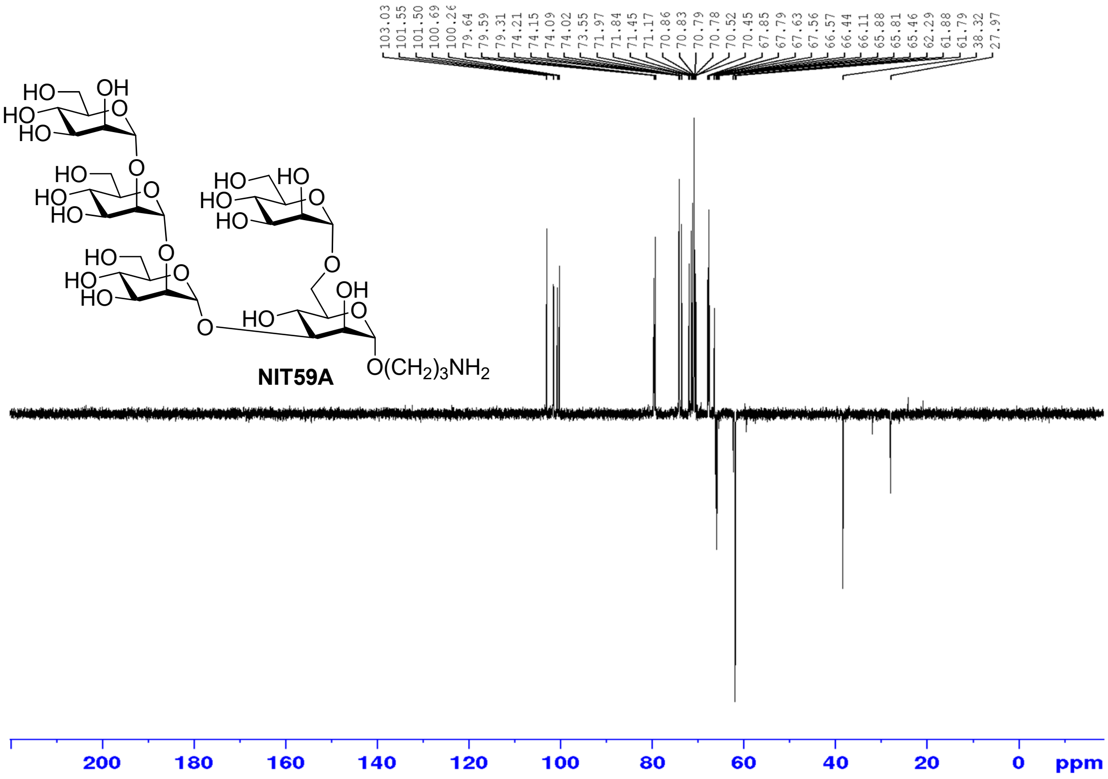

Supplementary Figure 17 | ^{1}H NMR spectrum (top) and ^{13}C NMR spectrum (bottom) of **NIT59A**.

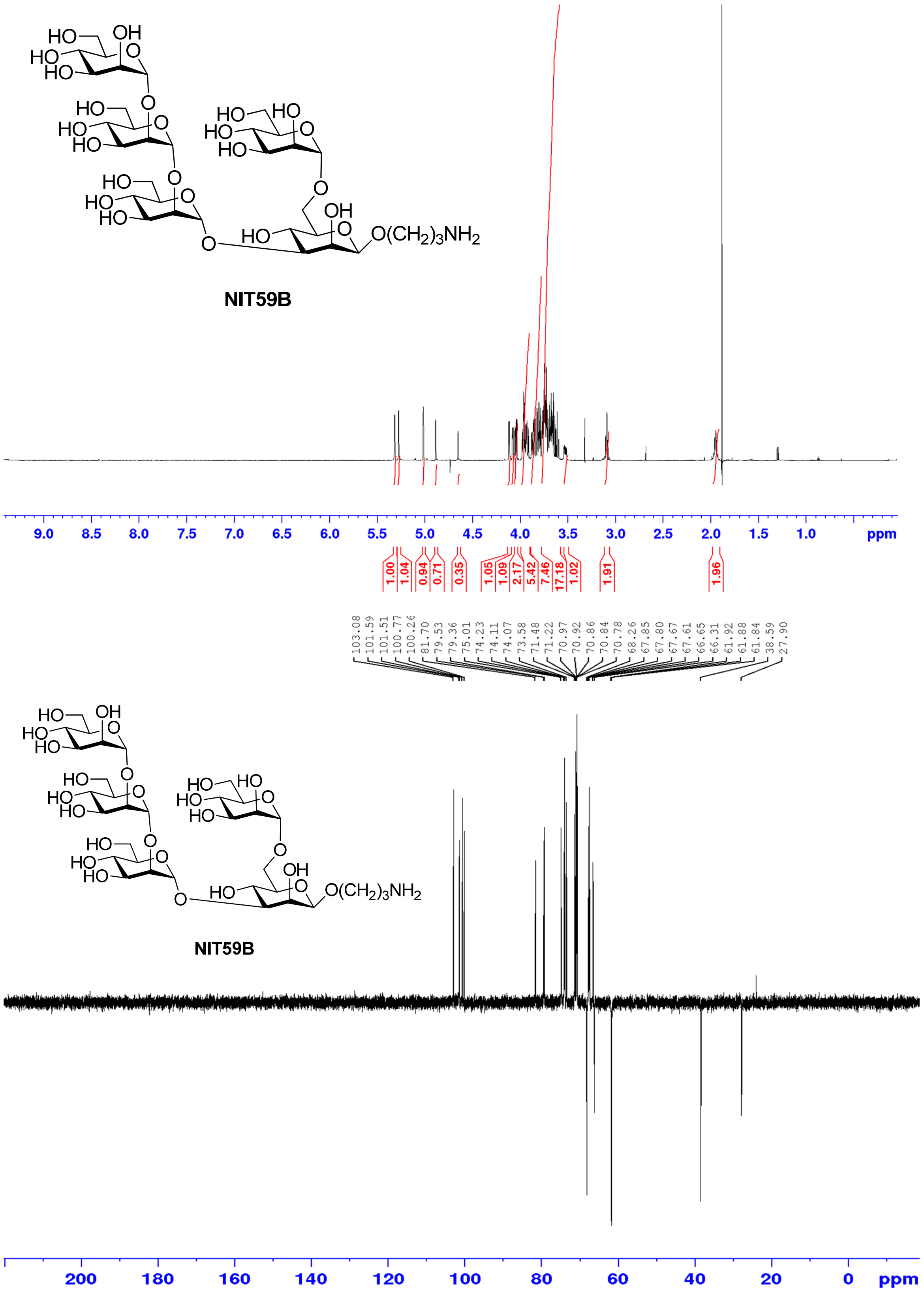

Supplementary Figure 18 | ^{1}H NMR spectrum (top) and ^{13}C NMR spectrum (bottom) of **NIT59B**.

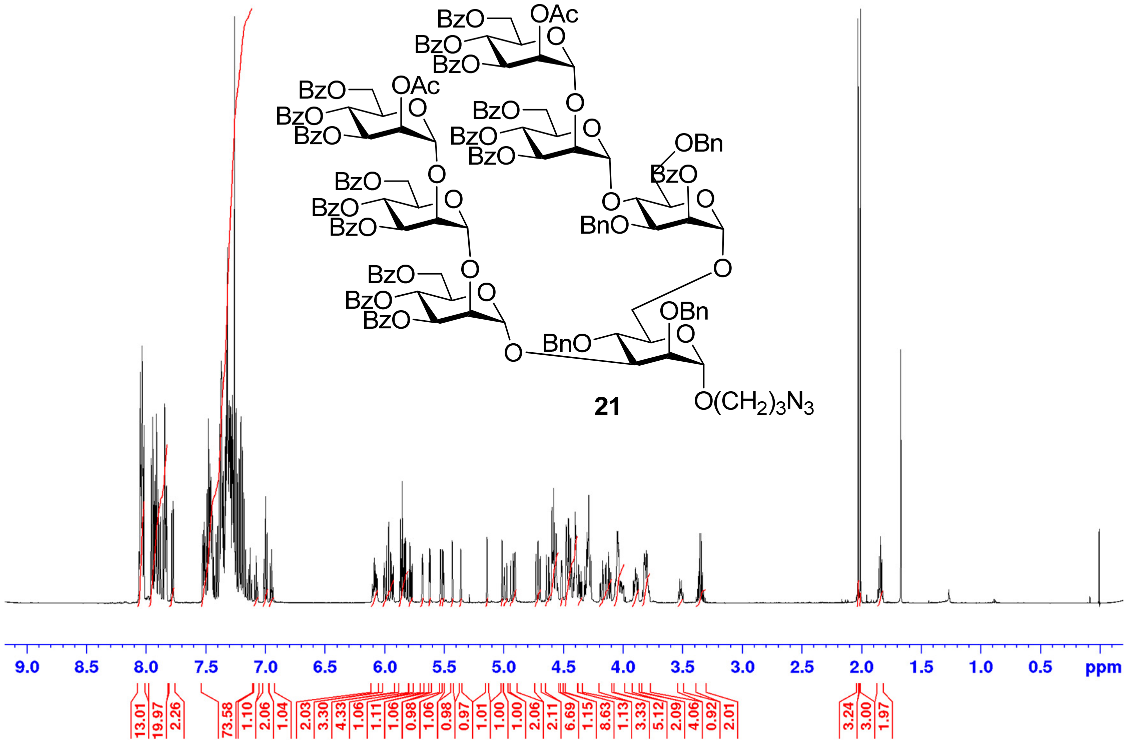

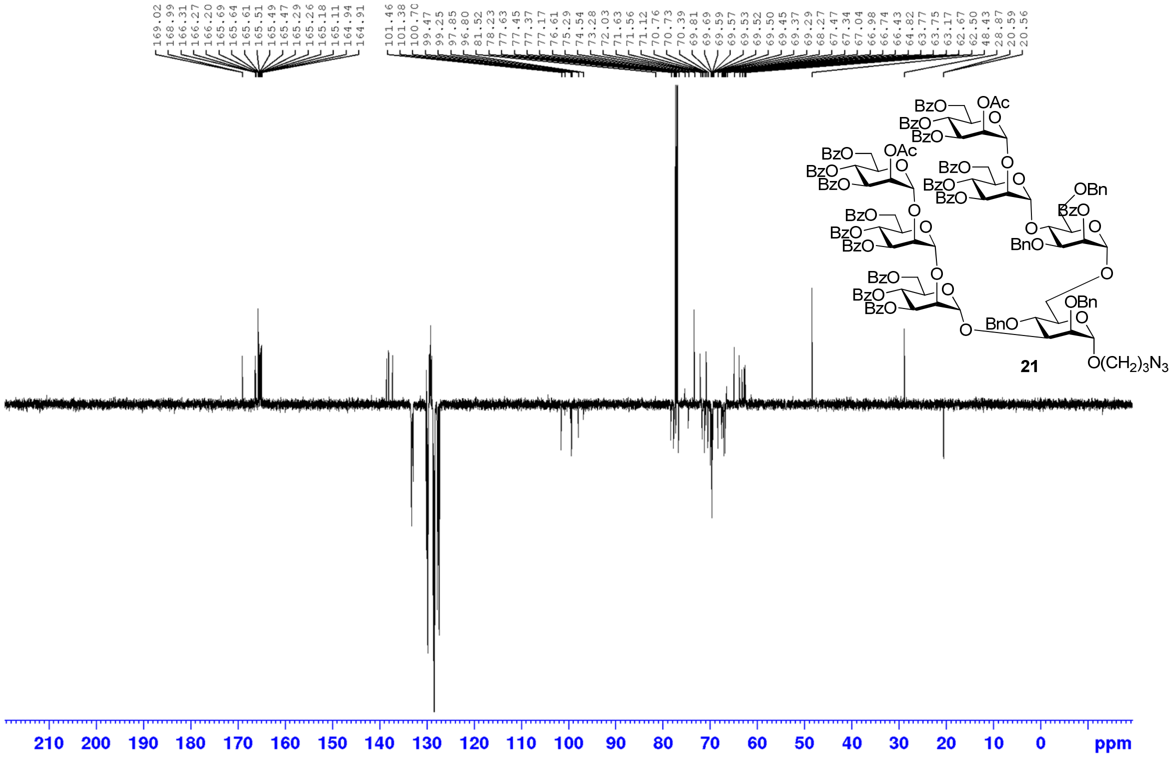

Supplementary Figure 19 | ^{1}H NMR spectrum (top) and ^{13}C NMR spectrum (bottom) of **21**.

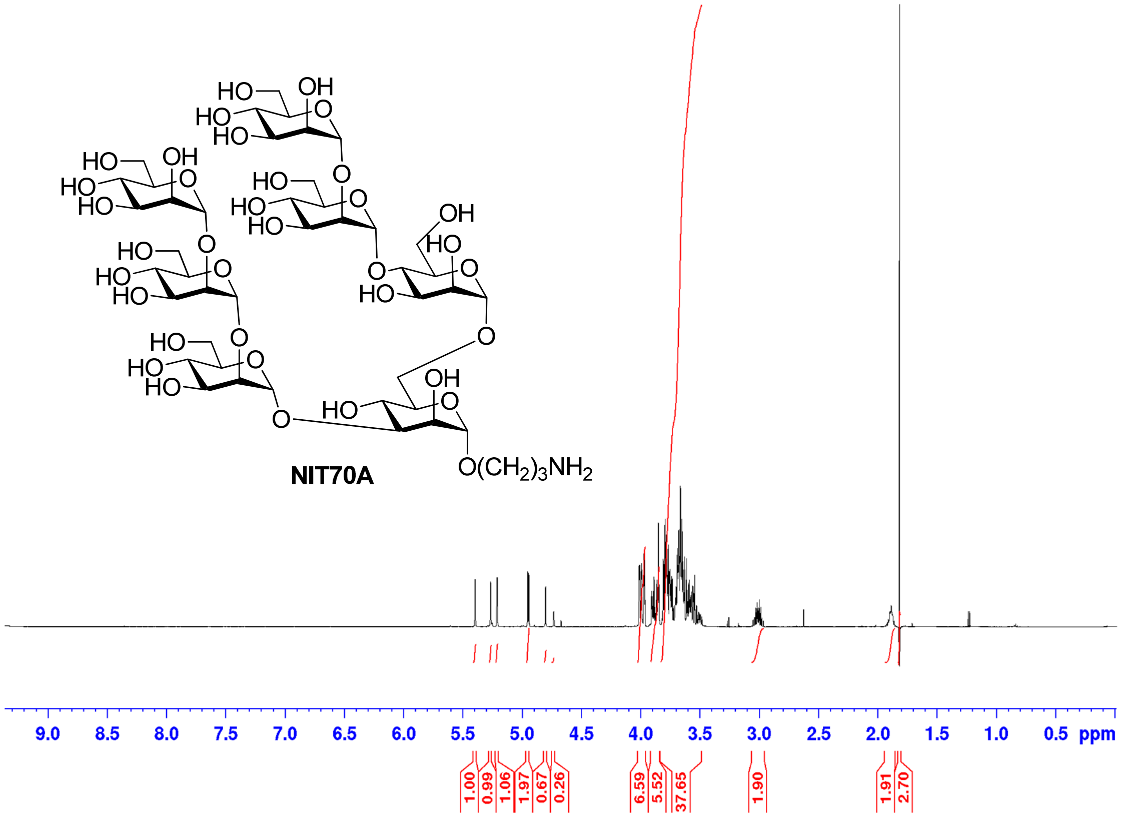

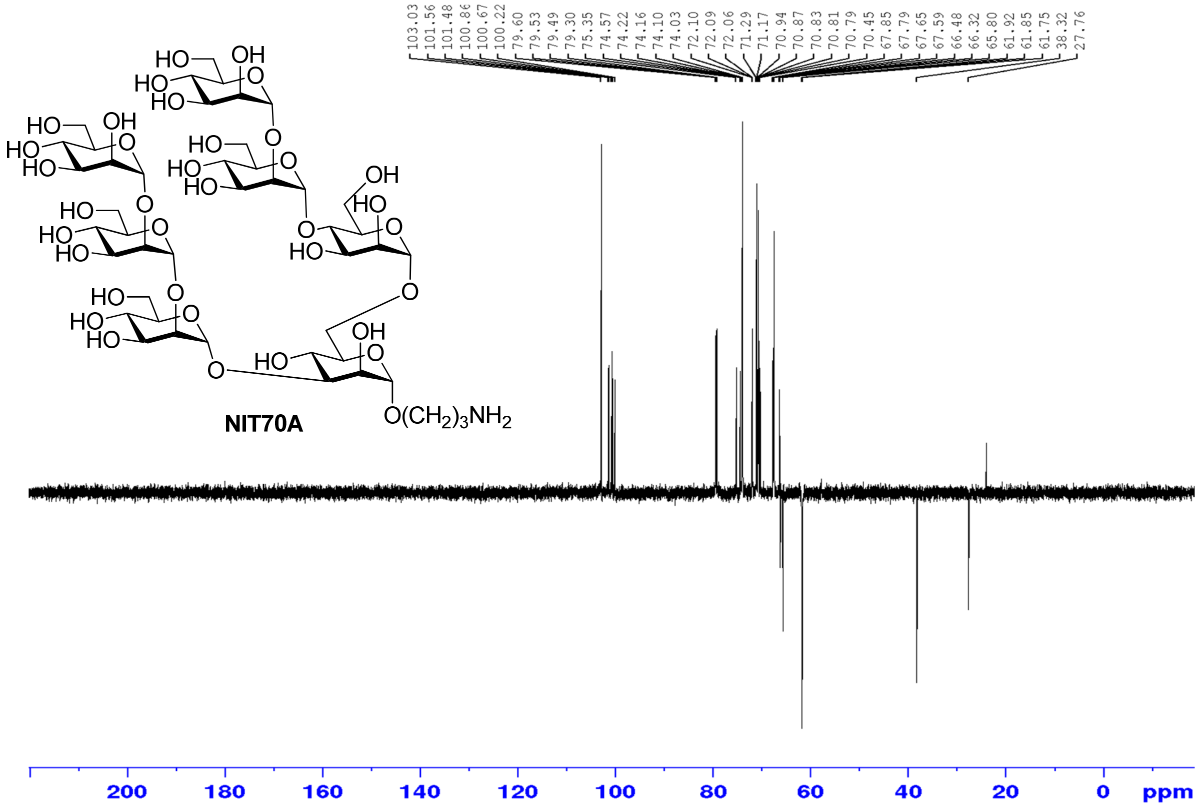

Supplementary Figure 20 | ^{1}H NMR spectrum (top) and ^{13}C NMR spectrum (bottom) of **NIT70A**.

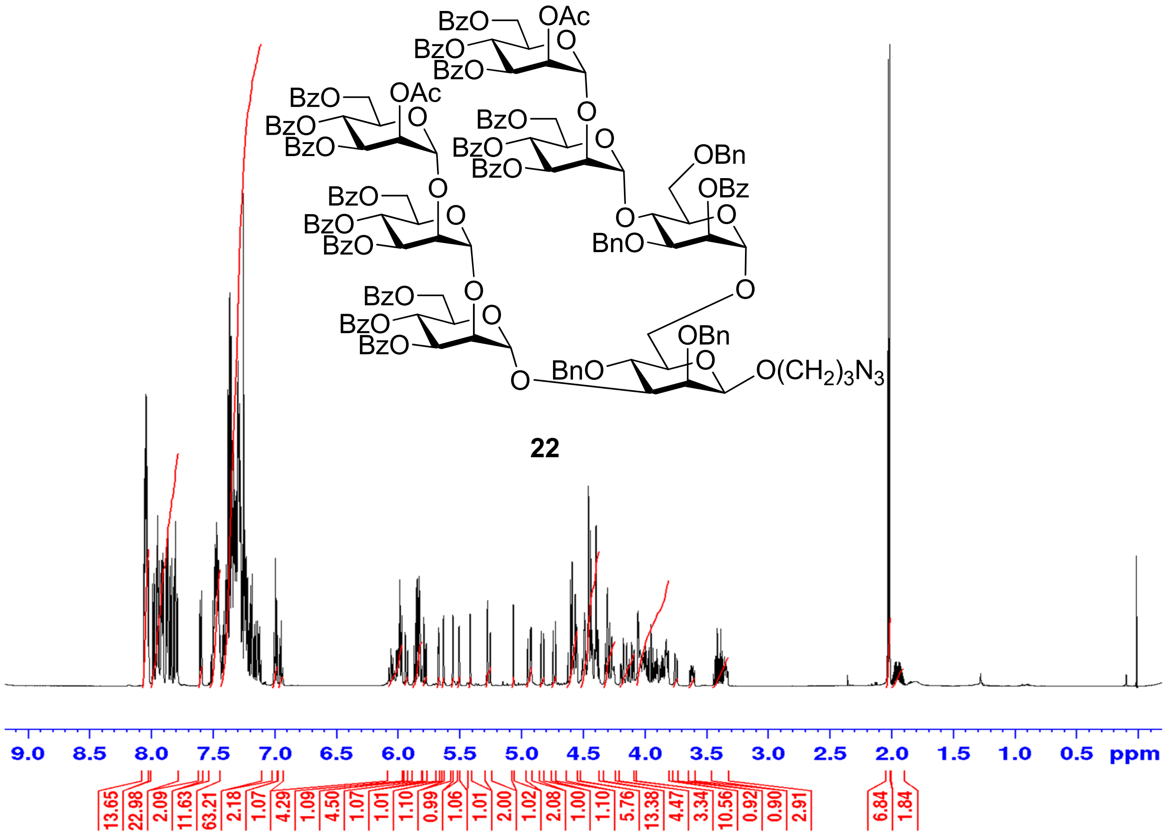

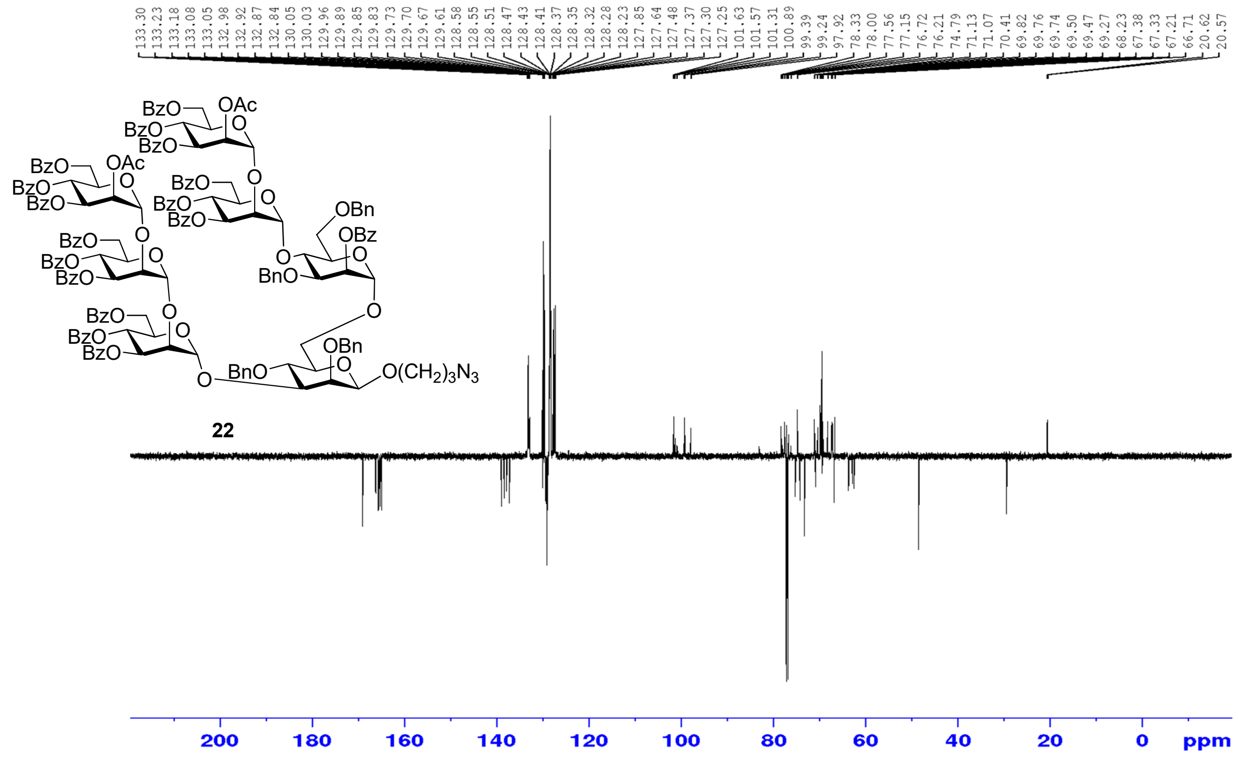

Supplementary Figure 21 | ^{1}H NMR spectrum (top) and ^{13}C NMR spectrum (bottom) of **22**.

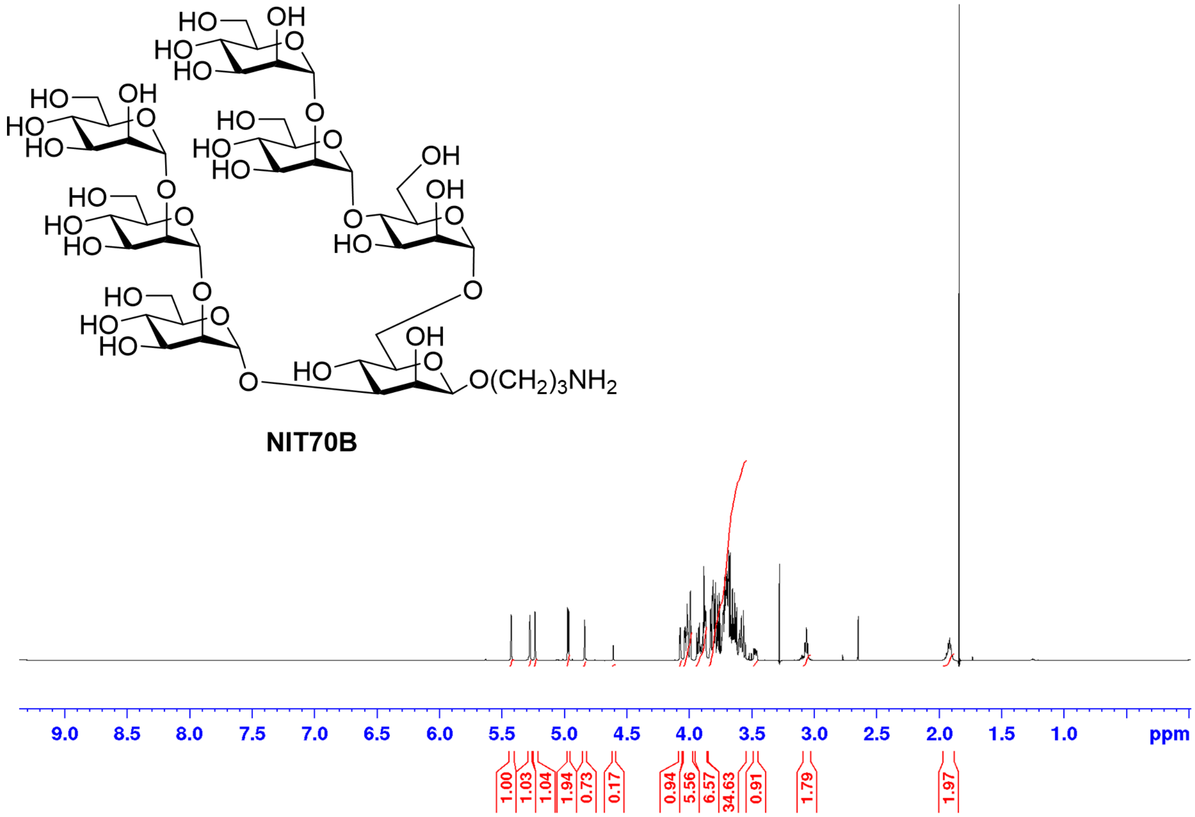

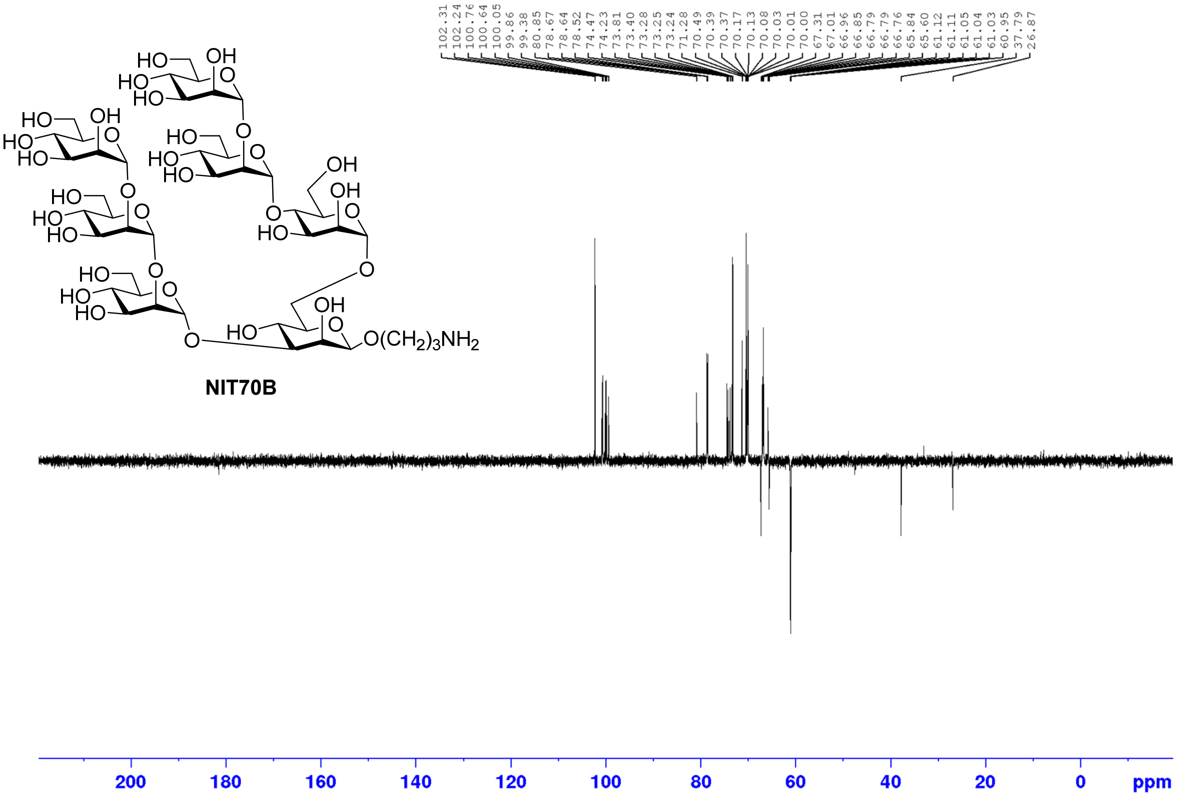

Supplementary Figure 22 | ^{1}H NMR spectrum (top) and ^{13}C NMR spectrum (bottom) of **NIT70B**.

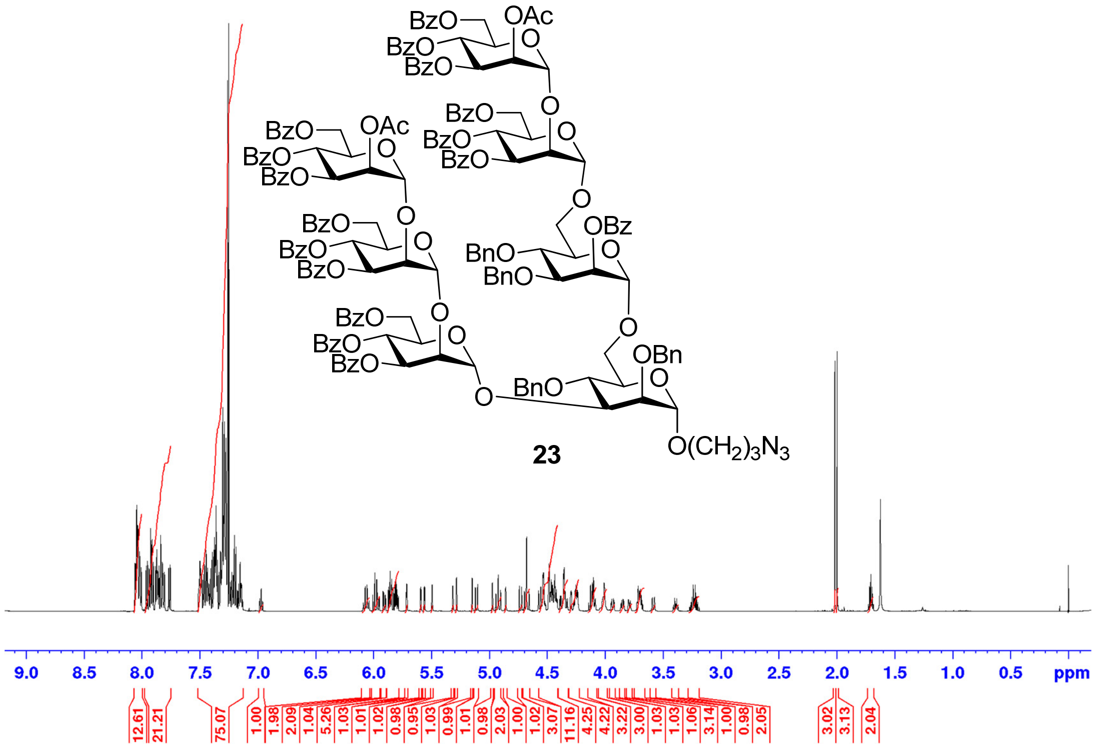

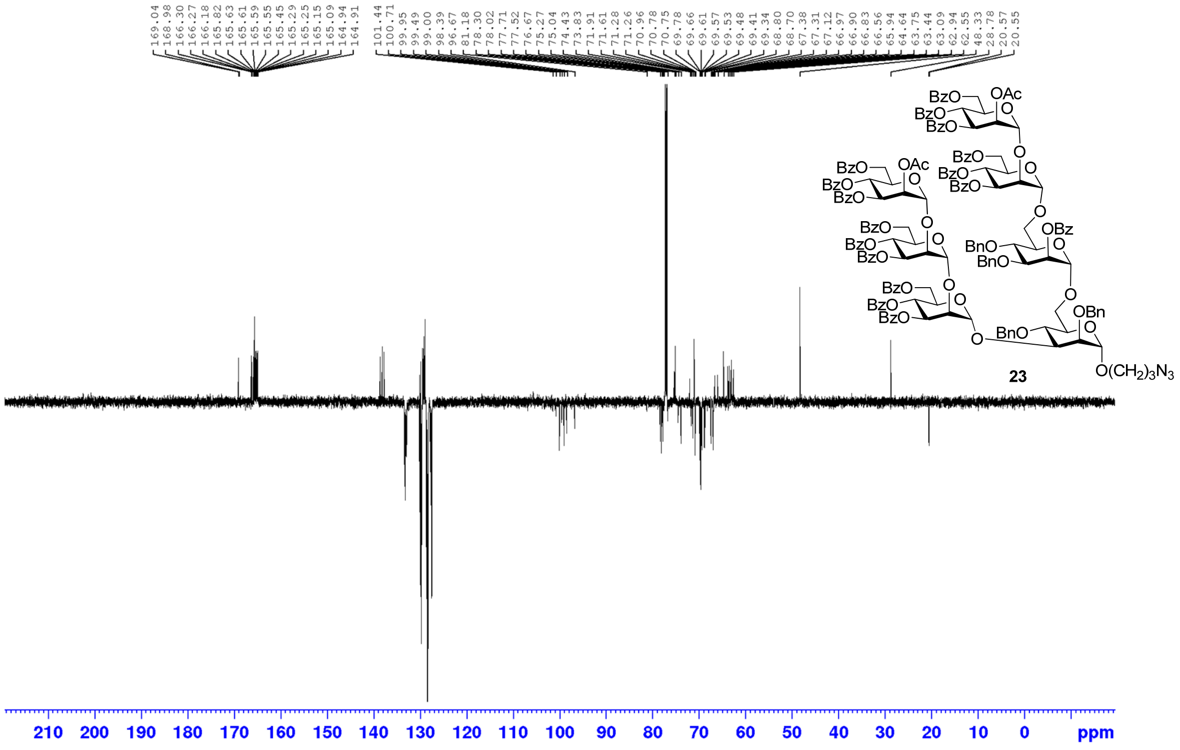

Supplementary Figure 23 | ^{1}H NMR spectrum (top) and ^{13}C NMR spectrum (bottom) of **23**.

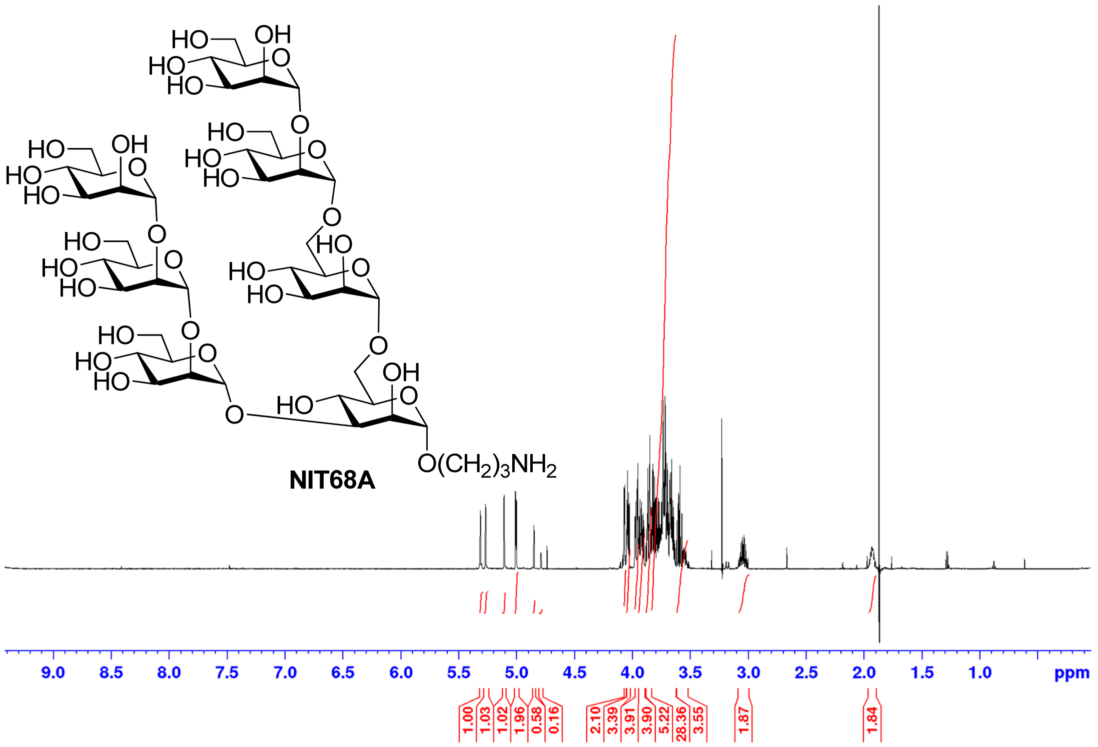

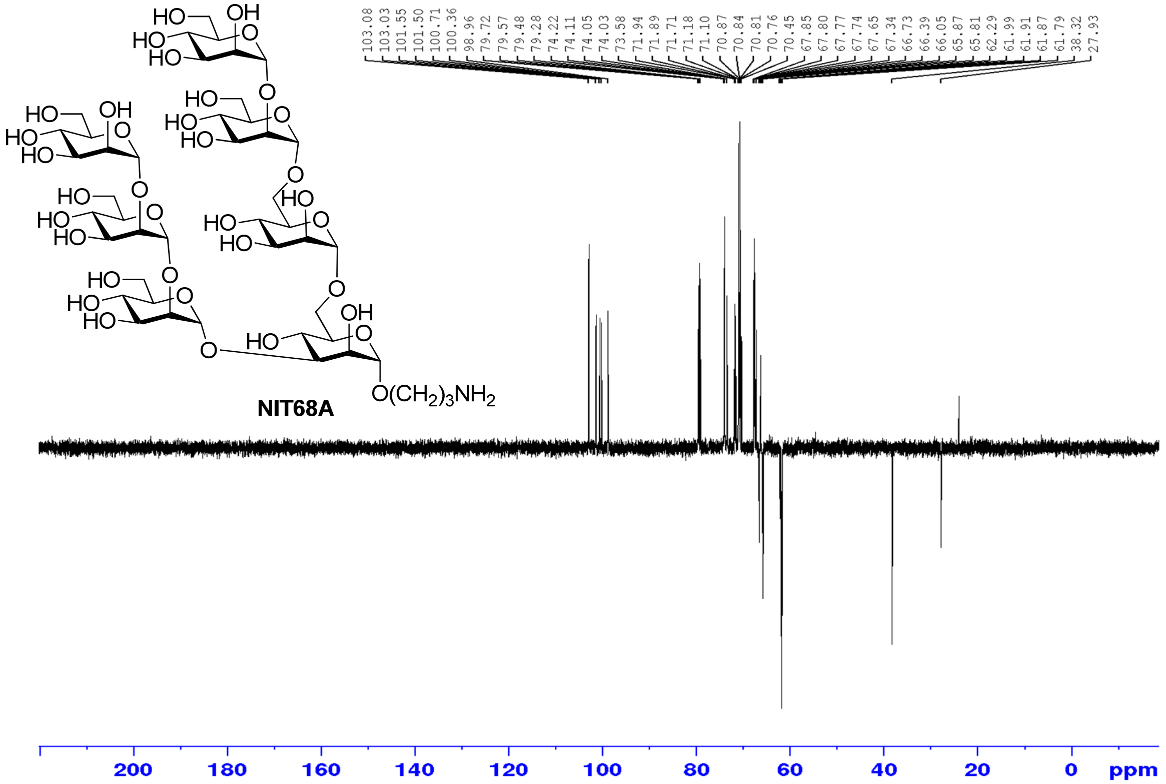

Supplementary Figure 24 | ^{1}H NMR spectrum (top) and ^{13}C NMR spectrum (bottom) of **NIT68A**.

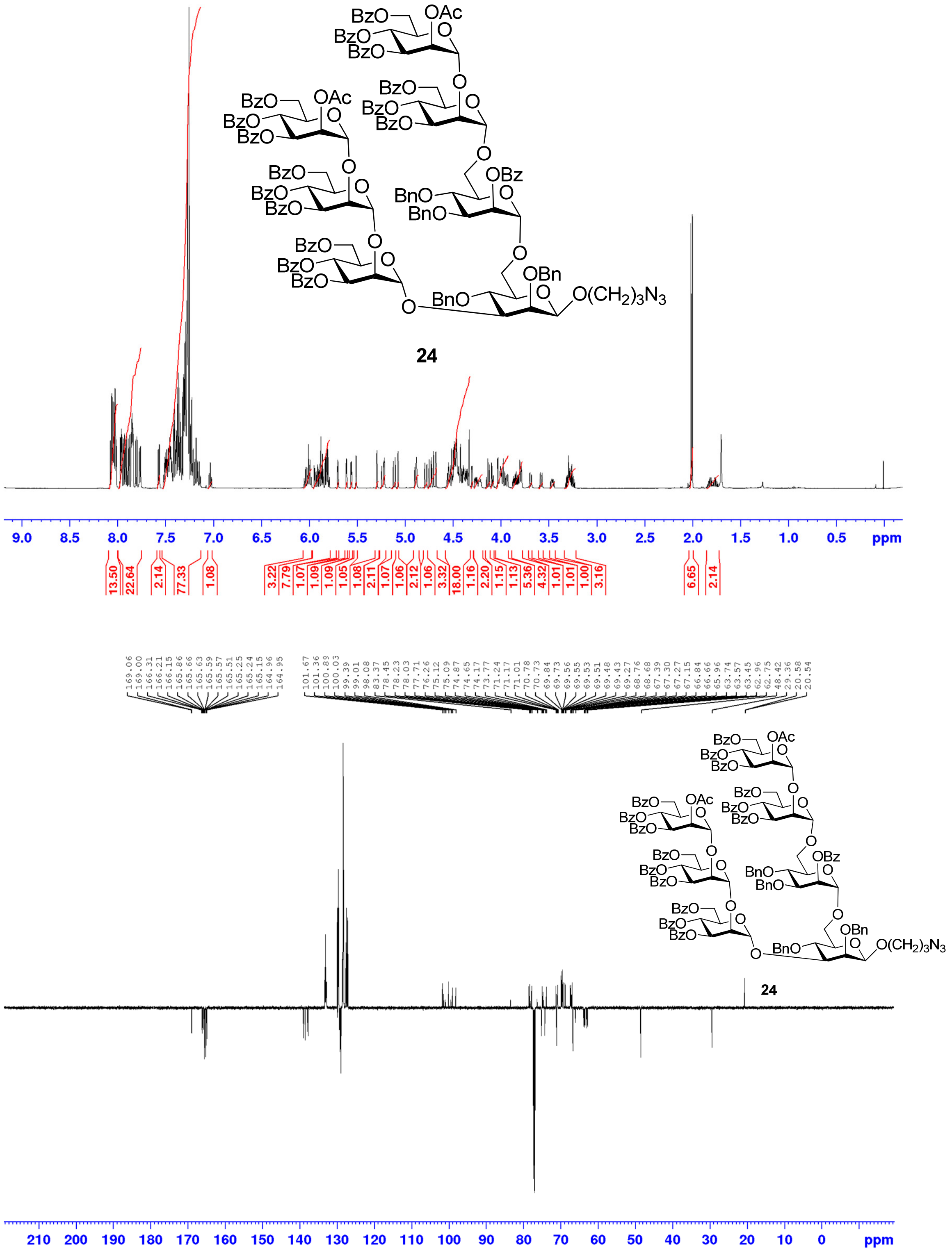

Supplementary Figure 25 | ^{1}H NMR spectrum (top) and ^{13}C NMR spectrum (bottom) of **24**.

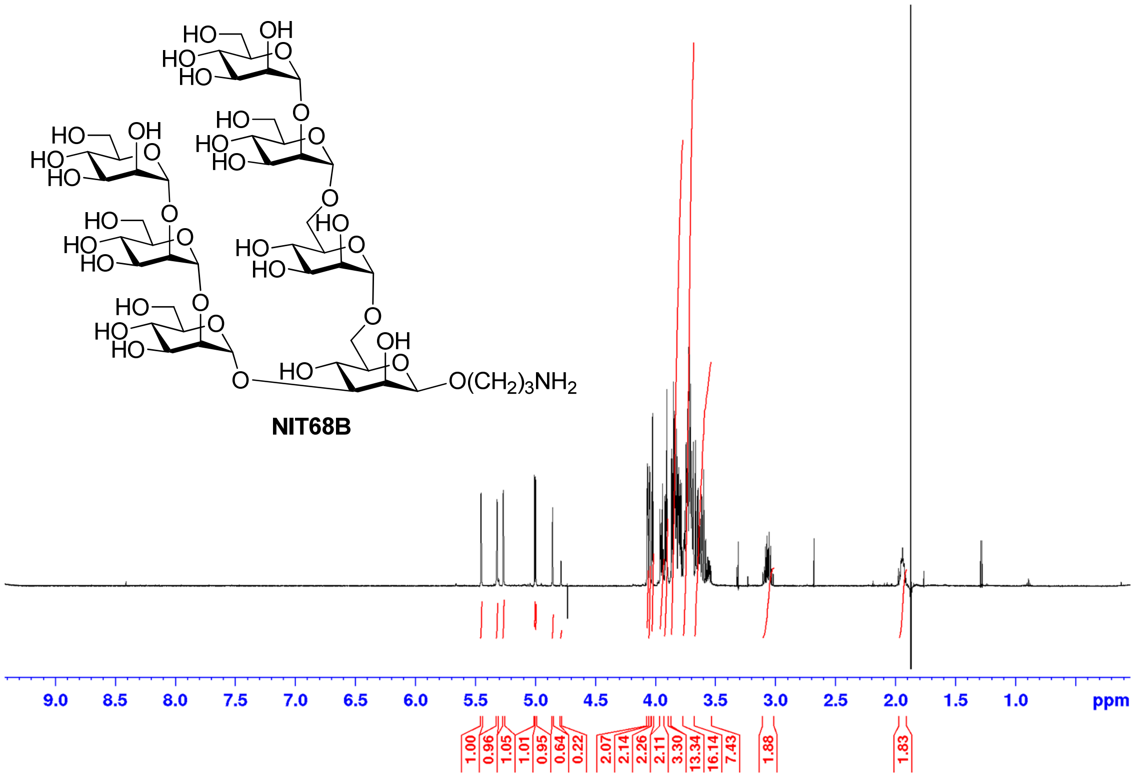

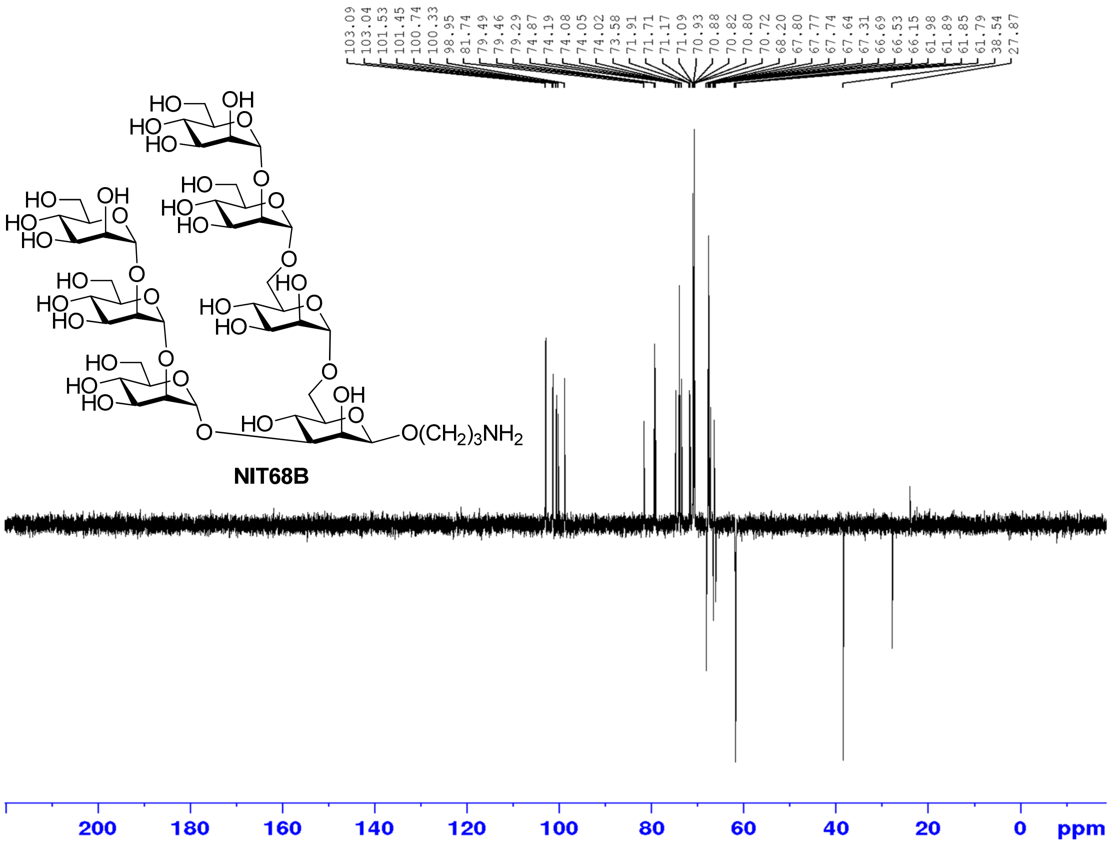

Supplementary Figure 26 | ^{1}H NMR spectrum (top) and ^{13}C NMR spectrum (bottom) of **NIT68B**.

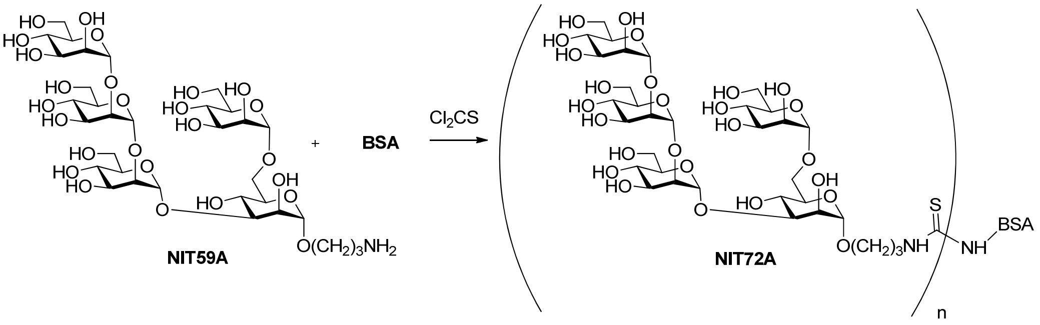

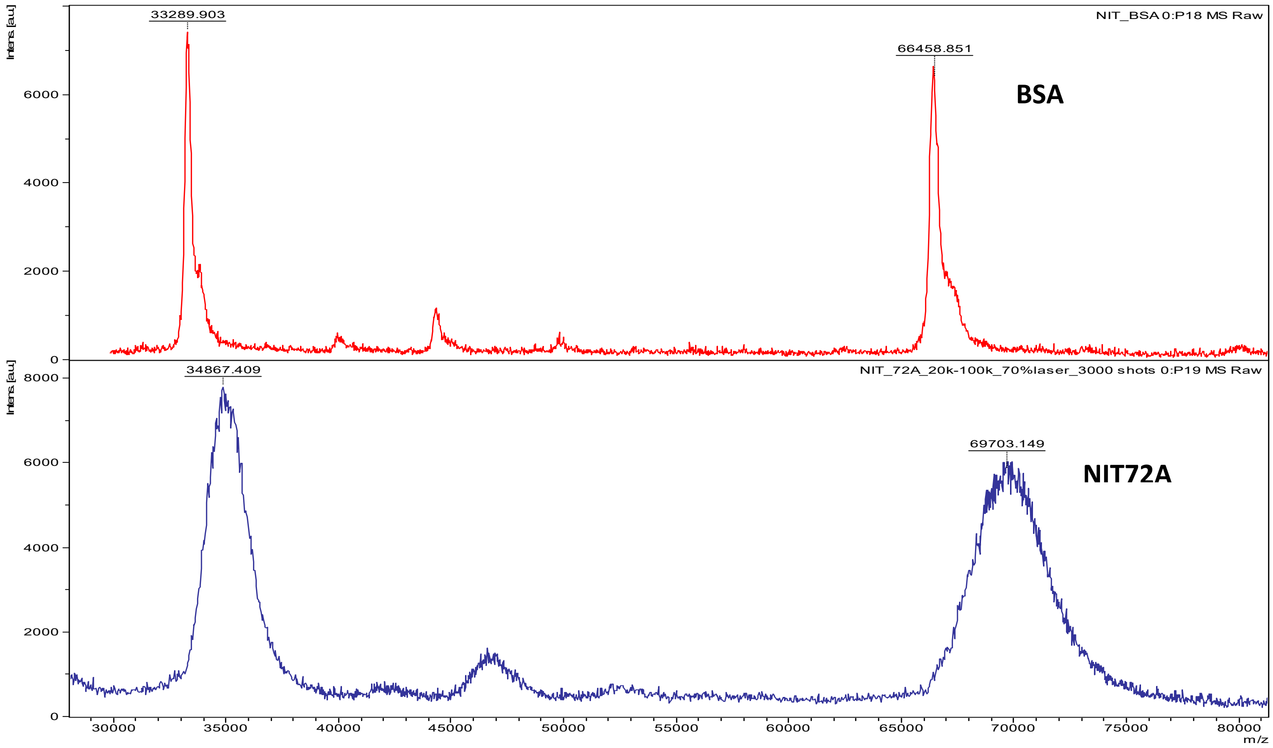

Supplementary Figure 27 | MALDI-TOF spectrum of NIT72A. *Top:* Conjugate NIT72A was synthesized as per general procedure B using NIT59A (2.0 mg; 2 μmol), to give 2.1 mg of product. *Bottom:* MALDI-TOF spectrum of NIT72A (bottom) and BSA (top). The mass spectroscopic analysis revealed a ligand:BSA ratio of 3.5:1.

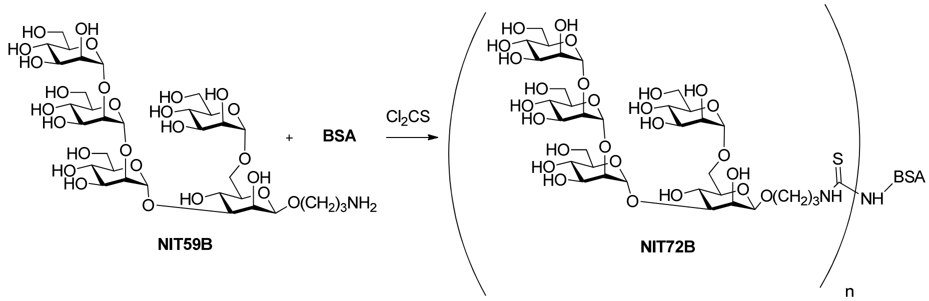

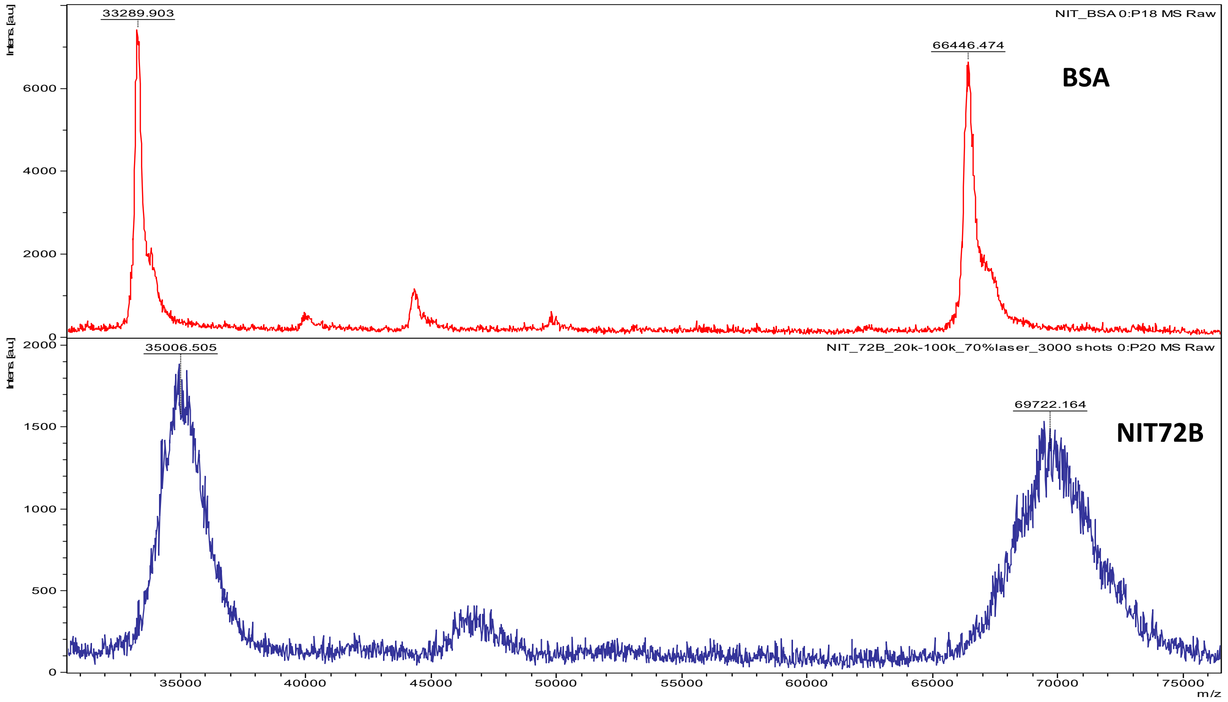

Supplementary Figure 28 | MALDI-TOF spectrum of NIT72B. *Top:* Conjugate NIT72B was synthesized as per general procedure B using NIT59B (2 mg; 2 μmol) to give 2.1 mg of product. *Bottom:* MALDI-TOF spectrum of NIT72B (bottom) and BSA (top). The mass spectroscopic analysis revealed a ligand:BSA ratio of 3.3:1.

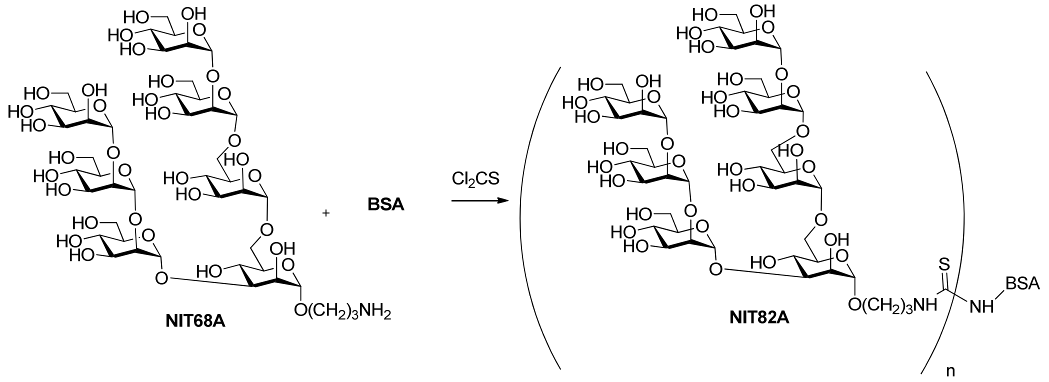

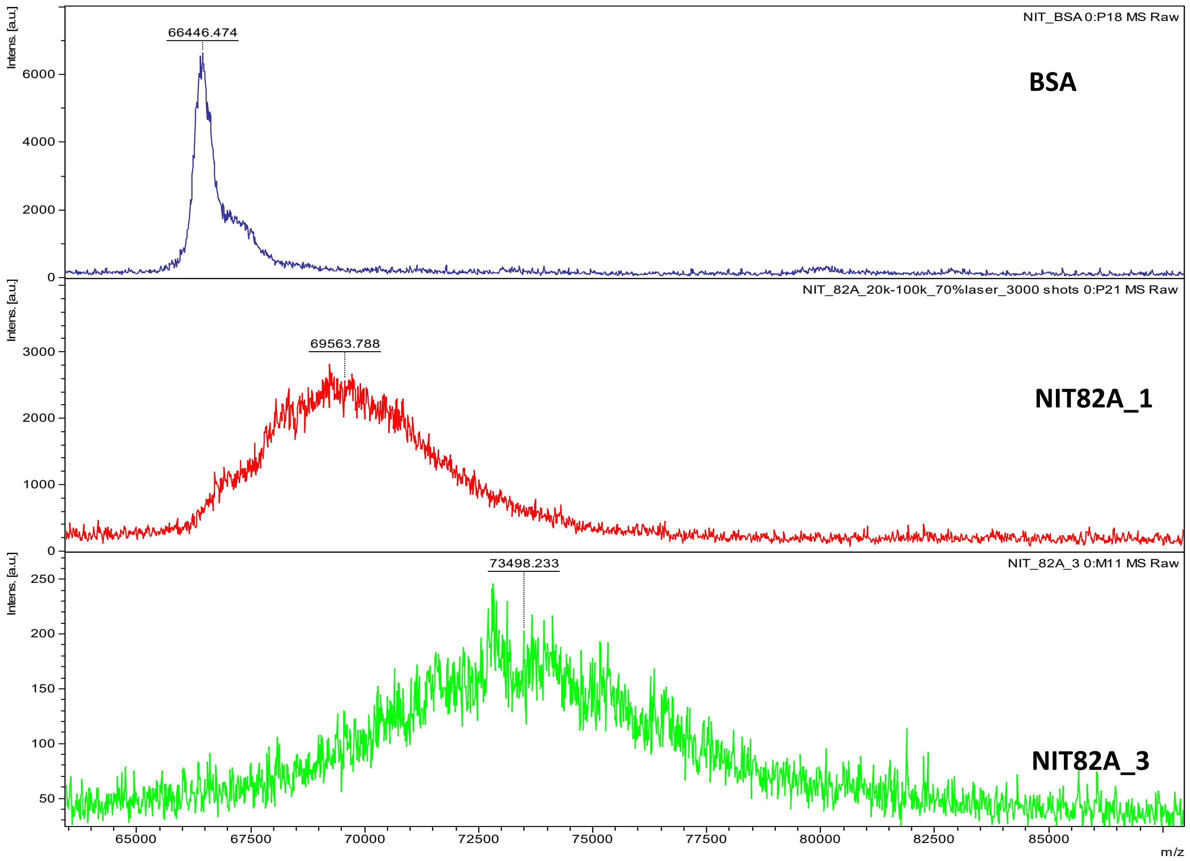

Supplementary Figure 29 | MALDI-TOF spectrum of NIT82A derivatives. *Top:* NIT82A_1 was synthesized as per general procedure B using NIT68A (2.7 mg; 2 μmol) to furnish 2.8 mg of BSA-conjugate. NIT82A_3 was synthesized per a slightly modified version of general procedure B using NIT68A (3.5 mg; 3 μmol) in 0.1 M aqu. $NaHCO_3$ (2 ml). After conjugation to the linker and removal of residual thiophosgen, it was added to a solution of BSA (1 mg) in buffer A to furnish 1 mg of BSA-conjugate. *Bottom:* MALDI-TOF spectrum of NIT82A_3 (bottom), NIT82A_1 (middle) and BSA (top). The MALDI-TOF mass spectroscopic analysis revealed a ligand:BSA ratio of 2.3:1 (NIT82A_1) and 5.4:1 (NIT82A_3).

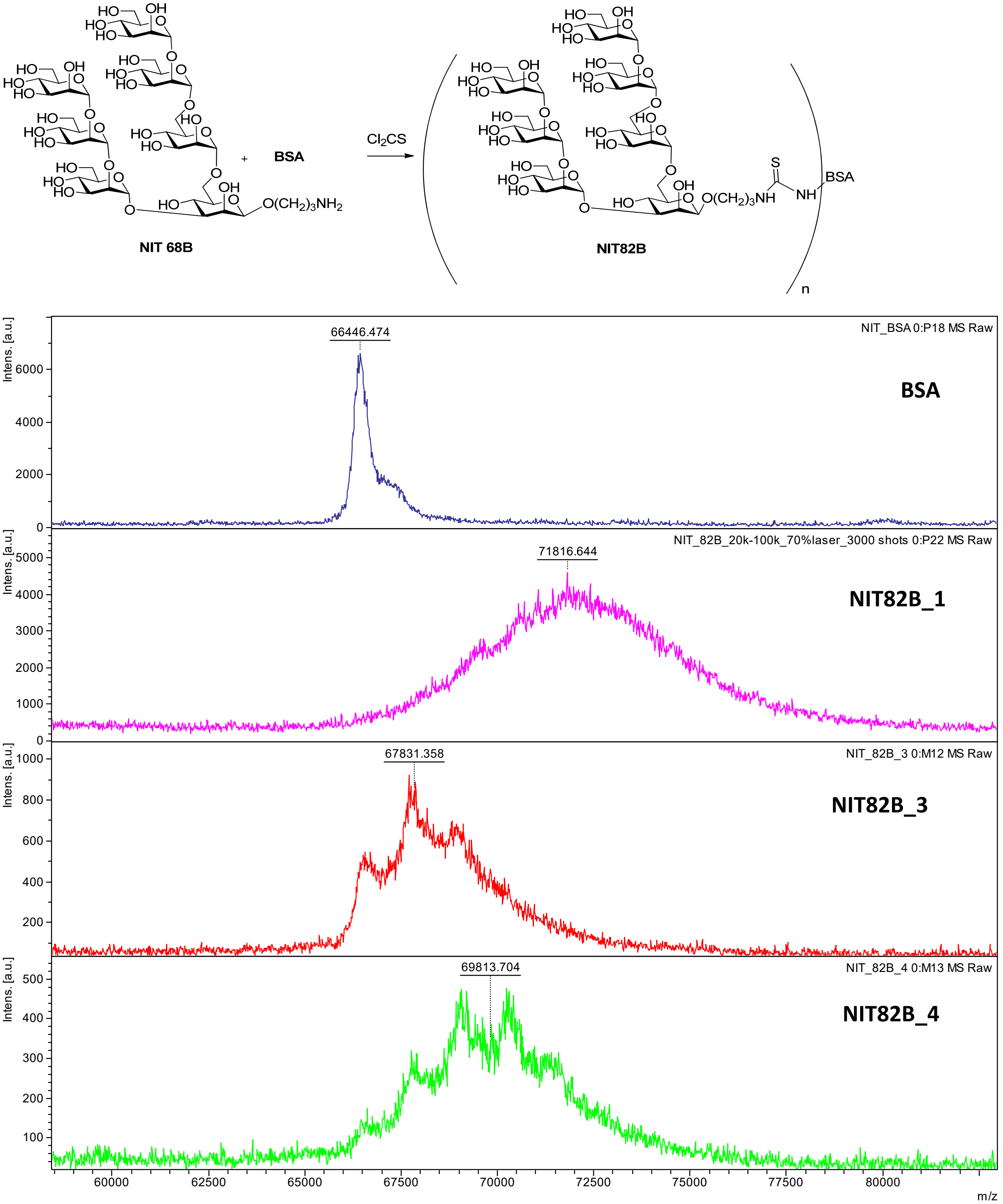

Supplementary Figure 30 | MALDI-TOF spectrum of NIT82B derivatives. *Top:* Compound NIT82B_1 was synthesized per general procedure B using (2.7 mg; 2 μmol) of NIT68B to give 2.9 mg of BSA-conjugate. NIT82B_3 was synthesized according to a slightly modified version of general procedure B using NIT68B (0.5 mg; 0.4 μmol) in 0.1 M aqu. $NaHCO_3$ (1 ml) and

thiophosgen (0.5 ml of 6 mM in $CHCl_3$; 450 eq). After conjugation to the linker and removal of residual thiophosgen, it was added to a solution of BSA (1 mg) in buffer A (0.5 ml) to furnish 1 mg of BSA-conjugate. NIT82B_4 was synthesized according to a slightly modified version of general procedure B using NIT68B (1 mg; 0.8 μmol) in 0.1 M aqu. $NaHCO_3$ (1 ml) and thiophosgen (1 ml of 6 mM in $CHCl_3$; 450 eq). After conjugation to the linker and removal of residual thiophosgen, it was added to a solution of BSA (1 mg) in buffer A (0.5 ml) to furnish 1 mg of BSA-conjugate. *Bottom:* MALDI-TOF spectrum of BSA, NIT82B_1, NIT82B_3 and NIT82B_4 (top to bottom, respectively). The MALDI-TOF mass spectroscopic analysis revealed a ligand:BSA ratio of 4.4:1 (NIT82B_1), 1.3:1 (NIT82B_3) and 2.6:1 (NIT82B_4)

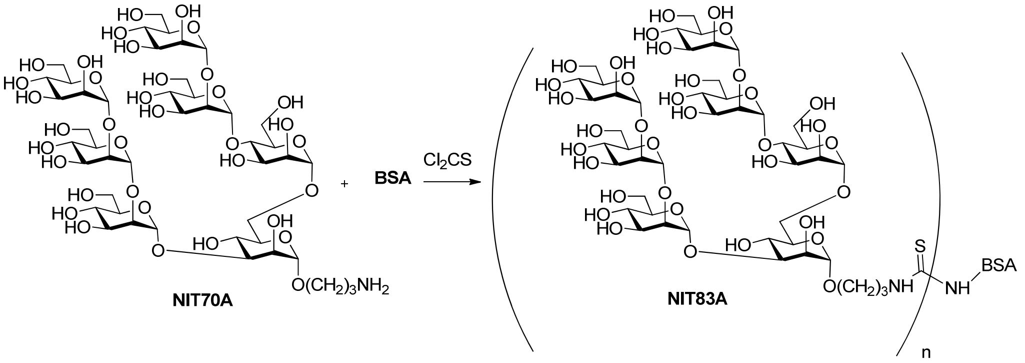

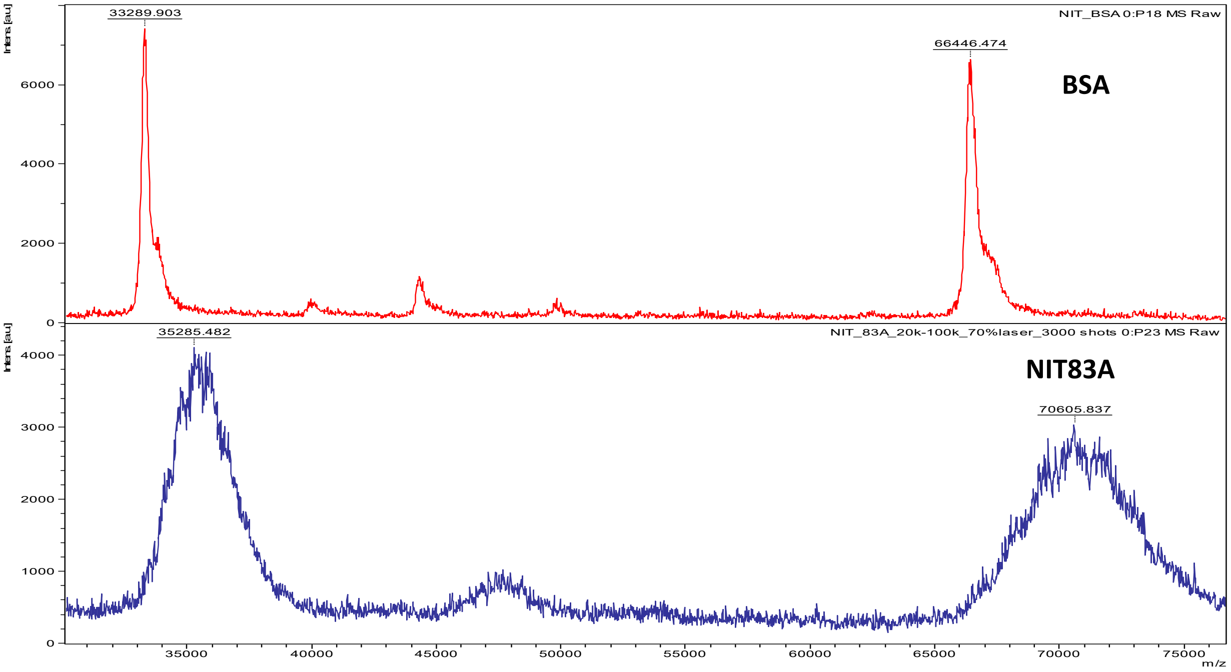

Supplementary Figure 31 | MALDI-TOF spectrum of NIT83A. *Top:* Conjugate NIT83A was synthesized as per general procedure B using NIT70A (2.7 mg; 2 µmol) to afford 2.8 mg of BSA-conjugate. *Bottom:* MALDI-TOF spectrum of NIT83A (bottom) and BSA (top). MALDI-TOF mass spectroscopic analysis revealed a ligand:BSA ratio of 3.4:1.

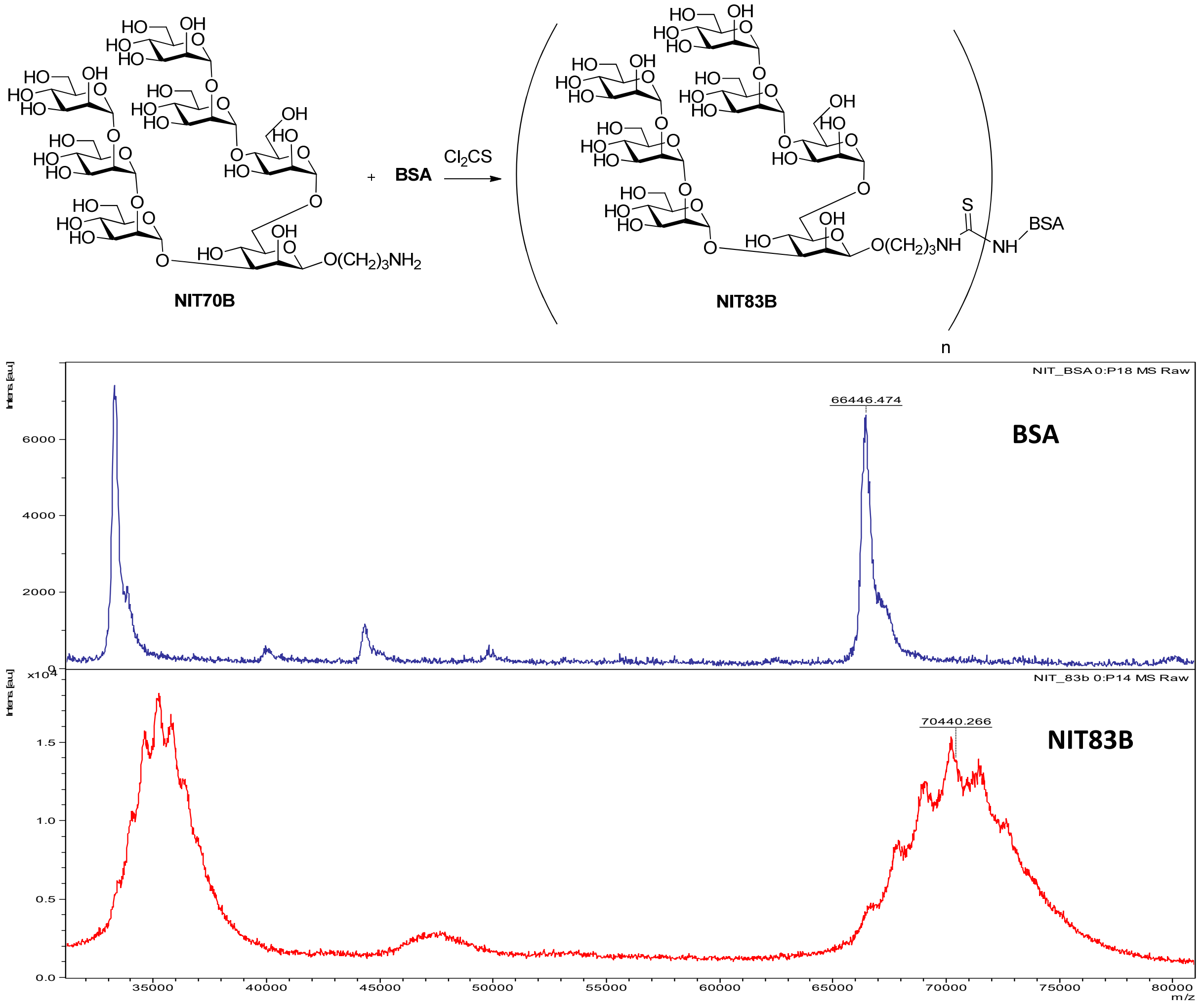

Supplementary Figure 32 | MALDI-TOF spectrum of NIT83B. *Top:* Conjugate NIT83B was synthesized per general procedure B using NIT70B (2.7 mg; 2 μmol) to afford 2.8 mg of BSA-conjugate. *Bottom:* MALDI-TOF spectrum of NIT83B (bottom) and BSA (top). MALDI-TOF mass spectroscopic analysis revealed a ligand:BSA ratio of 3.1:1.

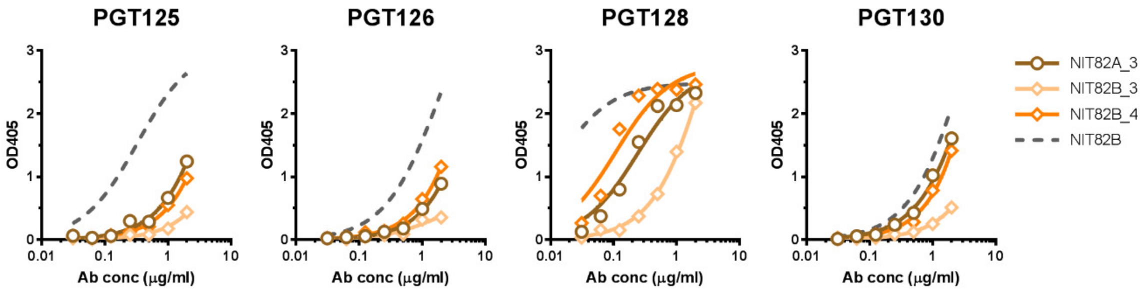

Supplementary Figure 33 | Glycoside titration reveals importance of loading density on glycoconjugates NIT82A and NIT82B for antibody binding. NAbs PGT125, 126, 128 and 130 were assayed for binding to a NIT82A conjugate with an average of 5.4 glycosides per mol BSA (NIT82A_3) and two NIT82B conjugates with average glycoside densities of 1.5 and 2.6 per BSA (NIT82B_3 and NIT82B_4) (**Supplementary Figs 29 and 30**). Conjugate NIT82B, with an average of 4.4 glycosides per BSA, was included as a comparator. The conjugates were coated as solid phase antigen onto ELISA plate wells (5 μg ml^{-1}). All antibodies were tested as IgGs. The antibodies bound stronger to conjugate NIT82A_3, assayed here with 5.4 glycosides per BSA, compared to its counterpart loaded at half the density (**Fig. 3a**). Consistent with these findings, the antibodies bound progressively better to NIT82B conjugates with increasing ligand density (1.5→4.4 per mol BSA). The results are from one experiment, performed in duplicate.

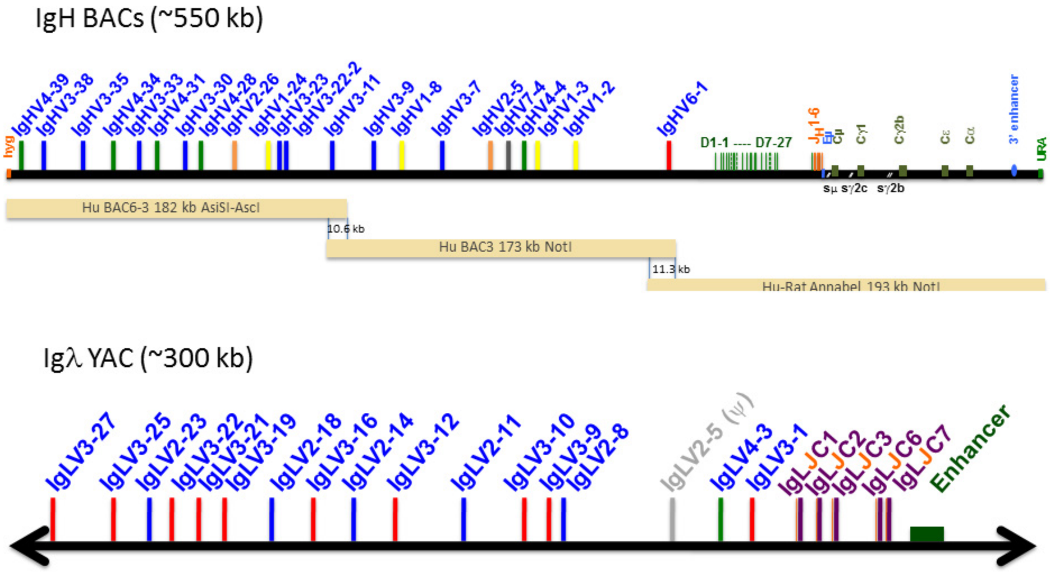

Supplementary Figure 34 | Integrated human Ig loci of OmniRat^{TM} strain[9,10]. *(Top)* The chimeric human-rat IgH region contains 3 overlapping BACs with 22 different and potentially functional human V_H segments. BAC6-3 has been extended with V_H3-11 to provide a 10.6 kb overlap to BAC3, which overlaps 11.3 kb via V_H6-1 with the C region BAC Hu-Rat Annabel. The latter is chimeric and contains all human D and J_H segments followed by the rat C region (Cμ, Cγ1, Cγ2b, Cε, Cα) with full enhancer sequences. *(Bottom)* The human Igλ region with 17 Vλs and all J-Cλs, including the 3' enhancer, is from a YAC[11].

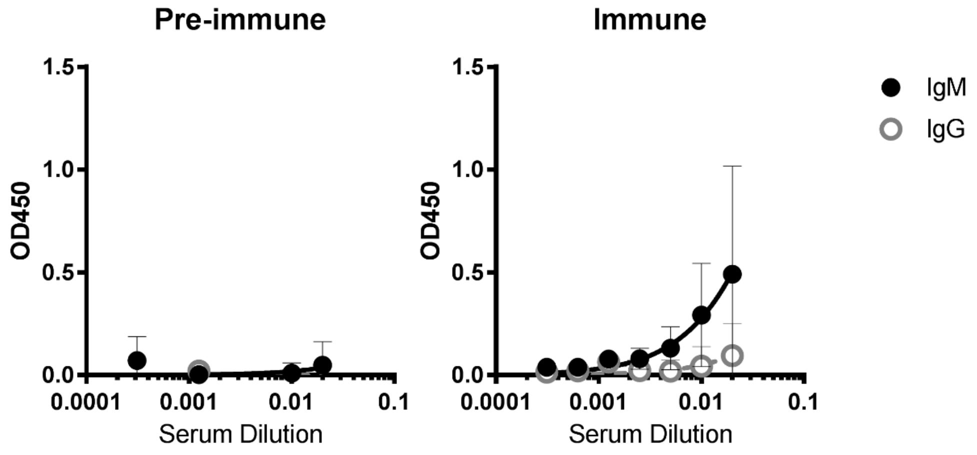

Supplementary Figure 35 | Antibody responses in NIT82B-immunized OmniRat animals are generally poor. Binding of IgM and IgG antibodies in pre-immune and immune sera to BSA (5 μg ml^{-1}) in

ELISA. The low levels of immune serum antibodies to BSA suggest that the immunogen formulation was poorly immunogenic in general. Shown are results from one experiment, performed in duplicate. Error bars denote the standard error from the mean.

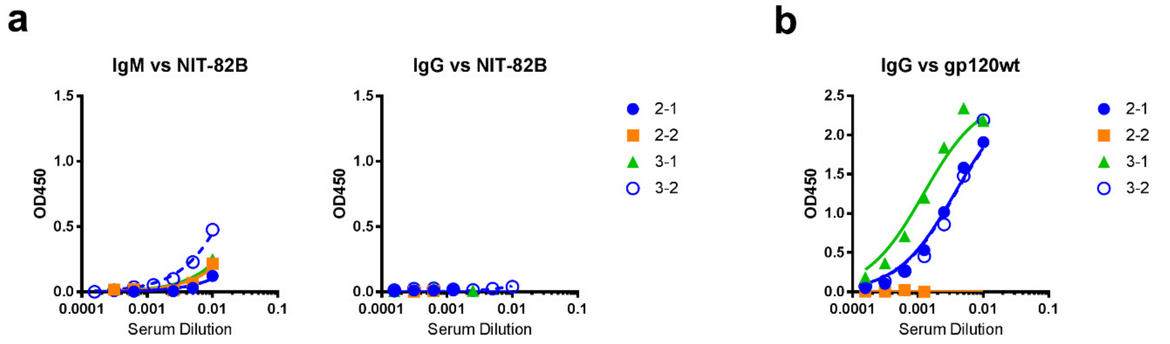

Supplementary Figure 36 | Immunization of OmniRat with recombinant gp120 does not readily elicit anti-glycan antibodies. Four transgenic rats were immunized 3x with recombinant gp120 expressed from stably transfected CHO-K1 cells. Sera collected 10 days after the final booster injection were assayed. (**a**) Assessment of serum IgM and IgG binding to the NIT82B glycoconjugate. The results show minimal IgM binding and no substantial IgG binding. (**b**) Serum IgG binding to recombinant gp120, showing that 3 of the 4 animals have high titers of gp120 binding antibodies. All results are from a single experiment, performed in duplicate.

JRFL wt	C	K	D	V	**N**	A	T	**N**	T	T	**N**	D	S	E	G	T	M	E	R	G	E	I	K	N
JRFL-T140A	C	K	D	V	**N**	A	T	N	T	**A**	**N**	D	S	E	G	T	M	E	R	G	E	I	K	N
JRFL-N141Q	C	K	D	V	**N**	A	T	**N**	T	T	**Q**	D	S	E	G	T	M	E	R	G	E	I	K	N
JRCSF wt	C	K	D	V	**N**	A	T	**N**	T	T	S	S	S	E	G	M	M	E	R	G	E	I	K	N

Supplementary Figure 37 | Comparison of JRFL and JRCSF V1 sequences. Wild-type JRFL (JRFL wt; top sequence) has three N-glycan attachment sites in V1 at positions 135, 138 and 141 (bold and underlined), whereas wild-type JRCSF has two, at positions 135 and 138 (JRCSF wt; bottom sequence). The two mutated JRFL sequences (JRFL-T140A, JRFL-N141Q) each eliminate an N-glycan site in JRFL: the Thr-to-Ala change at position 140 (T140A; red) eliminates the second glycan site while the Asn-to-Gln mutation at position 141 knocks out the third site (N141Q; red). Residue numbering for JRCSF and JRFL is based on the HXB2 reference sequence[12].

Supplementary Table 1. Chemical sequence and identification numbers of glycosides used for glycan array analyses

**1**	Galα-Sp8
**2**	Glcα-Sp8
**3**	Manα-Sp8
**4**	GalNAcα-Sp8
**5**	GalNAcα-Sp15
**6**	Fucα-Sp8
**7**	Fucα-Sp9
**8**	Rhaα-Sp8
**9**	Neu5Acα-Sp8
**10**	Neu5Acα-Sp11
**11**	Neu5Acβ-Sp8
**12**	Galβ-Sp8
**13**	Glcβ-Sp8
**14**	Manβ-Sp8
**15**	GalNAcβ-Sp8
**16**	GlcNAcβ-Sp0
**17**	GlcNAcβ-Sp8
**18**	GlcN(Gc)β-Sp8
**19**	Galβ1-4GlcNAcβ1-6(Galβ1-4GlcNAcβ1-3)GalNAcα-Sp8
**20**	Galβ1-4GlcNAcβ1-6(Galβ1-4GlcNAcβ1-3)GalNAc-Sp14

**21**	GlcNAcβ1-6(GlcNAcβ1-4)(GlcNAcβ1-3)GlcNAc-Sp8
**22**	6S(3S)Galβ1-4(6S)GlcNAcβ-Sp0
**23**	6S(3S)Galβ1-4GlcNAcβ-Sp0
**24**	(3S)Galβ1-4(Fucα1-3)(6S)Glc-Sp0
**25**	(3S)Galβ1-4Glcβ-Sp8
**26**	(3S)Galβ1-4(6S)Glcβ-Sp0
**27**	(3S)Galβ1-4(6S)Glcβ-Sp8
**28**	(3S)Galβ1-3(Fucα1-4)GlcNAcβ-Sp8
**29**	(3S)Galβ1-3GalNAcα-Sp8
**30**	(3S)Galβ1-3GlcNAcβ-Sp0
**31**	(3S)Galβ1-3GlcNAcβ-Sp8
**32**	(3S)Galβ1-4(Fucα1-3)GlcNAc-Sp0
**33**	(3S)Galβ1-4(Fucα1-3)GlcNAc-Sp8
**34**	(3S)Galβ1-4(6S)GlcNAcβ-Sp0
**35**	(3S)Galβ1-4(6S)GlcNAcβ-Sp8
**36**	(3S)Galβ1-4GlcNAcβ-Sp0
**37**	(3S)Galβ1-4GlcNAcβ-Sp8
**38**	(3S)Galβ-Sp8
**39**	(6S)(4S)Galβ1-4GlcNAcβ-Sp0
**40**	(4S)Galβ1-4GlcNAcβ-Sp8
**41**	(6P)Manα-Sp8
**42**	(6S)Galβ1-4Glcβ-Sp0

**43**	(6S)Galβ1-4Glcβ-Sp8
**44**	(6S)Galβ1-4GlcNAcβ-Sp8
**45**	(6S)Galβ1-4(6S)Glcβ-Sp8
**46**	Neu5Acα2-3(6S)Galβ1-4GlcNAcβ-Sp8
**47**	(6S)GlcNAcβ-Sp8
**48**	Neu5,9Ac$_2$α-Sp8
**49**	Neu5,9Ac2α2-6Galβ1-4GlcNAcβ-Sp8
**50**	Manα1-6(Manα1-3)Manβ1-4GlcNAcβ1-4GlcNAcβ-Sp12
**51**	Manα1-6(Manα1-3)Manβ1-4GlcNAcβ1-4GlcNAcβ-Sp13
**52**	GlcNAcβ1-2Manα1-6(GlcNAcβ1-2Manα1-3)Manβ1-4GlcNAcβ1-4GlcNAcβ-Sp12
**53**	GlcNAcβ1-2Manα1-6(GlcNAcβ1-2Manα1-3)Manβ1-4GlcNAcβ1-4GlcNAcβ-Sp13
**54**	Galβ1-4GlcNAcβ1-2Manα1-6(Galβ1-4GlcNAcβ1-2Manα1-3)Manβ1-4GlcNAcβ1-4GlcNAcβ-Sp12
**55**	Neu5Acα2-6Galβ1-4GlcNAcβ1-2Manα1-6(Neu5Acα2-6Galβ1-4GlcNAcβ1-2Manα1-3)Manβ1-4GlcNAcβ1-4GlcNAcβ-Sp12
**56**	Neu5Acα2-6Galβ1-4GlcNAcβ1-2Manα1-6(Neu5Acα2-6Galβ1-4GlcNAcβ1-2Man-a1-3)Manβ1-4GlcNAcβ1-4GlcNAcβ-Sp21
**57**	Neu5Acα2-6Galβ1-4GlcNAcβ1-2Manα1-6(Neu5Acα2-6Galβ1-4GlcNAcβ1-2Manα1-3)Manβ1-4GlcNAcβ1-4GlcNAcβ-Sp24
**58**	Fucα1-2Galβ1-3GalNAcβ1-3Galα-Sp9
**59**	Fucα1-2Galβ1-3GalNAcβ1-3Galα1-4Galβ1-4Glcβ-Sp9
**60**	Fucα1-2Galβ1-3(Fucα1-4)GlcNAcβ-Sp8

**61**	Fucα1-2Galβ1-3GalNAcα-Sp8
**62**	Fucα1-2Galβ1-3GalNAcα-Sp14
**63**	Fucα1-2Galβ1-3GalNAcβ1-4(Neu5Acα2-3)Galβ1-4Glcβ-Sp0
**64**	Fucα1-2Galβ1-3GalNAcβ1-4(Neu5Acα2-3)Galβ1-4Glcβ-Sp9
**65**	Fucα1-2Galβ1-3GlcNAcβ1-3Galβ1-4Glcβ-Sp8
**66**	Fucα1-2Galβ1-3GlcNAcβ1-3Galβ1-4Glcβ-Sp10
**67**	Fucα1-2Galβ1-3GlcNAcβ-Sp0
**68**	Fucα1-2Galβ1-3GlcNAcβ-Sp8
**69**	Fucα1-2Galβ1-4(Fucα1-3)GlcNAcβ1-3Galβ1-4(Fucα1-3)GlcNAcβ-Sp0
**70**	Fucα1-2Galβ1-4(Fucα1-3)GlcNAcβ1-3Galβ1-4(Fucα1-3)GlcNAcβ1-3Galβ1-4(Fucα1-3)GlcNAcβ-Sp0
**71**	Fucα1-2Galβ1-4(Fucα1-3)GlcNAcβ-Sp0
**72**	Fucα1-2Galβ1-4(Fucα1-3)GlcNAcβ-Sp8
**73**	Fucα1-2Galβ1-4GlcNAcβ1-3Galβ1-4GlcNAcβ-Sp0
**74**	Fucα1-2Galβ1-4GlcNAcβ1-3Galβ1-4GlcNAcβ1-3Galβ1-4GlcNAcβ-Sp0
**75**	Fucα1-2Galβ1-4GlcNAcβ-Sp0
**76**	Fucα1-2Galβ1-4GlcNAcβ-Sp8
**77**	Fucα1-2Galβ1-4Glcβ-Sp0
**78**	Fucα1-2Galβ-Sp8
**79**	Fucα1-3GlcNAcβ-Sp8
**80**	Fucα1-4GlcNAcβ-Sp8
**81**	Fucβ1-3GlcNAcβ-Sp8

**82**	GalNAcα1-3(Fucα1-2)Galβ1-3GlcNAcβ-Sp0
**83**	GalNAcα1-3(Fucα1-2)Galβ1-4(Fucα1-3)GlcNAcβ-Sp0
**84**	(3S)Galβ1-4(Fucα1-3)Glcβ-Sp0
**85**	GalNAcα1-3(Fucα1-2)Galβ1-4GlcNAcβ-Sp0
**86**	GalNAcα1-3(Fucα1-2)Galβ1-4GlcNAcβ-Sp8
**87**	GalNAcα1-3(Fucα1-2)Galβ1-4Glcβ-Sp0
**88**	GlcNAcβ1-3Galβ1-3GalNAcα-Sp8
**89**	GalNAcα1-3(Fucα1-2)Galβ-Sp8
**90**	GalNAcα1-3(Fucα1-2)Galβ-Sp18
**91**	GalNAcα1-3GalNAcβ-Sp8
**92**	GalNAcα1-3Galβ-Sp8
**93**	GalNAcα1-4(Fucα1-2)Galβ1-4GlcNAcβ-Sp8
**94**	GalNAcβ1-3GalNAcα-Sp8
**95**	GalNAcβ1-3(Fucα1-2)Galβ-Sp8
**96**	GalNAcβ1-3Galα1-4Galβ1-4GlcNAcβ-Sp0
**97**	GalNAcβ1-4(Fucα1-3)GlcNAcβ-Sp0
**98**	GalNAcβ1-4GlcNAcβ-Sp0
**99**	GalNAcβ1-4GlcNAcβ-Sp8
**100**	Galα1-2Galβ-Sp8
**101**	Galα1-3(Fucα1-2)Galβ1-3GlcNAcβ-Sp0
**102**	Galα1-3(Fucα1-2)Galβ1-3GlcNAcβ-Sp8
**103**	Galα1-3(Fucα1-2)Galβ1-4(Fucα1-3)GlcNAcβ-Sp0

**104**	Galα1-3(Fucα1-2)Galβ1-4(Fucα1-3)GlcNAcβ-Sp8
**105**	Galα1-3(Fucα1-2)Galβ1-4GlcNAc-Sp0
**106**	Galα1-3(Fucα1-2)Galβ1-4Glcβ-Sp0
**107**	Galα1-3(Fucα1-2)Galβ-Sp8
**108**	Galα1-3(Fucα1-2)Galβ-Sp18
**109**	Galα1-4(Galα1-3)Galβ1-4GlcNAcβ-Sp8
**110**	Galα1-3GalNAcα-Sp8
**111**	Galα1-3GalNAcα-Sp16
**112**	Galα1-3GalNAcβ-Sp8
**113**	Galα1-3Galβ1-4(Fucα1-3)GlcNAcβ-Sp8
**114**	Galα1-3Galβ1-3GlcNAcβ-Sp0
**115**	Galα1-3Galβ1-4GlcNAcβ-Sp8
**116**	Galα1-3Galβ1-4Glcβ-Sp0
**117**	Galα1-3Galβ1-4Glc-Sp10
**118**	Galα1-3Galβ-Sp8
**119**	Galα1-4(Fucα1-2)Galβ1-4GlcNAcβ-Sp8
**120**	Galα1-4Galβ1-4GlcNAcβ-Sp0
**121**	Galα1-4Galβ1-4GlcNAcβ-Sp8
**122**	Galα1-4Galβ1-4Glcβ-Sp0
**123**	Galα1-4GlcNAcβ-Sp8
**124**	Galα1-6Glcβ-Sp8
**125**	Galβ1-2Galβ-Sp8

**126**	Galβ1-3(Fucα1-4)GlcNAcβ1-3Galβ1-4(Fucα1-3)GlcNAcβ-Sp0
**127**	Galβ1-3GlcNAcβ1-3Galβ1-4(Fucα1-3)GlcNAcβ-Sp0
**128**	Galβ1-3(Fucα1-4)GlcNAc-Sp0
**129**	Galβ1-3(Fucα1-4)GlcNAc-Sp8
**130**	Fucα1-4(Galβ1-3)GlcNAcβ-Sp8
**131**	Galβ1-4GlcNAcβ1-6GalNAcα-Sp8
**132**	Galβ1-4GlcNAcβ1-6GalNAc-Sp14
**133**	GlcNAcβ1-6(Galβ1-3)GalNAcα-Sp8
**134**	GlcNAcβ1-6(Galβ1-3)GalNAcα-Sp14
**135**	Neu5Acα2-6(Galβ1-3)GalNAcα-Sp8
**136**	Neu5Acα2-6(Galβ1-3)GalNAcα-Sp14
**137**	Neu5Acβ2-6(Galβ1-3)GalNAcα-Sp8
**138**	Neu5Acα2-6(Galβ1-3)GlcNAcβ1-4Galβ1-4Glcβ-Sp10
**139**	Galβ1-3GalNAcα-Sp8
**140**	Galβ1-3GalNAcα-Sp14
**141**	Galβ1-3GalNAcα-Sp16
**142**	Galβ1-3GalNAcβ-Sp8
**143**	Galβ1-3GalNAcβ1-3Galα1-4Galβ1-4Glcβ-Sp0
**144**	Galβ1-3GalNAcβ1-4(Neu5Acα2-3)Galβ1-4Glcβ-Sp0
**145**	Galβ1-3GalNAcβ1-4Galβ1-4Glcβ-Sp8
**146**	Galβ1-3Galβ-Sp8
**147**	Galβ1-3GlcNAcβ1-3Galβ1-4GlcNAcβ-Sp0

**148**	Galβ1-3GlcNAcβ1-3Galβ1-4Glcβ-Sp10
**149**	Galβ1-3GlcNAcβ-Sp0
**150**	Galβ1-3GlcNAcβ-Sp8
**151**	Galβ1-4(Fucα1-3)GlcNAcβ-Sp0
**152**	Galβ1-4(Fucα1-3)GlcNAcβ-Sp8
**153**	Galβ1-4(Fucα1-3)GlcNAcβ1-3Galβ1-4(Fucα1-3)GlcNAcβ-Sp0
**154**	Galβ1-4(Fucα1-3)GlcNAcβ1-3Galβ1-4(Fucα1-3)GlcNAcβ1-3Galβ1-4(Fucα1-3)GlcNAcβ-Sp0
**155**	Galβ1-4(6S)Glcβ-Sp0
**156**	Galβ1-4(6S)Glcβ-Sp8
**157**	Galβ1-4GalNAcα1-3(Fucα1-2)Galβ1-4GlcNAcβ-Sp8
**158**	Galβ1-4GalNAcβ1-3(Fucα1-2)Galβ1-4GlcNAcβ-Sp8
**159**	Galβ1-4GlcNAcβ1-3GalNAcα-Sp8
**160**	Galβ1-4GlcNAcβ1-3GalNAc-Sp14
**161**	Galβ1-4GlcNAcβ1-3Galβ1-4(Fucα1-3)GlcNAcβ1-3Galβ1-4(Fucα1-3)GlcNAcβ-Sp0
**162**	Galβ1-4GlcNAcβ1-3Galβ1-4GlcNAcβ1-3Galβ1-4GlcNAcβ-Sp0
**163**	Galβ1-4GlcNAcβ1-3Galβ1-4GlcNAcβ-Sp0
**164**	Galβ1-4GlcNAcβ1-3Galβ1-4Glcβ-Sp0
**165**	Galβ1-4GlcNAcβ1-3Galβ1-4Glcβ-Sp8
**166**	Galβ1-4GlcNAcβ1-6(Galβ1-3)GalNAcα-Sp8
**167**	Galβ1-4GlcNAcβ1-6(Galβ1-3)GalNAc-Sp14
**168**	Galβ1-4GlcNAcβ-Sp0

**169**	Galβ1-4GlcNAcβ-Sp8
**170**	Galβ1-4GlcNAcβ-Sp23
**171**	Galβ1-4Glcβ-Sp0
**172**	Galβ1-4Glcβ-Sp8
**173**	GlcNAcα1-3Galβ1-4GlcNAcβ-Sp8
**174**	GlcNAcα1-6Galβ1-4GlcNAcβ-Sp8
**175**	GlcNAcβ1-2Galβ1-3GalNAcα-Sp8
**176**	GlcNAcβ1-6(GlcNAcβ1-3)GalNAcα-Sp8
**177**	GlcNAcβ1-6(GlcNAcβ1-3)GalNAcα-Sp14
**178**	GlcNAcβ1-6(GlcNAcβ1-3)Galβ1-4GlcNAcβ-Sp8
**179**	GlcNAcβ1-3GalNAcα-Sp8
**180**	GlcNAcβ1-3GalNAcα-Sp14
**181**	GlcNAcβ1-3Galβ-Sp8
**182**	GlcNAcβ1-3Galβ1-4GlcNAcβ-Sp0
**183**	GlcNAcβ1-3Galβ1-4GlcNAcβ-Sp8
**184**	GlcNAcβ1-3Galβ1-4GlcNAcβ1-3Galβ1-4GlcNAcβ-Sp0
**185**	GlcNAcβ1-3Galβ1-4Glcβ-Sp0
**186**	GlcNAcβ1-4-MDPLys
**187**	GlcNAcβ1-6(GlcNAcβ1-4)GalNAcα-Sp8
**188**	GlcNAcβ1-4Galβ1-4GlcNAcβ-Sp8
**189**	GlcNAcβ1-4GlcNAcβ1-4GlcNAcβ1-4GlcNAcβ1-4GlcNAcβ1-4GlcNAcβ1-Sp8

**190**	GlcNAcβ1-4GlcNAcβ1-4GlcNAcβ1-4GlcNAcβ1-4GlcNAcβ1-Sp8
**191**	GlcNAcβ1-4GlcNAcβ1-4GlcNAcβ-Sp8
**192**	GlcNAcβ1-6GalNAcα-Sp8
**193**	GlcNAcβ1-6GalNAcα-Sp14
**194**	GlcNAcβ1-6Galβ1-4GlcNAcβ-Sp8
**195**	Glcα1-4Glcβ-Sp8
**196**	Glcα1-4Glcα-Sp8
**197**	Glcα1-6Glcα1-6Glcβ-Sp8
**198**	Glcβ1-4Glcβ-Sp8
**199**	Glcβ1-6Glcβ-Sp8
**200**	G-ol-Sp8
**201**	GlcAα-Sp8
**202**	GlcAβ-Sp8
**203**	GlcAβ1-3Galβ-Sp8
**204**	GlcAβ1-6Galβ-Sp8
**205**	KDNα2-3Galβ1-3GlcNAcβ-Sp0
**206**	KDNα2-3Galβ1-4GlcNAcβ-Sp0
**207**	Manα1-2Manα1-2Manα1-3Manα-Sp9
**208**	Manα1-2Manα1-6(Manα1-2Manα1-3)Manα-Sp9
**209**	Manα1-2Manα1-3Manα-Sp9
**210**	Manα1-6(Manα1-2Manα1-3)Manα1-6(Manα1-2Manα1-3)Manβ1-4GlcNAcβ1-4GlcNAcβ-Sp12

**211**	Manα1-2Manα1-6(Manα1-3)Manα1-6(Manα1-2Manα1-2Manα1-3)Manβ1-4GlcNAcβ1-4GlcNAcβ-Sp12
**212**	Manα1-2Manα1-6(Manα1-2Manα1-3)Manα1-6(Manα1-2Manα1-2Manα1-3)Manβ1-4GlcNAcβ1-4GlcNAcβ-Sp12
**213**	Manα1-6(Manα1-3)Manα-Sp9
**214**	Manα1-2Manα1-2Manα1-6(Manα1-3)Manα-Sp9
**215**	Manα1-6(Manα1-3)Manα1-6(Manα1-2Manα1-3)Manβ1-4GlcNAcβ1-4GlcNAcβ-Sp12
**216**	Manα1-6(Manα1-3)Manα1-6(Manα1-3)Manβ1-4GlcNAcβ1-4GlcNAcβ-Sp12
**217**	Manβ1-4GlcNAcβ-Sp0
**218**	Neu5Acα2-3Galβ1-4GlcNAcβ1-3Galβ1-4(Fucα1-3)GlcNAcβ-Sp0
**219**	(3S)Galβ1-4(Fucα1-3)(6S)GlcNAcβ-Sp8
**220**	Fucα1-2(6S)Galβ1-4GlcNAcβ-Sp0
**221**	Fucα1-2Galβ1-4(6S)GlcNAcβ-Sp8
**222**	Fucα1-2(6S)Galβ1-4(6S)Glcβ-Sp0
**223**	Neu5Acα2-3Galβ1-3GalNAcα-Sp8
**224**	Neu5Acα2-3Galβ1-3GalNAcα-Sp14
**225**	GalNAcβ1-4(Neu5Acα2-8Neu5Acα2-8Neu5Acα2-8Neu5Acα2-3)Galβ1-4Glcβ-Sp0
**226**	GalNAcβ1-4(Neu5Acα2-8Neu5Acα2-8Neu5Acα2-3)Galβ1-4Glcβ-Sp0
**227**	Neu5Acα2-8Neu5Acα2-8Neu5Acα2-3Galβ1-4Glcβ-Sp0
**228**	GalNAcβ1-4(Neu5Acα2-8Neu5Acα2-3)Galβ1-4Glcβ-Sp0
**229**	Neu5Acα2-8Neu5Acα2-8Neu5Acα-Sp8

**230**	GalNAcβ1-4(Neu5Acα2-3)Galβ1-4GlcNAcβ-Sp0
**231**	GalNAcβ1-4(Neu5Acα2-3)Galβ1-4GlcNAcβ-Sp8
**232**	GalNAcβ1-4(Neu5Acα2-3)Galβ1-4Glcβ-Sp0
**233**	Neu5Acα2-3Galβ1-3GalNAcβ1-4(Neu5Acα2-3)Galβ1-4Glcβ-Sp0
**234**	Neu5Acα2-6(Neu5Acα2-3)GalNAcα-Sp8
**235**	Neu5Acα2-3GalNAcα-Sp8
**236**	Neu5Acα2-3GalNAcβ1-4GlcNAcβ-Sp0
**237**	Neu5Acα2-3Galβ1-3(6S)GlcNAc-Sp8
**238**	Neu5Acα2-3Galβ1-3(Fucα1-4)GlcNAcβ-Sp8
**239**	Neu5Acα2-3Galβ1-3(Fucα1-4)GlcNAcβ1-3Galβ1-4(Fucα1-3)GlcNAcβ-Sp0
**240**	Neu5Acα2-3Galβ1-4(Neu5Acα2-3Galβ1-3)GlcNAcβ-Sp8
**241**	Neu5Acα2-3Galβ1-3(6S)GalNAcα-Sp8
**242**	Neu5Acα2-6(Neu5Acα2-3Galβ1-3)GalNAcα-Sp8
**243**	Neu5Acα2-6(Neu5Acα2-3Galβ1-3)GalNAcα-Sp14
**244**	Neu5Acα2-3Galβ-Sp8
**245**	Neu5Acα2-3Galβ1-3GalNAcβ1-3Galα1-4Galβ1-4Glcβ-Sp0
**246**	Neu5Acα2-3Galβ1-3GlcNAcβ1-3Galβ1-4GlcNAcβ-Sp0
**247**	Fucα1-2(6S)Galβ1-4Glcβ-Sp0
**248**	Neu5Acα2-3Galβ1-3GlcNAcβ-Sp0
**249**	Neu5Acα2-3Galβ1-4(6S)GlcNAcβ-Sp8
**250**	Neu5Acα2-3Galβ1-4(Fucα1-3)(6S)GlcNAcβ-Sp8
**251**	Neu5Acα2-3Galβ1-4(Fucα1-3)GlcNAcβ1-3Galβ1-4(Fucα1-3)GlcNAcβ1-

	3Galβ1-4(Fucα1-3)GlcNAcβ-Sp0
**252**	Neu5Acα2-3Galβ1-4(Fucα1-3)GlcNAcβ-Sp0
**253**	Neu5Acα2-3Galβ1-4(Fucα1-3)GlcNAcβ-Sp8
**254**	Neu5Acα2-3Galβ1-4(Fucα1-3)GlcNAcβ1-3Galβ-Sp8
**255**	Neu5Acα2-3Galβ1-4(Fucα1-3)GlcNAcβ1-3Galβ1-4GlcNAcβ-Sp8
**256**	Neu5Acα2-3Galβ1-4GlcNAcβ1-3Galβ1-4GlcNAcβ1-3Galβ1-4GlcNAcβ-Sp0
**257**	Neu5Acα2-3Galβ1-4GlcNAcβ-Sp0
**258**	Neu5Acα2-3Galβ1-4GlcNAcβ-Sp8
**259**	Neu5Acα2-3Galβ1-4GlcNAcβ1-3Galβ1-4GlcNAcβ-Sp0
**260**	Fucα1-2Galβ1-4(6S)Glcβ-Sp0
**261**	Neu5Acα2-3Galβ1-4Glcβ-Sp0
**262**	Neu5Acα2-3Galβ1-4Glcβ-Sp8
**263**	Neu5Acα2-6GalNAcα-Sp8
**264**	Neu5Acα2-6GalNAcβ1-4GlcNAcβ-Sp0
**265**	Neu5Acα2-6Galβ1-4(6S)GlcNAcβ-Sp8
**266**	Neu5Acα2-6Galβ1-4GlcNAcβ-Sp0
**267**	Neu5Acα2-6Galβ1-4GlcNAcβ-Sp8
**268**	Neu5Acα2-6Galβ1-4GlcNAcβ1-3Galβ1-4(Fucα1-3)GlcNAcβ1-3Galβ1-4(Fucα1-3)GlcNAcβ-Sp0
**269**	Neu5Acα2-6Galβ1-4GlcNAcβ1-3Galβ1-4GlcNAcβ-Sp0
**270**	Neu5Acα2-6Galβ1-4Glcβ-Sp0
**271**	Neu5Acα2-6Galβ1-4Glcβ-Sp8

**272**	Neu5Acα2-6Galβ-Sp8
**273**	Neu5Acα2-8Neu5Acα-Sp8
**274**	Neu5Acα2-8Neu5Acα2-3Galβ1-4Glcβ-Sp0
**275**	Galβ1-3(Fucα1-4)GlcNAcβ1-3Galβ1-3(Fucα1-4)GlcNAcβ-Sp0
**276**	Neu5Acβ2-6GalNAcα-Sp8
**277**	Neu5Acβ2-6Galβ1-4GlcNAcβ-Sp8
**278**	Neu5Gcα2-3Galβ1-3(Fucα1-4)GlcNAcβ-Sp0
**279**	Neu5Gcα2-3Galβ1-3GlcNAcβ-Sp0
**280**	Neu5Gcα2-3Galβ1-4(Fucα1-3)GlcNAcβ-Sp0
**281**	Neu5Gcα2-3Galβ1-4GlcNAcβ-Sp0
**282**	Neu5Gcα2-3Galβ1-4Glcβ-Sp0
**283**	Neu5Gcα2-6GalNAcα-Sp0
**284**	Neu5Gcα2-6Galβ1-4GlcNAcβ-Sp0
**285**	Neu5Gcα-Sp8
**286**	Neu5Acα2-3Galβ1-4GlcNAcβ1-6(Galβ1-3)GalNAcα-Sp14
**287**	Galβ1-3GlcNAcβ1-3Galβ1-3GlcNAcβ-Sp0
**288**	Galβ1-4(Fucα1-3)(6S)GlcNAcβ-Sp0
**289**	Galβ1-4(Fucα1-3)(6S)Glcβ-Sp0
**290**	Galβ1-4(Fucα1-3)GlcNAcβ1-3Galβ1-3(Fucα1-4)GlcNAcβ-Sp0
**291**	Galβ1-4GlcNAcβ1-3Galβ1-3GlcNAcβ-Sp0
**292**	Neu5Acα2-3Galβ1-3GlcNAcβ1-3Galβ1-3GlcNAcβ-Sp0
**293**	Neu5Acα2-3Galβ1-4GlcNAcβ1-3Galβ1-3GlcNAcβ-Sp0

**294**	4S(3S)Galβ1-4GlcNAcβ-Sp0
**295**	(6S)Galβ1-4(6S)GlcNAcβ-Sp0
**296**	(6P)Glcβ-Sp10
**297**	Neu5Acα2-3Galβ1-4(Fucα1-3)GlcNAcβ1-6(Galβ1-3)GalNAcα-Sp14
**298**	Galβ1-3Galβ1-4GlcNAcβ-Sp8
**299**	Neu5Acα2-6Galβ1-4GlcNAcβ1-2Manα1-6(Galβ1-4GlcNAcβ1-2Manα1-3)Manβ1-4GlcNAcβ1-4GlcNAcβ-Sp12
**300**	Galβ1-4GlcNAcβ1-6(Galβ1-4GlcNAcβ1-3)Galβ1-4GlcNAc-Sp0
**301**	GlcNAcβ1-6(Galβ1-4GlcNAcβ1-3)Galβ1-4GlcNAc-Sp0
**302**	Galβ1-4GlcNAcα1-6Galβ1-4GlcNAcβ-Sp0
**303**	Galβ1-4GlcNAcβ1-6Galβ1-4GlcNAcβ-Sp0
**304**	GalNAcβ1-3Galβ-Sp8
**305**	GlcAβ1-3GlcNAcβ-Sp8
**306**	Neu5Acα2-6Galβ1-4GlcNAcβ1-2Manα1-6(GlcNAcβ1-2Manα1-3)Manβ1-4GlcNAcβ1-4GlcNAcβ-Sp12
**307**	GlcNAcβ1-3Man-Sp10
**308**	GlcNAcβ1-4GlcNAcβ-Sp10
**309**	GlcNAcβ1-4GlcNAcβ-Sp12
**310**	MurNAcβ1-4GlcNAcβ-Sp10
**311**	Manα1-6Manβ-Sp10
**312**	Manα1-6(Manα1-3)Manα1-6(Manα1-3)Manβ-Sp10
**313**	Manα1-2Manα1-6(Manα1-3)Manα1-6(Manα1-2Manα1-2Manα1-3)Manα-Sp9

**314**	Manα1-2Manα1-6(Manα1-2Manα1-3)Manα1-6(Manα1-2Manα1-2Manα1-3)Manα-Sp9
**315**	Neu5Acα2-3Galβ1-4GlcNAcβ1-6(Neu5Acα2-3Galβ1-3)GalNAcα-Sp14
**316**	Neu5Acα2-6Galβ1-4GlcNAcβ1-2Manα1-6(Neu5Acα2-3Galβ1-4GlcNAcβ1-2Manα1-3)Manβ1-4GlcNAcβ1-4GlcNAcβ-Sp12
**317**	Galβ1-4GlcNAcβ1-2Manα1-6(Neu5Acα2-6Galβ1-4GlcNAcβ1-2Manα1-3)Manβ1-4GlcNAcβ1-4GlcNAcβ-Sp12
**318**	Neu5Acα2-8Neu5Acβ-Sp17
**319**	Neu5Acα2-8Neu5Acα2-8Neu5Acβ-Sp8
**320**	Neu5Gcβ2-6Galβ1-4GlcNAc-Sp8
**321**	Galβ1-3GlcNAcβ1-2Manα1-6(Galβ1-3GlcNAcβ1-2Manα1-3)Manβ1-4GlcNAcβ1-4GlcNAcβ-Sp19
**322**	Neu5Acα2-3Galβ1-4GlcNAcβ1-2Manα1-6(Neu5Acα2-3Galβ1-4GlcNAcβ1-2Manα1-3)Manβ1-4GlcNAcβ1-4GlcNAcβ-Sp12
**323**	Neu5Acα2-3Galβ1-4GlcNAcβ1-2Manα1-6(Neu5Acα2-6Galβ1-4GlcNAcβ1-2Manα1-3)Manβ1-4GlcNAcβ1-4GlcNAcβ-Sp12
**324**	Galβ1-4(Fucα1-3)GlcNAcβ1-2Manα1-6(Galβ1-4(Fucα1-3)GlcNAcβ1-2Manα1-3)Manβ1-4GlcNAcβ1-4GlcNAcβ-Sp20
**325**	Neu5,9Ac2α2-3Galβ1-4GlcNAcβ-Sp0
**326**	Neu5,9Ac2α2-3Galβ1-3GlcNAcβ-Sp0
**327**	Neu5Acα2-6Galβ1-4GlcNAcβ1-3Galβ1-3GlcNAcβ-Sp0
**328**	Neu5Acα2-3Galβ1-3(Fucα1-4)GlcNAcβ1-3Galβ1-3(Fucα1-4)GlcNAcβ-Sp0
**329**	Neu5Acα2-6Galβ1-4GlcNAcβ1-3Galβ1-4GlcNAcβ1-3Galβ1-4GlcNAcβ-Sp0
**330**	Galα1-4Galβ1-4GlcNAcβ1-3Galβ1-4Glcβ-Sp0

**331**	GalNAcβ1-3Galα1-4Galβ1-4GlcNAcβ1-3Galβ1-4Glcβ-Sp0
**332**	GalNAcα1-3(Fucα1-2)Galβ1-4GlcNAcβ1-3Galβ1-4GlcNAcβ-Sp0
**333**	GalNAcα1-3(Fucα1-2)Galβ1-4GlcNAcβ1-3Galβ1-4GlcNAcβ1-3Galβ1-4GlcNAcβ-Sp0
**334**	Neu5Acα2-3Galβ1-4(Fucα1-3)GlcNAcβ1-6(Neu5Acα2-3Galβ1-3)GalNAc-Sp14
**335**	GlcNAcα1-4Galβ1-4GlcNAcβ1-3Galβ1-4GlcNAcβ1-3Galβ1-4GlcNAcβ-Sp0
**336**	GlcNAcα1-4Galβ1-4GlcNAcβ-Sp0
**337**	GlcNAcα1-4Galβ1-3GlcNAcβ-Sp0
**338**	GlcNAcα1-4Galβ1-4GlcNAcβ1-3Galβ1-4Glcβ-Sp0
**339**	GlcNAcα1-4Galβ1-4GlcNAcβ1-3Galβ1-4(Fucα1-3)GlcNAcβ1-3Galβ1-4(Fucα1-3)GlcNAcβ-Sp0
**340**	GlcNAcα1-4Galβ1-4GlcNAcβ1-3Galβ1-4GlcNAcβ-Sp0
**341**	GlcNAcα1-4Galβ1-3GalNAc-Sp14
**342**	Neu5Acα2-6Galβ1-4GlcNAcβ1-2Manα1-6(Manα1-3)Manβ1-4GlcNAcβ1-4GlcNAc-Sp12
**343**	Manα1-6(Neu5Acα2-6Galβ1-4GlcNAcβ1-2Manα1-3)Manβ1-4GlcNAcβ1-4GlcNAc-Sp12
**344**	Neu5Acα2-6Galβ1-4GlcNAcβ1-2Manα1-6Manβ1-4GlcNAcβ1-4GlcNAc-Sp12
**345**	Neu5Acα2-6Galβ1-4GlcNAcβ1-2Manα1-3Manβ1-4GlcNAcβ1-4GlcNAc-Sp12
**346**	Galβ1-4GlcNAcβ1-2Manα1-3Manβ1-4GlcNAcβ1-4GlcNAc-Sp12
**347**	Galβ1-4GlcNAcβ1-2Manα1-6Manβ1-4GlcNAcβ1-4GlcNAc-Sp12

**348**	Manα1-6(Galβ1-4GlcNAcβ1-2Manα1-3)Manβ1-4GlcNAcβ1-4GlcNAcβ-Sp12
**349**	GlcNAcβ1-2Manα1-6(GlcNAcβ1-2Manα1-3)Manβ1-4GlcNAcβ1-4(Fucα1-6)GlcNAcβ-Sp22
**350**	Galβ1-4GlcNAcβ1-2Manα1-6(Galβ1-4GlcNAcβ1-2Manα1-3)Manβ1-4GlcNAcβ1-4(Fucα1-6)GlcNAcβ-Sp22
**351**	Galβ1-3GlcNAcβ1-2Manα1-6(Galβ1-3GlcNAcβ1-2Manα1-3)Manβ1-4GlcNAcβ1-4(Fucα1-6)GlcNAcβ-Sp22
**352**	(6S)GlcNAcβ1-3Galβ1-4GlcNAcβ-Sp0
**353**	KDNα2-3Galβ1-4(Fucα1-3)GlcNAc-Sp0
**354**	KDNα2-6Galβ1-4GlcNAc-Sp0
**355**	KDNα2-3Galβ1-4Glc-Sp0
**356**	KDNα2-3Galβ1-3GalNAcα-Sp14
**357**	Fucα1-2Galβ1-3GlcNAcβ1-2Manα1-6(Fucα1-2Galβ1-3GlcNAcβ1-2Manα1-3)Manβ1-4GlcNAcβ1-4GlcNAcβ-Sp20
**358**	Fucα1-2Galβ1-4GlcNAcβ1-2Manα1-6(Fucα1-2Galβ1-4GlcNAcβ1-2Manα1-3)Manβ1-4GlcNAcβ1-4GlcNAcβ-Sp20
**359**	Fucα1-2Galβ1-4(Fucα1-3)GlcNAcβ1-2Manα1-6(Fucα1-2Galβ1-4(Fucα1-3)GlcNAcβ1-2Manα1-3)Manβ1-4GlcNAcβ1-4GlcNAβ-Sp20
**360**	Galα1-3Galβ1-4GlcNAcβ1-2Manα1-6(Galα1-3Galβ1-4GlcNAcβ1-2Manα1-3)Manβ1-4GlcNAcβ1-4GlcNAcβ-Sp20
**361**	Galβ1-4GlcNAcβ1-2Manα1-6(Manα1-3)Manβ1-4GlcNAcβ1-4GlcNAcβ-Sp12
**362**	Fucα1-4(Galβ1-3)GlcNAcβ1-2Manα1-6(Fucα1-4(Galβ1-3)GlcNAcβ1-2Manα1-3)Manβ1-4GlcNAcβ1-4(Fucα1-6)GlcNAcβ-Sp22

**363**	Neu5Acα2-6GlcNAcβ1-4GlcNAc-Sp21
**364**	Neu5Acα2-6GlcNAcβ1-4GlcNAcβ1-4GlcNAc-Sp21
**365**	Galβ1-4(Fucα1-3)GlcNAcβ1-6(Fucα1-2Galβ1-4GlcNAcβ1-3)Galβ1-4Glc-Sp21
**366**	Galβ1-4GlcNAcβ1-2Manα1-6(Galβ1-4GlcNAcβ1-4(Galβ1-4GlcNAcβ1-2)Manα1-3)Manβ1-4GlcNAcβ1-4GlcNAc-Sp21
**367**	GalNAcα1-3(Fucα1-2)Galβ1-4GlcNAcβ1-2Manα1-6(GalNAcα1-3(Fucα1-2)Galβ1-4GlcNAcβ1-2Manα1-3)Manβ1-4GlcNAcβ1-4GlcNAcβ-Sp20
**368**	Galα1-3(Fucα1-2)Galβ1-4GlcNAcβ1-2Manα1-6(Galα1-3(Fucα1-2)Galβ1-4GlcNAcβ1-2Manα1-3)Manβ1-4GlcNAcβ1-4GlcNAcβ-Sp20
**369**	Galα1-3Galβ1-4(Fucα1-3)GlcNAcβ1-2Manα1-6(Galα1-3Galβ1-4(Fucα1-3)GlcNAcβ1-2Manα1-3)Manβ1-4GlcNAcβ1-4GlcNAcβ-Sp20
**370**	GalNAcα1-3(Fucα1-2)Galβ1-3GlcNAcβ1-2Manα1-6(GalNAcα1-3(Fucα1-2)Galβ1-3GlcNAcβ1-2Manα1-3)Manβ1-4GlcNAcβ1-4GlcNAcβ-Sp20
**371**	Fucα1-4(Fucα1-2Galβ1-3)GlcNAcβ1-2Manα1-3(Fucα1-4(Fucα1-2Galβ1-3)GlcNAcβ1-2Manα1-3)Manβ1-4GlcNAcβ1-4GlcNAcβ-Sp19
**372**	Neu5Acα2-3Galβ1-4GlcNAcβ1-3GalNAc-Sp14
**373**	Neu5Acα2-6Galβ1-4GlcNAcβ1-3GalNAc-Sp14
**374**	Neu5Acα2-3Galβ1-4(Fucα1-3)GlcNAcβ1-3GalNAcα-Sp14
**375**	GalNAcβ1-4GlcNAcβ1-2Manα1-6(GalNAcβ1-4GlcNAcβ1-2Manα1-3)Manβ1-4GlcNAcβ1-4GlcNAc-Sp12
**376**	Galβ1-3GalNAcα1-3(Fucα1-2)Galβ1-4Glc-Sp0
**377**	Galβ1-3GalNAcα1-3(Fucα1-2)Galβ1-4GlcNAc-Sp0
**378**	Galβ1-3GlcNAcβ1-3Galβ1-4GlcNAcβ1-6(Galβ1-3GlcNAcβ1-3)Galβ1-4Glcβ-Sp0

**379**	Galβ1-4(Fucα1-3)GlcNAcβ1-6(Galβ1-3GlcNAcβ1-3)Galβ1-4Glc-Sp21
**380**	Galβ1-4GlcNAcβ1-6(Fucα1-4(Fucα1-2Galβ1-3)GlcNAcβ1-3)Galβ1-4Glc-Sp21
**381**	Galβ1-4(Fucα1-3)GlcNAcβ1-6(Fucα1-4(Fucα1-2Galβ1-3)GlcNAcβ1-3)Galβ1-4Glc-Sp21
**382**	Galβ1-3GlcNAcβ1-3Galβ1-4(Fucα1-3)GlcNAcβ1-6(Galβ1-3GlcNAcβ1-3)Galβ1-4Glc-Sp21
**383**	Galβ1-4GlcNAcβ1-6(Galβ1-4GlcNAcβ1-2)Manα1-6(Galβ1-4GlcNAcβ1-4(Galβ1-4GlcNAcβ1-2)Manα1-3)Manβ1-4GlcNAcβ1-4GlcNAcβ-Sp21
**384**	GlcNAcβ1-2Manα1-6(GlcNAcβ1-4(GlcNAcβ1-2)Manα1-3)Manβ1-4GlcNAcβ1-4GlcNAc-Sp21
**385**	Fucα1-2Galβ1-3GalNAcα1-3(Fucα1-2)Galβ1-4Glcβ-Sp0
**386**	Fucα1-2Galβ1-3GalNAcα1-3(Fucα1-2)Galβ1-4GlcNAcβ-Sp0
**387**	Galβ1-3GlcNAcβ1-3GalNAcα-Sp14
**388**	GalNAcβ1-4(Neu5Acα2-3)Galβ1-4GlcNAcβ1-3GalNAcα-Sp14
**389**	GalNAcα1-3(Fucα1-2)Galβ1-3GalNAcα1-3(Fucα1-2)Galβ1-4GlcNAcβ-Sp0
**390**	Galα1-3Galβ1-3GlcNAcβ1-2Manα1-6(Galα1-3Galβ1-3GlcNAcβ1-2Manα1-3)Manβ1-4GlcNAcβ1-4GlcNAc-Sp19
**391**	Galα1-3Galβ1-3(Fucα1-4)GlcNAcβ1-2Manα1-6(Galα1-3Galβ1-3(Fucα1-4)GlcNAcβ1-2Manα1-3)Manβ1-4GlcNAcβ1-4GlcNAc-Sp19
**392**	Neu5Acα2-3Galβ1-3GlcNAcβ1-2Manα1-6(Neu5Acα2-3Galβ1-3GlcNAcβ1-2Manα1-3)Manβ1-4GlcNAcβ1-4GlcNAc-Sp19
**393**	GlcNAcβ1-2Manα1-6(Galβ1-4GlcNAcβ1-2Manα1-3)Manβ1-4GlcNAcβ1-4GlcNAc-Sp12
**394**	Galβ1-4GlcNAcβ1-2Manα1-6(GlcNAcβ1-2Manα1-3)Manβ1-4GlcNAcβ1-

	4GlcNAc-Sp12
**395**	Neu5Acα2-3Galβ1-3GlcNAcβ1-3GalNAcα-Sp14
**396**	Fucα1-2Galβ1-4GlcNAcβ1-3GalNAcα-Sp14
**397**	Galβ1-4(Fucα1-3)GlcNAcβ1-3GalNAcα-Sp14
**398**	GalNAcα1-3GalNAcβ1-3Galα1-4Galβ1-4GlcNAcβ-Sp0
**399**	Galα1-4Galβ1-3GlcNAcβ1-2Manα1-6(Galα1-4Galβ1-3GlcNAcβ1-2Manα1-3)Manβ1-4GlcNAcβ1-4GlcNAcβ-Sp19
**400**	Galα1-4Galβ1-4GlcNAcβ1-2Manα1-6(Galα1-4Galβ1-4GlcNAcβ1-2Manα1-3)Manβ1-4GlcNAcβ1-4GlcNAcβ-Sp24
**401**	Galα1-3Galβ1-4GlcNAcβ1-3GalNAcα-Sp14
**402**	Galβ1-3GlcNAcβ1-6Galβ1-4GlcNAcβ-Sp0
**403**	Galβ1-3GlcNAcα1-6Galβ1-4GlcNAcβ-Sp0
**404**	GalNAcβ1-3Galα1-6Galβ1-4Glcβ-Sp8
**405**	Galα1-3(Fucα1-2)Galβ1-4(Fucα1-3)Glcβ-Sp21
**406**	Galβ1-4GlcNAcβ1-6(Neu5Acα2-6Galβ1-3GlcNAcβ1-3)Galβ1-4Glc-Sp21
**407**	Galβ1-3GalNAcβ1-4(Neu5Acα2-8Neu5Acα2-3)Galβ1-4Glcβ-Sp0
**408**	Neu5Acα2-3Galβ1-3GalNAcβ1-4(Neu5Acα2-8Neu5Acα2-3)Galβ1-4Glcβ-Sp0
**409**	Galα1-3(Fucα1-2)Galβ1-4GlcNAcβ1-3GalNAcα-Sp14
**410**	GalNAcα1-3(Fucα1-2)Galβ1-4GlcNAcβ1-3GalNAcα-Sp14
**411**	GalNAcα1-3GalNAcβ1-3Galα1-4Galβ1-4Glcβ-Sp0
**412**	Fucα1-2Galβ1-4(Fucα1-3)GlcNAcβ1-3GalNAcα-Sp14
**413**	Galα1-3(Fucα1-2)Galβ1-4(Fucα1-3)GlcNAcβ1-3GalNAc-Sp14

**414**	GalNAcα1-3(Fucα1-2)Galβ1-4(Fucα1-3)GlcNAcβ1-3GalNAc-Sp14
**415**	Galβ1-4(Fucα1-3)GlcNAcβ1-2Manα1-6(Galβ1-4(Fucα1-3)GlcNAcβ1-2Manα1-3)Manβ1-4GlcNAcβ1-4(Fucα1-6)GlcNAcβ-Sp22
**416**	Fucα1-2Galβ1-4GlcNAcβ1-2Manα1-6(Fucα1-2Galβ1-4GlcNAcβ1-2Manα1-3)Manβ1-4GlcNAcβ1-4(Fucα1-6)GlcNAcβ-Sp22
**417**	GlcNAcβ1-2(GlcNAcβ1-6)Manα1-6(GlcNAcβ1-2Manα1-3)Manβ1-4GlcNAcβ1-4GlcNAcβ-Sp19
**418**	Fucα1-2Galβ1-3GlcNAcβ1-3GalNAc-Sp14
**419**	Galα1-3(Fucα1-2)Galβ1-3GlcNAcβ1-3GalNAc-Sp14
**420**	GalNAcα1-3(Fucα1-2)Galβ1-3GlcNAcβ1-3GalNAc-Sp14
**421**	Galα1-3Galβ1-3GlcNAcβ1-3GalNAc-Sp14
**422**	Fucα1-2Galβ1-3GlcNAcβ1-2Manα1-6(Fucα1-2Galβ1-3GlcNAcβ1-2Manα1-3)Manβ1-4GlcNAcβ1-4(Fucα1-6)GlcNAcβ-Sp22
**423**	Galα1-3(Fucα1-2)Galβ1-4GlcNAcβ1-2Manα1-6(Galα1-3(Fucα1-2)Galβ1-4GlcNAcβ1-2Manα1-3)Manβ1-4GlcNAcβ1-4(Fucα1-6)GlcNAcβ-Sp22
**424**	Galβ1-3GlcNAcβ1-6(Galβ1-3GlcNAcβ1-2)Manα1-6(Galβ1-3GlcNAcβ1-2Manα1-3)Manβ1-4GlcNAcβ1-4GlcNAcβ-Sp19
**425**	Galβ1-4GlcNAcβ1-6(Fucα1-2Galβ1-3GlcNAcβ1-3)Galβ1-4Glc-Sp21
**426**	Fucα1-3GlcNAcβ1-6(Galβ1-4GlcNAcβ1-3)Galβ1-4Glc-Sp21
**427**	GlcNAcβ1-2Manα1-6(GlcNAcβ1-4)(GlcNAcβ1-2Manα1-3)Manβ1-4GlcNAcβ1-4GlcNAc-Sp21
**428**	GlcNAcβ1-2Manα1-6(GlcNAcβ1-4)(GlcNAcβ1-4(GlcNAcβ1-2)Manα1-3)Manβ1-4GlcNAcβ1-4GlcNAc-Sp21
**429**	GlcNAcβ1-6(GlcNAcβ1-2)Manα1-6(GlcNAcβ1-4)(GlcNAcβ1-2Manα1-3)Manβ1-4GlcNAcβ1-4GlcNAc-Sp21

**430**	GlcNAcβ1-6(GlcNAcβ1-2)Manα1-6(GlcNAcβ1-4)(GlcNAcβ1-4(GlcNAcβ1-2)Manα1-3)Manβ1-4GlcNAcβ1-4GlcNAc-Sp21
**431**	Galβ1-4GlcNAcβ1-2Manα1-6(GlcNAcβ1-4)(Galβ1-4GlcNAcβ1-2Manα1-3)Manβ1-4GlcNAcβ1-4GlcNAc-Sp21
**432**	Galβ1-4GlcNAcβ1-2Manα1-6(GlcNAcβ1-4)(Galβ1-4GlcNAcβ1-4(Galβ1-4GlcNAcβ1-2)Manα1-3)Manβ1-4GlcNAcβ1-4GlcNAc-Sp21
**433**	Galβ1-4GlcNAcβ1-6(Galβ1-4GlcNAcβ1-2)Manα1-6(GlcNAcβ1-4)(Galβ1-4GlcNAcβ1-2Manα1-3)Manβ1-4GlcNAcβ1-4GlcNAc-Sp21
**434**	Galβ1-4GlcNAcβ1-6(Galβ1-4GlcNAcβ1-2)Manα1-6(GlcNAcβ1-4)(Galβ1-4GlcNAcβ1-4(Galβ1-4GlcNAcβ1-2)Manα1-3)Manβ1-4GlcNAcβ1-4GlcNAc-Sp21
**435**	Galβ1-4Galβ-Sp10
**436**	Galβ1-6Galβ-Sp10
**437**	Neu5Acα2-3Galβ1-4GlcNAcβ1-3Galβ-Sp8
**438**	GalNAcβ1-6GalNAcβ-Sp8
**439**	(6S)Galβ1-3GlcNAcβ-Sp0
**440**	(6S)Galβ1-3(6S)GlcNAc-Sp0
**441**	Fucα1-2Galβ1-4 GlcNAcβ1-2Manα1-6(Fucα1-2Galβ1-4GlcNAcβ1-2(Fucα1-2Galβ1-4GlcNAcβ1-4)Manα1-3)Manβ1-4GlcNAcβ1-4GlcNAcβ-Sp12
**442**	Fucα1-2Galβ1-4(Fucα1-3)GlcNAcβ1-2Manα1-6(Fucα1-2Galβ1-4(Fucα1-3)GlcNAcβ1-4(Fucα1-2Galβ1-4(Fucα1-3)GlcNAcβ1-2)Manα1-3)Manβ1-4GlcNAcβ1-4GlcNAcβ-Sp12
**443**	Galβ1-4(Fucα1-3)GlcNAcβ1-6GalNAc-Sp14
**444**	Galβ1-4GlcNAcβ1-2Manα-Sp0
**445**	Fucα1-2Galβ1-4GlcNAcβ1-6(Fucα1-2Galβ1-4GlcNAcβ1-3)GalNAc-Sp14

**446**	Galα1-3(Fucα1-2)Galβ1-4GlcNAcβ1-6(Galα1-3(Fucα1-2)Galβ1-4GlcNAcβ1-3)GalNAc-Sp14
**447**	GalNAcα1-3(Fucα1-2)Galβ1-4GlcNAcβ1-6(GalNAcα1-3(Fucα1-2)Galβ1-4GlcNAcβ1-3)GalNAc-Sp14
**448**	Neu5Acα2-8Neu5Acα2-3Galβ1-3GalNAcβ1-4(Neu5Acα2-8Neu5Acα2-3)Galβ1-4Glcβ-Sp0
**449**	GalNAcβ1-4Galβ1-4Glcβ-Sp0
**450**	GalNAcα1-3(Fucα1-2)Galβ1-4GlcNAcβ1-2Manα1-6(GalNAcα1-3(Fucα1-2)Galβ1-4GlcNAcβ1-2Manα1-3)Manβ1-4GlcNAcβ1-4(Fucα1-6)GlcNAcβ-Sp22
**451**	Galα1-3(Fucα1-2)Galβ1-3GlcNAcβ1-2Manα1-6(Galα1-3(Fucα1-2)Galβ1-3GlcNAcβ1-2Manα1-3)Manβ1-4GlcNAcβ1-4(Fucα1-6)GlcNAcβ-Sp22
**452**	Neu5Acα2-6Galβ1-4GlcNAcβ1-6(Fucα1-2Galβ1-3GlcNAcβ1-3)Galβ1-4Glc-Sp21
**453**	GalNAcα1-3(Fucα1-2)Galβ1-3GlcNAcβ1-2Manα1-6(GalNAcα1-3(Fucα1-2)Galβ1-3GlcNAcβ1-2Manα1-3)Manβ1-4GlcNAcβ1-4(Fucα1-6)GlcNAcβ-Sp22
**454**	Galβ1-4GlcNAcβ1-6(Galβ1-4GlcNAcβ1-2)Manα1-6(Galβ1-4GlcNAcβ1-2Manα1-3)Manβ1-4GlcNAcβ1-4GlcNAcβ-Sp19
**455**	Neu5Acα2-3Galβ1-4GlcNAcβ1-2Manα1-6(GlcNAcβ1-4)(Neu5Acα2-3Galβ1-4GlcNAcβ1-2Manα1-3)Manβ1-4GlcNAcβ1-4GlcNAcβ-Sp21
**456**	Neu5Acα2-3Galβ1-4GlcNAcβ1-4Manα1-6(GlcNAcβ1-4)(Neu5Acα2-3Galβ1-4GlcNAcβ1-4(Neu5Acα2-3Galβ1-4GlcNAcβ1-2)Manα1-3)Manβ1-4GlcNAcβ1-4GlcNAcβ-Sp21
**457**	Neu5Acα2-3Galβ1-4GlcNAcβ1-6(Neu5Acα2-3Galβ1-4GlcNAcβ1-2)Manα1-6(GlcNAcβ1-4)(Neu5Acα2-3Galβ1-4GlcNAcβ1-2Manα1-3)Manβ1-4GlcNAcβ1-4GlcNAcβ-Sp21

**458**	Neu5Acα2-3Galβ1-4GlcNAcβ1-6(Neu5Acα2-3Galβ1-4GlcNAcβ1-2)Manα1-6(GlcNAcβ1-4)(Neu5Acα2-3Galβ1-4GlcNAcβ1-4(Neu5Acα2-3Galβ1-4GlcNAcβ1-2)Manα1-3)Manβ1-4GlcNAcβ1-4GlcNAcβ-Sp21
**459**	Neu5Acα2-6Galβ1-4GlcNAcβ1-2Manα1-6(GlcNAcβ1-4)(Neu5Acα2-6Galβ1-4GlcNAcβ1-2Manα1-3)Manβ1-4GlcNAcβ1-4GlcNAcβ-Sp21
**460**	Neu5Acα2-6Galβ1-4GlcNAcβ1-4Manα1-6(GlcNAcβ1-4)(Neu5Acα2-6Galβ1-4GlcNAcβ1-4(Neu5Acα2-6Galβ1-4GlcNAcβ1-2)Manα1-3)Manβ1-4GlcNAcβ1-4GlcNAcβ-Sp21
**461**	Neu5Acα2-6Galβ1-4GlcNAcβ1-6(Neu5Acα2-6Galβ1-4GlcNAcβ1-2)Manα1-6(GlcNAcβ1-4)(Neu5Acα2-6Galβ1-4GlcNAcβ1-2Manα1-3)Manβ1-4GlcNAcβ1-4GlcNAcβ-Sp21
**462**	Neu5Acα2-6Galβ1-4GlcNAcβ1-6(Neu5Acα2-6Galβ1-4GlcNAcβ1-2)Manα1-6(GlcNAcβ1-4)(Neu5Acα2-6Galβ1-4GlcNAcβ1-4(Neu5Acα2-6Galβ1-4GlcNAcβ1-2)Manα1-3)Manβ1-4GlcNAcβ1-4GlcNAcβ-Sp21
**463**	Galα1-3(Fucα1-2)Galβ1-3GalNAcα-Sp8
**464**	Galα1-3(Fucα1-2)Galβ1-3GalNAcβ-Sp8
**465**	Glcα1-6Glcα1-6Glcα1-6Glcβ-Sp10
**466**	Glcα1-4Glcα1-4Glcα1-4Glcβ-Sp10
**467**	Neu5Acα2-3Galβ1-4GlcNAcβ1-6(Neu5Acα2-3Galβ1-4GlcNAcβ1-3)GalNAcα-Sp14
**468**	Fucα1-2Galβ1-4(Fucα1-3)GlcNAcβ1-2Manα1-6(Fucα1-2Galβ1-4(Fucα1-3)GlcNAcβ1-2Manα1-3)Manβ1-4GlcNAcβ1-4(Fucα1-6)GlcNAcβ-Sp24
**469**	Fucα1-2Galβ1-3(Fucα1-4)GlcNAcβ1-2Manα1-6(Fucα1-2Galβ1-3(Fucα1-4)GlcNAcβ1-2Manα1-3)Manβ1-4GlcNAcβ1-4(Fucα1-6)GlcNAcβ1-4(Fucα1-6)GlcNAcβ-Sp19
**470**	GlcNAcβ1-6(GlcNAcβ1-2)Manα1-6(GlcNAcβ1-2Manα1-3)Manβ1-4GlcNAcβ1-4(Fucα1-6)GlcNAcβ-Sp24

**471**	Galβ1-3GlcNAcβ1-2Manα1-6(GlcNAcβ1-4)(Galβ1-3GlcNAcβ1-2Manα1-3)Manβ1-4GlcNAcβ1-4GlcNAcβ-Sp21
**472**	Neu5Acα2-6Galβ1-4GlcNAcβ1-6(Galβ1-3GlcNAcβ1-3)Galβ1-4Glcβ-Sp21
**473**	Neu5Acα2-3Galβ1-4GlcNAcβ1-2Manα-Sp0
**474**	Neu5Acα2-3Galβ1-4GlcNAcβ1-6GalNAcα-Sp14
**475**	Neu5Acα2-6Galβ1-4GlcNAcβ1-6GalNAcα-Sp14
**476**	Neu5Acα2-6Galβ1-4 GlcNAcβ1-6(Neu5Acα2-6Galβ1-4GlcNAcβ1-3)GalNAcα-Sp14
**477**	Neu5Acα2-6Galβ1-4GlcNAcβ1-2Manα1-6(Neu5Acα2-6Galβ1-4GlcNAcβ1-2Manα1-3)Manβ1-4GlcNAcβ1-4(Fucα1-6)GlcNAcβ-Sp24
**478**	Neu5Acα2-3Galβ1-4GlcNAcβ1-2Manα1-6(Neu5Acα2-3Galβ1-4GlcNAcβ1-2Manα1-3)Manβ1-4GlcNAcβ1-4(Fucα1-6)GlcNAcβ-Sp24
**479**	Manα1-6(Manα1-3)Manβ1-4GlcNAcβ1-4(Fucα1-6)GlcNAcβ-Sp19
**480**	Galβ1-4GlcNAcβ1-6(Galβ1-4GlcNAcβ1-2)Manα1-6(Galβ1-4GlcNAcβ1-2Manα1-3)Manβ1-4GlcNAcβ1-4(Fucα1-6)GlcNAcβ-Sp24
**481**	Neu5Acα2-3Galβ1-3GlcNAcβ1-2Manα1-6(GlcNAcβ1-4)(Neu5Acα2-3Galβ1-3GlcNAcβ1-2Manα1-3)Manβ1-4GlcNAcβ1-4GlcNAc-Sp21
**482**	Neu5Acα2-6Galβ1-4GlcNAcβ1-6(Fucα1-2Galβ1-4(Fucα1-3)GlcNAcβ1-3)Galβ1-4Glc-Sp21
**483**	Galβ1-3GlcNAcβ1-6GalNAcα-Sp14
**484**	Galα1-3Galβ1-3GlcNAcβ1-6GalNAcα-Sp14
**485**	Galβ1-3(Fucα1-4)GlcNAcβ1-6GalNAcα-Sp14
**486**	Neu5Acα2-3Galβ1-3GlcNAcβ1-6GalNAcα-Sp14
**487**	(3S)Galβ1-3(Fucα1-4)GlcNAcβ-Sp0

**488**	Galβ1-4(Fucα1-3)GlcNAcβ1-6(Neu5Acα2-6(Neu5Acα2-3Galβ1-3)GlcNAcβ1-3)Galβ1-4Glc-Sp21
**489**	Fucα1-2Galβ1-4GlcNAcβ1-6GalNAcα-Sp14
**490**	Galα1-3Galβ1-4GlcNAcβ1-6GalNAcα-Sp14
**491**	Galβ1-4(Fucα1-3)GlcNAcβ1-2Manα-Sp0
**492**	Fucα1-2(6S)Galβ1-3GlcNAcβ-Sp0
**493**	Galα1-3(Fucα1-2)Galβ1-4GlcNAcβ1-6GalNAcα-Sp14
**494**	Fucα1-2Galβ1-4GlcNAcβ1-2Manα-Sp0
**495**	Fucα1-2Galβ1-3(6S)GlcNAcβ-Sp0
**496**	Fucα1-2(6S)Galβ1-3(6S)GlcNAcβ-Sp0
**497**	Neu5Acα2-6GalNAcβ1-4(6S)GlcNAcβ-Sp8
**498**	GalNAcβ1-4(Fucα1-3)(6S)GlcNAcβ-Sp8
**499**	(3S)GalNAcβ1-4(Fucα1-3)GlcNAcβ-Sp8
**500**	Fucα1-2Galβ1-3GlcNAcβ1-6(Fucα1-2Galβ1-3GlcNAcβ1-3)GalNAcα-Sp14
**501**	GalNAcα1-3(Fucα1-2)Galβ1-3GlcNAcβ1-6GalNAcα-Sp14
**502**	GlcNAcβ1-6(GlcNAcβ1-2)Manα1-6(GlcNAcβ1-4)(GlcNAcβ1-4(GlcNAcβ1-2)Manα1-3)Manβ1-4GlcNAcβ1-4(Fucα1-6)GlcNAc-Sp21
**503**	Galβ1-4GlcNAcβ1-6(Galβ1-4GlcNAcβ1-2)Manα1-6(GlcNAcβ1-4)Galβ1-4GlcNAcβ1-4(Gal b1-4GlcNAcβ1-2)Manα1-3)Manβ1-4GlcNAcβ1-4(Fucα1-6)GlcNAc-Sp21
**504**	Galβ1-3GlcNAcα1-3Galβ1-4GlcNAcβ-Sp8
**505**	Galβ1-3(6S)GlcNAcβ-Sp8
**506**	(6S)(4S)GalNAcβ1-4GlcNAc-Sp8

**507**	(6S)GalNAcβ1-4GlcNAc-Sp8
**508**	(3S)GalNAcβ1-4(3S)GlcNAc-Sp8
**509**	GalNAcβ1-4(6S)GlcNAc-Sp8
**510**	(3S)GalNAcβ1-4GlcNAc-Sp8
**511**	(4S)GalNAcβ-Sp10
**512**	Galβ1-4(6P)GlcNAcβ-Sp0
**513**	(6P)Galβ1-4GlcNAcβ-SP0
**514**	GalNAcα1-3(Fucα1-2)Galβ1-4GlcNAcβ1-6GalNAc-Sp14
**515**	Neu5Acα2-6Galβ1-4GlcNAcβ1-2Man-Sp0
**516**	Galα1-3Galβ1-4GlcNAcβ1-2Manα-Sp0
**517**	Galα1-3(Fucα1-2)Galβ1-4GlcNAcβ1-2Manα-Sp0
**518**	GalNAcα1-3(Fucα1-2)Galβ1-4 GlcNAcβ1-2Manα-Sp0
**519**	Galβ1-3GlcNAcβ1-2Manα-Sp0
**520**	Galα1-3(Fucα1-2)Galβ1-3GlcNAcβ1-6GalNAc-Sp14
**521**	Neu5Acα2-3Galβ1-3GlcNAcβ1-2Manα-Sp0
**522**	Galα1-3Galβ1-3GlcNAcβ1-2Manα-Sp0
**523**	GalNAcβ1-4GlcNAcβ1-2Manα-Sp0
**524**	Neu5Acα2-3Galβ1-3GalNAcβ1-4Galβ1-4Glcβ-Sp0
**525**	GlcNAcβ1-2 Manα1-6(GlcNAcβ1-4)(GlcNAcβ1-2Manα1-3)Manβ1-4GlcNAcβ1-4(Fucα1-6)GlcNAc-Sp21
**526**	Galβ1-4GlcNAcβ1-2 Manα1-6(GlcNAcβ1-4)(Galβ1-4GlcNAcβ1-2Manα1-3)Manβ1-4GlcNAcβ1-4(Fucα1-6)GlcNAc-Sp21
**527**	Galβ1-4GlcNAcβ1-2 Manα1-6(Galβ1-4GlcNAcβ1-4)(Galβ1-4GlcNAcβ1-

	2Manα1-3)Manβ1-4GlcNAcβ1-4(Fucα1-6)GlcNAc-Sp21
**528**	Fucα1-4(Galβ1-3)GlcNAcβ1-2 Manα-Sp0
**529**	Neu5Acα2-3Galβ1-4(Fucα1-3)GlcNAcβ1-2Manα-Sp0
**530**	GlcNAcβ1-3Galβ1-4GlcNAcβ1-6(GlcNAcβ1-3)Galβ1-4GlcNAc-Sp0
**531**	GalNAcα1-3(Fucα1-2)Galβ1-3GalNAcβ1-3Galα1-4Galβ1-4Glc-Sp21
**532**	Galα1-3(Fucα1-2)Galβ1-3GalNAcβ1-3Galα1-4Galβ1-4Glc-Sp21
**533**	Galβ1-3GalNAcβ1-3Gal-Sp21
**534**	GlcNAcβ1-3Galβ1-4GlcNAcβ1-2Manα1-6(GlcNAcβ1-3Galβ1-4GlcNAcβ1-2Manα1-3)Manβ1-4GlcNAcβ1-4GlcNAcβ-Sp12
**535**	GlcNAcβ1-3Galβ1-4GlcNAcβ1-2Manα1-6(GlcNAcβ1-3Galβ1-4GlcNAcβ1-2Manα1-3)Manβ1-4GlcNAcβ1-4GlcNAcβ-Sp25
**536**	Fucα1-2Galβ1-4GlcNAcβ1-3Galβ1-4GlcNAcβ1-2Manα1-6(Fucα1-2Galβ1-4GlcNAcβ1-3Galβ1-4GlcNAcβ1-2Manα1-3)Manβ1-4GlcNAcβ1-4GlcNAcβ-Sp24
**537**	GlcNAcβ1-3Galβ1-4GlcNAcβ1-3Galβ1-4GlcNAcβ1-2Manα1-6(GlcNAcβ1-3Galβ1-4GlcNAcβ1-3Galβ1-4GlcNAcβ1-2Manα1-3)Manβ1-4GlcNAcβ1-4GlcNAcβ-Sp12
**538**	GlcNAcβ1-3Galβ1-4GlcNAcβ1-3Galβ1-4GlcNAcβ1-2Manα1-6(GlcNAcβ1-3Galβ1-4GlcNAcβ1-3Galβ1-4GlcNAcβ1-2Manα1-3)Manβ1-4GlcNAcβ1-4GlcNAcβ-Sp25
**539**	Galβ1-4GlcNAcβ1-3Galβ1-4GlcNAcβ1-3Galβ1-4GlcNAcβ1-2Manα1-6(Galβ1-4GlcNAcβ1-3Galβ1-4GlcNAcβ1-3Galβ1-4GlcNAcβ1-2Manα1-3)Manβ1-4GlcNAcβ1-4GlcNAcβ-Sp12
**540**	Galβ1-4GlcNAcβ1-3Galβ1-4GlcNAcβ1-3Galβ1-4GlcNAcβ1-2Manα1-6(Galβ1-4GlcNAcβ1-3Galβ1-4GlcNAcβ1-3Galβ1-4GlcNAcβ1-2Manα1-3)Manβ1-4GlcNAcβ1-4GlcNAcβ-Sp24

**541**	Galβ1-3GlcNAcβ1-3Galβ1-4GlcNAcβ1-2Manα1-6(Galβ1-3GlcNAcβ1-3Galβ1-4GlcNAcβ1-2Manα1-3)Manβ1-4GlcNAcβ1-4GlcNAc-Sp25
**542**	Neu5Gcα2-8Neu5Gcα2-3Galβ1-4GlcNAc-Sp0
**543**	Neu5Acα2-8Neu5Gcα2-3Galβ1-4GlcNAc-Sp0
**544**	Neu5Gcα2-8Neu5Acα2-3Galβ1-4GlcNAc-Sp0
**545**	Neu5Gcα2-8Neu5Gcα2-3Galβ1-4GlcNAcβ1-3Galβ1-4GlcNAc-Sp0
**546**	Neu5Gcα2-8Neu5Gcα2-6Galβ1-4GlcNAc-Sp0
**547**	Neu5Acα2-8Neu5Acα2-3Galβ1-4GlcNAc-Sp0
**548**	GlcNAcβ1-3Galβ1-4GlcNAcβ1-6(GlcNAcβ1-3Galβ1-4GlcNAcβ1-2)Manα1-6(GlcNAcβ1-3Galβ1-4GlcNAcβ1-2Manα1-3)Manβ1-4GlcNAcβ1-4GlcNAc-Sp24
**549**	Galβ1-4GlcNAcβ1-3Galβ1-4GlcNAcβ1-6(Galβ1-4GlcNAcβ1-3Galβ1-4GlcNAcβ1-2)Manα1-6(Galβ1-4GlcNAcβ1-3Galβ1-4GlcNAcβ1-2Manα1-3)Manα1-4GlcNAcβ1-4GlcNAc-Sp24
**550**	Galα1-3Galβ1-4GlcNAcβ1-2Manα1-6(Galα1-3Galβ1-4GlcNAcβ1-2Manα1-3)Manβ1-4GlcNAcβ1-4GlcNAc-Sp24
**551**	GlcNAcβ1-3Galβ1-4GlcNAcβ1-6(GlcNAcβ1-3Galβ1-3)GalNAcα-Sp14
**552**	GalNAcβ1-3GlcNAcβ-Sp0
**553**	GalNAcβ1-4GlcNAcβ1-3GalNAcβ1-4GlcNAcβ-Sp0
**554**	GlcNAcβ1-3Galβ1-4GlcNAcβ1-3Galβ1-4GlcNAcβ1-3Galβ1-4GlcNAcβ1-3Galβ1-4GlcNAcβ1-2Manα1-6(GlcNAcβ1-3Galβ1-4GlcNAcβ1-3Galβ1-4GlcNAcβ1-3Galβ1-4GlcNAcβ1-3Galβ1-4GlcNAcβ1-2Manα1-3)Manβ1-4GlcNAcβ1-4GlcNAcβ-Sp25
**555**	Galβ1-4GlcNAcβ1-3Galβ1-4GlcNAcβ1-3Galβ1-4GlcNAcβ1-3Galβ1-4GlcNAcβ1-3Galβ1-4GlcNAcβ1-2Manα1-6(Galβ1-4GlcNAcβ1-3Galβ1-4GlcNAcβ1-3Galβ1-4GlcNAcβ1-3Galβ1-4GlcNAcβ1-3Galβ1-4GlcNAcβ1-

	2Manα1-3)Manβ1-4GlcNAcβ1-4GlcNAcβ-Sp25
**556**	GlcNAβ1-3Galβ1-3GalNAc-Sp14
**557**	Galβ1-3GlcNAcβ1-6(Galβ1-3)GalNAc-Sp14
**558**	Galβ1-4GlcNAcβ1-3Galβ1-4GlcNAcβ1-3Galβ1-4GlcNAcβ1-3Galβ1-4GlcNAcβ1-3Galβ1-4GlcNAcβ1-3Galβ1-4GlcNAcβ1-2Manα1-6(Galβ1-4GlcNAcβ1-3Galβ1-4GlcNAcβ1-3Galβ1-4GlcNAcβ1-3Galβ1-4GlcNAcβ1-3Galβ1-4GlcNAcβ1-3Galβ1-4GlcNAcβ1-2Manα1-3)Manβ1-4GlcNAcβ1-4GlcNAcβ-Sp25
**559**	(3S)GlcAβ1-3Galβ1-4GlcNAcβ1-3Galβ1-4Glc-Sp0
**560**	(3S)GlcAβ1-3Galβ1-4GlcNAcβ1-2Manα-Sp0
**561**	Galβ1-3GlcNAcβ1-3Galβ1-4GlcNAcβ1-3Galβ1-4GlcNAcβ1-6(Galβ1-3GlcNAcβ1-3Galβ1-4GlcNAcβ1-3Galβ1-4GlcNAβ1-2)Manα1-6(Galβ1-3GlcNAcβ1-3Galβ1-4GlcNAcβ1-3Galβ1-4GlcNAcβ1-2Manα1-3)Manβ1-4GlcNAcβ1-4(Fucα1-6)GlcNAcβ-Sp24
**562**	Galβ1-3GlcNAcβ1-3Galβ1-4GlcNAcβ1-6(Galβ1-3GlcNAcβ1-3Galβ1-4GlcNAβ1-2)Manα1-6(Galβ1-3GlcNAcβ1-3Galβ1-4GlcNAcβ1-2Manα1-3)Manβ1-4GlcNAcβ1-4(Fucα1-6)GlcNAcβ-Sp24
**563**	Neu5Acα2-8Neu5Acα2-3Galβ1-3GalNAcβ1-4(Neu5Acα2-3)Galβ1-4Glc-Sp21
**564**	GlcNAcβ1-3Galβ1-4GlcNAcβ1-2Manα1-6(GlcNAcβ1-3Galβ1-4GlcNAcβ1-2Manα1-3)Manβ1-4GlcNAcβ1-4(Fucα1-6)GlcNAcβ-Sp24
**565**	Galβ1-4GlcNAcβ1-3Galβ1-4GlcNAcβ1-2Manα1-6(Galβ1-4GlcNAcβ1-3Galβ1-4GlcNAcβ1-2Manα1-3)Manβ1-4GlcNAcβ1-4(Fucα1-6)GlcNAcβ-Sp24
**566**	GlcNAcβ1-3Galβ1-4GlcNAcβ1-3Galβ1-4GlcNAcβ1-2Manα1-6(GlcNAcβ1-3Galβ1-4GlcNAcβ1-3Galβ1-4GlcNAcβ1-2Manα1-3)Manβ1-4GlcNAcβ1-4(Fucα1-6)GlcNAcβ-Sp24

**567**	Galβ1-4GlcNAcβ1-3Galβ1-4GlcNAcβ1-3Galβ1-4GlcNAcβ1-2Manα1-6(Galβ1-4GlcNAcβ1-3Galβ1-4GlcNAcβ1-3Galβ1-4GlcNAcβ1-2Manα1-3)Manβ1-4GlcNAcβ1-4(Fucα1-6)GlcNAcβ-Sp24
**568**	GlcNAcβ1-3Galβ1-4GlcNAcβ1-3Galβ1-4GlcNAcβ1-3Galβ1-4GlcNAcβ1-2Manα1-6(GlcNAcβ1-3Galβ1-4GlcNAcβ1-3Galβ1-4GlcNAcβ1-3Galβ1-4GlcNAcβ1-2Manα1-3)Manβ1-4GlcNAcβ1-4(Fucα1-6)GlcNAcβ-Sp24
**569**	Galβ1-4GlcNAcβ1-3Galβ1-4GlcNAcβ1-3Galβ1-4GlcNAcβ1-3Galβ1-4GlcNAcβ1-2Manα1-6(Galβ1-4GlcNAcβ1-3Galβ1-4GlcNAcβ1-3Galβ1-4GlcNAcβ1-3Galβ1-4GlcNAcβ1-2Manα1-3)Manβ1-4GlcNAcβ1-4(Fucα1-6)GlcNAcβ-Sp24
**570**	GlcNAcβ1-3Galβ1-4GlcNAcβ1-3Galβ1-4GlcNAcβ1-3Galβ1-4GlcNAcβ1-3Galβ1-4GlcNAcβ1-2Manα1-6(GlcNAcβ1-3Galβ1-4GlcNAcβ1-3Galβ1-4GlcNAcβ1-3Galβ1-4GlcNAcβ1-3Galβ1-4GlcNAcβ1-2Manα1-3)Manβ1-4GlcNAcβ1-4(Fucα1-6)GlcNAcβ-Sp19
**571**	Galβ1-4GlcNAcβ1-3Galβ1-4GlcNAcβ1-3Galβ1-4GlcNAcβ1-3Galβ1-4GlcNAcβ1-3Galβ1-4GlcNAcβ1-2Manα1-6(Galβ1-4GlcNAcβ1-3Galβ1-4GlcNAcβ1-3Galβ1-4GlcNAcβ1-3Galβ1-4GlcNAcβ1-3Galβ1-4GlcNAcβ1-2Manα1-3)Manβ1-4GlcNAcβ1-4(Fucα1-6)GlcNAcβ-Sp19
**572**	Galβ1-4GlcNAcβ1-3Galβ1-4GlcNAcβ1-6(Galβ1-4GlcNAcβ1-3Galβ1-4GlcNAβ1-2)Manα1-6(Galβ1-4GlcNAcβ1-3Galβ1-4GlcNAcβ1-2Manα1-3)Manβ1-4GlcNAcβ1-4(Fucα1-6)GlcNAcβ-Sp24
**573**	GlcNAcβ1-3Galβ1-4GlcNAcβ1-3Galβ1-4GlcNAcβ1-6(GlcNAcβ1-3Galβ1-4GlcNAcβ1-3Galβ1-4GlcNAβ1-2)Manα1-6(GlcNAcβ1-3Galβ1-4GlcNAcβ1-3Galβ1-4GlcNAcβ1-2Manα1-3)Manβ1-4GlcNAcβ1-4(Fucα1-6)GlcNAcβ-Sp24
**574**	Galβ1-4GlcNAcβ1-3Galβ1-4GlcNAcβ1-3Galβ1-4GlcNAcβ1-6(Galβ1-4GlcNAcβ1-3Galβ1-4GlcNAcβ1-3Galβ1-4GlcNAβ1-2)Manα1-6(Galβ1-4GlcNAcβ1-3Galβ1-4GlcNAcβ1-3Galβ1-4GlcNAcβ1-2Manα1-3)Manβ1-4GlcNAcβ1-4(Fucα1-6)GlcNAcβ-Sp24

**575**	GlcNAcβ1-3Galβ1-4GlcNAcβ1-3Galβ1-4GlcNAcβ1-3Galβ1-4GlcNAcβ1-6(GlcNAcβ1-3Galβ1-4GlcNAcβ1-3Galβ1-4GlcNAcβ1-3Galβ1-4GlcNAβ1-2)Manα1-6(GlcNAcβ1-3Galβ1-4GlcNAcβ1-3Galβ1-4GlcNAcβ1-3Galβ1-4GlcNAcβ1-2Manα1-3)Manβ1-4GlcNAcβ1-4(Fucα1-6)GlcNAcβ-Sp24
**576**	Galβ1-4GlcNAcβ1-3Galβ1-4GlcNAcβ1-3Galβ1-4GlcNAcβ1-3Galβ1-4GlcNAcβ1-6(Galβ1-4GlcNAcβ1-3Galβ1-4GlcNAcβ1-3Galβ1-4GlcNAcβ1-3Galβ1-4GlcNAβ1-2)Manα1-6(Galβ1-4GlcNAcβ1-3Galβ1-4GlcNAcβ1-3Galβ1-4GlcNAcβ1-3Galβ1-4GlcNAcβ1-2Manα1-3)Manβ1-4GlcNAcβ1-4(Fucα1-6)GlcNAcβ-Sp24
**577**	GlcNAcβ1-3Galβ1-4GlcNAcβ1-3Galβ1-4GlcNAcβ1-3Galβ1-4GlcNAcβ1-3Galβ1-4GlcNAcβ1-6(GlcNAcβ1-3Galβ1-4GlcNAcβ1-3Galβ1-4GlcNAcβ1-3Galβ1-4GlcNAcβ1-3Galβ1-4GlcNAβ1-2)Manα1-6(GlcNAcβ1-3Galβ1-4GlcNAcβ1-3Galβ1-4GlcNAcβ1-3Galβ1-4GlcNAcβ1-3Galβ1-4GlcNAcβ1-2Manα1-3)Manβ1-4GlcNAcβ1-4(Fucα1-6)GlcNAcβ-Sp24
**578**	Galβ1-4GlcNAcβ1-3Galβ1-4GlcNAcβ1-3Galβ1-4GlcNAcβ1-3Galβ1-4GlcNAcβ1-3Galβ1-4GlcNAcβ1-6(Galβ1-4GlcNAcβ1-3Galβ1-4GlcNAcβ1-3Galβ1-4GlcNAcβ1-3Galβ1-4GlcNAcβ1-3Galβ1-4GlcNAβ1-2)Manα1-6(Galβ1-4GlcNAcβ1-3Galβ1-4GlcNAcβ1-3Galβ1-4GlcNAcβ1-3Galβ1-4GlcNAcβ1-3Galβ1-4GlcNAcβ1-2Manα1-3)Manβ1-4GlcNAcβ1-4(Fucα1-6)GlcNAcβ-Sp24
**579**	Galβ1-4GlcNAcβ1-3Galβ1-4GlcNAcβ1-3GalNAcα-Sp14
**580**	Galβ1-4GlcNAcβ1-3Galβ1-4GlcNAcβ1-6(Galβ1-3)GalNAcα-Sp14
**581**	Galβ1-4GlcNAcβ1-3Galβ1-4GlcNAcβ1-6(Galβ1-4GlcNAcβ1-3Galβ1-4GlcNAcβ1-3)GalNAcα-Sp14
**582**	Neu5Acα2-3Galβ1-4GlcNAcβ1-3Galβ1-4GlcNAcβ1-3GalNAcα-Sp14
**583**	GlcNAcβ1-3Galβ1-4GlcNAcβ1-3GalNAcα-Sp14
**584**	GlcNAcβ1-3Galβ1-4GlcNAcβ1-6(Galβ1-3)GalNAcα-Sp14
**585**	GlcNAcβ1-3Galβ1-4GlcNAcβ1-6(GlcNAcβ1-3Galβ1-4GlcNAcβ1-

	3)GalNAcα-Sp14
**586**	Neu5Acα2-3Galβ1-4GlcNAcβ1-3Galβ1-4GlcNAcβ1-6(Neu5Acα2-3Galβ1-4GlcNAcβ1-3Galβ1-4GlcNAcβ1-3)GalNAcα-Sp14
**587**	Neu5Acα2-6Galβ1-4GlcNAcβ1-3Galβ1-4GlcNAcβ1-3GalNAcα-Sp14
**588**	GlcNAcβ1-3Galβ1-4GlcNAcβ1-3Galβ1-4GlcNAcβ1-3GalNAcα-Sp14
**589**	Galβ1-4GlcNAcβ1-3Galβ1-3GalNAcα-Sp14
**590**	Neu5Acα2-3Galβ1-4GlcNAcβ1-3Galβ1-4GlcNAcβ1-6(Galβ1-3)GalNAcα-Sp14
**591**	Neu5Acα2-6Galβ1-4GlcNAcβ1-3Galβ1-4GlcNAcβ1-6(Galβ1-3)GalNAcα-Sp14
**592**	Neu5Acα2-6Galβ1-4GlcNAcβ1-6(Galβ1-3)GalNAcα-Sp14
**593**	Neu5Acα2-3Galβ1-4GlcNAcβ1-3Galβ1-4GlcNAcβ1-2Manα1-6(Neu5Acα2-3Galβ1-4GlcNAcβ1-3Galβ1-4GlcNAcβ1-2Manα1-3)Manβ1-4GlcNAcβ1-4GlcNAcβ-Sp12
**594**	GlcNAcβ1-6(Neu5Acα2-3Galβ1-3)GalNAcα-Sp14
**595**	Neu5Acα2-6Galβ1-4GlcNAcβ1-3Galβ1-4GlcNAcβ1-6(Neu5Acα2-6Galβ1-4GlcNAcβ1-3Galβ1-4GlcNAcβ1-3)GalNAcα-Sp14
**596**	Neu5Acα2-6Galβ1-4GlcNAcβ1-3Galβ1-4GlcNAcβ1-3Galβ1-4GlcNAcβ1-2Manα1-6(Neu5Acα2-6Galβ1-4GlcNAcβ1-3Galβ1-4GlcNAcβ1-3Galβ1-4GlcNAcβ1-2Manα1-3)Manβ1-4GlcNAcβ1-4GlcNAcβ-Sp12
**597**	Neu5Acα2-3Galβ1-4GlcNAcβ1-3Galβ1-4GlcNAcβ1-3Galβ1-4GlcNAcβ1-2Manα1-6(Neu5Acα2-3Galβ1-4GlcNAcβ1-3Galβ1-4GlcNAcβ1-3Galβ1-4GlcNAcβ1-2Manα1-3)Manβ1-4GlcNAcβ1-4GlcNAcβ-Sp12
**598**	Neu5Acα2-6Galβ1-4GlcNAcβ1-3Galβ1-4GlcNAcβ1-2Manα1-6(Neu5Acα2-6Galβ1-4GlcNAcβ1-3Galβ1-4GlcNAcβ1-2Manα1-3)Manβ1-4GlcNAcβ1-4GlcNAcβ-Sp12

**599**	GlcNAcβ1-3Fucα-Sp21
**600**	Galβ1-3GalNAcβ1-4(Neu5Acα2-8Neu5Acα2-8Neu5Acα2-3)Galβ1-4Glcβ-Sp21

Sp0= $-CH_2CH_2NH_2$; Sp8= $-CH_2CH_2CH_2NH_2$; Sp9= $-CH_2CH_2CH_2CH_2CH_2NH_2$; Sp10= $-NHCOCH_2NH$; Sp11= $-OCH_2C_6H_4$-p-$NHCOCH_2NH$; Sp12= Asparagine; Sp13= Glycine; Oligomannose-type glycosides and partial structures thereof are highlighted in yellow.

Sp14= Threonine; Sp15= Serine; Sp16= $-PNP(OC_6H_4NH_2)$; Sp17= $OCH_2C_6H_4NH_2$; Sp18= $-O(CH_2)_3NHCO(CH_2)_5NH_2$; Sp19= EN or NK; Sp20= GENR; Sp21= $-N(CH_3)-O-(CH_2)_2-NH_2$; MDPLys= Mur-L-Ala-D-iGlnβ-$(CH_2)_4NH_2$

Supplementary Table 2. Serum antibody binding to arrayed glycans, excluding oligomannose-type glycans and related partial structures

Serum 13665		Serum 13667		Serum 13668	
Glycan ID	Fold difference in binding	Glycan ID	Fold difference in binding	Glycan ID	Fold difference in binding
585	2.4	188	4.2	190	9.7
419	0.1	190	4.9	191	6.6
575	2.8	539	0.04	308	5.5
177	0.1	196	19.2	198	4.9
577	2.6	177	0.04	568	3.4
281	2.0	309	24.1	177	0.1
595	1.9	561	0.01	335	0.2
488	4.6	339	2.1	310	0.03
441	2.4	338	0.03	309	6.9
568	3.6	97	0.2	566	6.1
188	5.4	408	0.02	555	7.0
520	2.8	511	30.1	149	0.3
442	4.2	402	21.8	189	9.0
416	3.5	465	0.05	496	0.3
189	0.3	194	3.0	338	0.4
204	4.5	172	0.2	538	2.4
538	2.7	581	0.1	514	0.3
341	2.4	381	5.0	482	0.3
493	2.1	321	0.1	541	0.3
357	2.7	340	0.2	186	0.6
380	4.1	191	3.0	235	0.3
176	0.2	219	3.1	586	1.9
358	2.4	534	8.4	180	0.7
375	2.6	575	0.5	362	2.1
561	2.0	514	0.2	251	1.9
501	2.3	598	0.1	165	3.3
562	1.9	94	0.2	424	2.4
454	2.2	562	0.1	148	3.4

459	2.6	429	0.1	274	0.5
382	8.2	580	0.03	219	0.2
424	2.4	199	38.0		
425	2.5	308	7.7		
107	2.5	420	2.4		
384	15.4	577	0.4		
198	1.9	482	3.5		
573	3.1	568	0.3		
583	2.3	496	0.5		
392	2.0	440	0.2		
579	1.7	549	0.1		
447	2.1	554	0.1		
391	2.2	510	8.6		
383	5.1	66	0.4		
515	6.3	112	0.4		
592	3.7	499	0.3		
174	3.5				
356	2.9				
250	3.4				
362	4.2				
303	2.2				
482	5.8				
197	7.0				
514	0.5				
445	3.1				
408	3.7				
456	2.5				
599	2.2				
487	3.3				
593	3.6				
381	8.5				
460	4.0				
352	1.8				

587	4.8				
428	3.1				
186	0.7				
219	2.4				
457	2.9				
339	2.4				
455	3.4				
136	2.2				
534	6.0				
502	3.4				
366	37.4				
340	3.9				
102	4.0				
586	2.9				
581	1.9				
528	2.9				
172	1.7				
378	2.9				
409	2.7				
372	1.5				
64	2.2				
451	3.5				
597	2.9				
423	2.6				
465	0.3				

The chemical sequences of the arrayed glycosides are listed in **Supplementary Table 1**. Shown is the fold difference in serum antibody binding relative to the pre-immune control. To facilitate visualization, a color gradient was applied so that the warmer the color (blue→red), the greater the difference in serum binding relative to control.

Supplementary Table 3. Hydrogen bonds in PGT128 complex with Man_9 vs NIT68A

PGT128 atom	Man_9 atom	Man_9 distance (3TV3) (Å)	NIT68A distance (Å)
Thr^{H57}-N	Man^{C}-O4	3.09	3.01
His^{H59}-Nδ1	Man^{D1}-O2	2.71	2.60
His^{H59}-N	Man^{D1}-O3	2.90	2.92
Lys^{H64}-Nζ	Man^{D1}-O2	3.15	3.11
Lys^{H64}-Nζ	Man^{D1}-O5	3.01	2.90
Trp^{H95}-Nε1	Man^{D1}-O4	2.97	2.93
Asn^{L94}- Nδ2	$Man^{4'}$-O5	3.03	3.15
Asn^{L94}- N	Man^{D3}-O5	3.19	3.38
Asn^{L94}- N	Man^{D3}-O6	3.06	2.95
Trp^{L95}- N	Man^{D3}-O6	2.94	2.88

Supplementary Table 4. Data collection and refinement statistics

	PGT128 Fab - NIT68A (6B3D)
**Data collection**	
Space group	$C222_1$
Cell dimensions [*a*, *b*, *c* (Å)]	72.1 105.2 145.3
Resolution (Å)	50.0-2.27 (2.35-2.27)*
No. of unique reflections	25,845
R_{sym}	10.2 (35.6)
R_{pim}	2.9 (12.2)
$I/\sigma(I)$	25.7 (6.6)
$CC_{1/2}$	0.99 (0.96)
Completeness (%)	99.8 (96.6)
Redundancy	11.8 (8.0)
**Refinement**	
Resolution (Å)	36.3-2.27
No. reflections	25,807
R_{work} / R_{free}	0.163 / 0.198
No. atoms	
Protein (PGT128)	3326
Ligand (NIT68A)	78
Solvent	69
Waters	152
Wilson *B* (Å^2)	33
B values (Å^2)	
Protein (PGT128)	36
Ligand (NIT68A)	50
Solvent	57
Waters	41
R.m.s. deviations	
Bond lengths (Å)	0.003
Bond angles (°)	0.67
Ramachandran statistics	
Preferred regions (%)	97.5
Outliers (%)	0.0

* Values in parentheses are for highest-resolution shell.

SUPPLEMENTARY METHODS

Nuclear Magnetic Resonance (NMR).

For the branched oligomers, the following labeling of residues was used for NMR assignments:

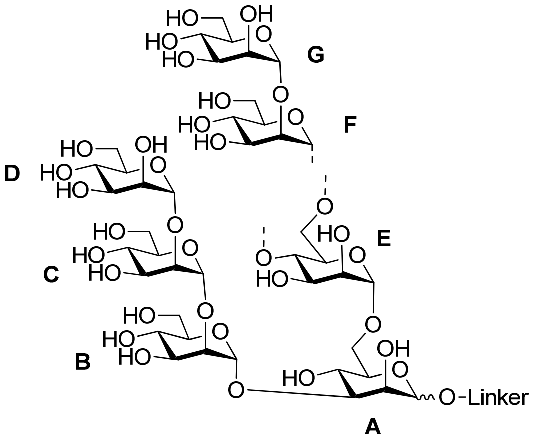

Synthesis of donors and acceptors.

4-Methylphenyl 2-*O*-benzoyl-3-*O*-benzyl-4,6-*O*-benzylidene-1-thio-α-D-mannopyranoside (4)

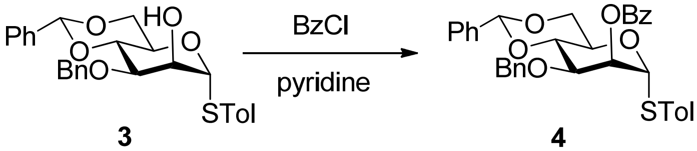

3 4

Benzoyl chloride (0.6 ml, 5.17 mmol; 1.5 eq.) was added dropwise to a solution of **3**[1,2] (1.6 g; 3.44 mmol) in dry pyridine (30 ml) under argon (Ar) and stirred for 3 h at RT. A solution of aq. satd. $NaHCO_3$ (30 ml) and DCM (30 ml) were added and the phases were separated. The aqueous phase was extracted with DCM (2×30 ml), the combined organic phases were dried (Na_2SO_4) and the solvent was removed *in vacuo*. The product was purified by silica flash chromatography (hexane/EtOAc = 10/1) to give **4** (1.778 g; 94 %) as a colorless oil; $[\alpha]_D^{21}$ +60.8 (*c* 1.1, $CHCl_3$). ^{1}H NMR (600 MHz, $CDCl_3$): δ = 8.13-8.10 (m, 2 H, Ar), 7.61-7.24 (m, 15 H, Ar), 7.16-7.13 (m, 2 H, Ar), 5.86 (dd, *J* = 3.4, 1.6 Hz, 1 H, H-2), 5.72 [s, 1 H, $ArCH(OR)_2$], 5.56 (d, *J* = 1.6 Hz, 1 H, H-1), 4.79 (d, *J* = 12.3 Hz, 1 H, CH_2Ar), 4.75 (d, *J* = 12.3 Hz, 1 H, CH_2Ar), 4.47 (ddd, *J* = 10.3, 9.7 and 4.7 Hz, 1 H, H-5), 4.30 (dd, *J* = 10.0, 4.7 Hz, 1 H, H-6a), 4.29 (t, *J* = 10.3 Hz, 1 H, H-4), 4.16 (dd, *J* = 10.0, 3.4 Hz, 1 H, H-3), 3.93 (dd, *J* = 10.3, 9.7 Hz, 1 H, H-6_b), 2.34 (s, 3 H, CH_3ArS). ^{13}C NMR (150 MHz, $CDCl_3$): δ = 165.9 (COAr), 138.3, 137.7, 137.4, 134.5, 133.3, 132.7, 130.5, 130.0, 129.9, 129.7, 129.2, 128.9, 128.8, 128.4, 128.3, 128.2, 127.6, 126.1 (24 C,

Ar), 101.6 [ArCH(OR)$_2$], 87.5 (C-1), 78.9 (C-4), 74.2 (C-3), 72.1 (CH$_2$Ar), 71.9 (C-2), 68.5 (C-6), 65.1 (C-5), 21.1 (CH$_3$ArS). ESI-TOF HRMS: *m/z* calcd for $C_{34}H_{32}O_6S$ [M+Na$^+$]$^+$: 591.1812; found: 591.1826.

2-*O*-Benzoyl-3-*O*-benzyl-4,6-*O*-benzylidene-D-mannopyranose-2,2,2-trichloroacetimidate (5)

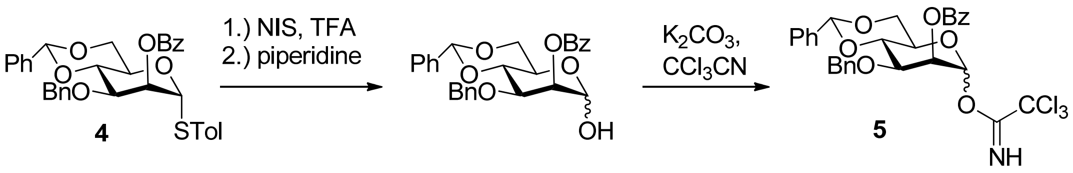

A solution of **4** (5.00 g; 8.79 mmol) in dry DCM (100 ml) was cooled to 0 °C and NIS (2.57 g; 11.43 mmol) was added under Ar, followed by addition of TFA (0.88 ml; 11.43 mmol). The reaction mixture was allowed to warm to RT and was stirred for 1 h, before piperidine (3.4 ml; 34.29 mmol) was added and stirring was continued for 30 min. Triethylamine was then added and the organic Phase was washed with aq 5 % $Na_2S_2O_3$, dried (Na_2SO_4) and the solvent was removed *in vacuo*. The crude product was purified *via* silica gel flash chromatography (hexane/EtOAc = 4/1) to give the intermediate hemiacetal. The residue was dissolved in dry DCM (25 ml) under argon. Solid K_2CO_3 (3.59 g; 25.95 mmol) was added at RT and the suspension was stirred for 10 min followed by addition of CCl_3CN (3.47 ml; 34.60 mmol). The suspension was stirred for 5 h at RT and was then filtered over Celite®. The solvent was removed *in vacuo* and the crude reaction product was purified *via* silica flash chromatography (hexane/EtOAc = 10/1 → 5/1), to afford **5** (4.53 g; 86%) as colorless amorphous solid; $[\alpha]_D^{21}$ -9.9 (*c* 1.0, CHCl$_3$).^{1}H NMR (600 MHz, CDCl$_3$): δ = 8.74 (s, 1 H, NH), 8.14-8.10 (m, 2 H, Ar), 7.62-7.21 (m, 13 H, Ar), 6.37 (d, *J* = 1.8 Hz, 1 H, H-1), 5.71 (dd, *J* = 3.5, 1.8 Hz, 1 H, H-2), 5.71 [s, 1 H, ArCH(OR$_2$)], 4.79 (d, *J* = 12.1 Hz, 1 H, OCH$_2$Ar), 4.75 (d, *J* = 12.1 Hz, 1 H, OCH$_2$Ar), 4.36 (dd, *J* = 10.4, 4.9 Hz, 1 H, H-6a), 4.29 (t, *J* = 9.6 Hz, 1 H, H-4), 4.20 (dd, *J* = 10.2, 3.6 Hz, 1 H, H-3), 4.09 (ddd, *J* = 10.4, 10.2 and 4.9 Hz, 1 H, H-5), 3.92 (t, *J* = 10.4 Hz, 1 H, H-6b); ^{13}C NMR (150 MHz, CDCl$_3$): δ = 165.4 (C=O), 160.0 (C=NH), 137.6, 137.2, 133.5, 130.0, 129.4, 129.0, 128.5, 128.3, 128.2, 127.9, 127.8, 126.0 (19 C, Ar, CCl$_3$), 101.7 (C-1), 95.7 [ArCH(OR$_2$)], 78.2 (C-4), 73.3 (C-3), 72.5 (O*C*H$_2$Ar), 69.0 (C-2), 68.5 (C-6), 66.5 (C-5). ESI-TOF HRMS: *m/z* calcd for $C_{29}H_{53}O_{14}S$ [M – O(NH)CCCl$_3$ + H$^+$]$^+$: 445.1646; found 445.1649.

4-Methylphenyl (2-*O*-acetyl-3,4,6-tri-*O*-benzoyl-D-mannopyranosyl)-(1→3)-2-*O*-benzyl-4,6-*O*-benzylidene-1-thio-α-D-mannopyranoside (7)

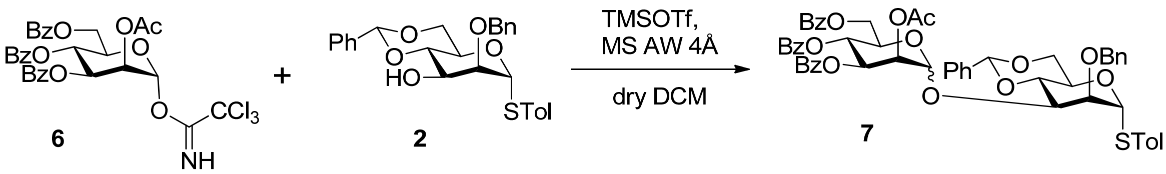

Acid-washed molecular sieves 4 Å (0.90 g) was added to a solution of **2**[1,2] (0.9 g; 1.94 mmol) and **6**[3-6] (1.71 g; 2.52 mmol; 1.3 eq) in dry DCM (15 ml) under Ar. The suspension was stirred for 10 min before TMSOTf (0.035 ml; 0.19 mmol; 0.1 eq.) was added dropwise. The reaction mixture was stirred at RT for 20 min and was subsequently quenched by addition of a few drops of NEt_3. The suspension was filtered over a plug of Celite® and the solvent was removed *in vacuo*. The crude product was purified *via* silica flash chromatography (toluene/EtOAc = 30/1) to give **7** (1.67 g; 88%) as a colorless oil. The product is an inseparable mixture of α- and β-product (ratio 1:0.08) and was used as such in the subsequent reaction. ^{1}H NMR (600 MHz, $CDCl_3$): δ = 8.06-8.04 (m, 2 H, Ar), 7.94-7.89 (m, 4 H, Ar), 7.54-7.25 (m, 21 H, Ar), 7.15-7.12 (m, 2 H, Ar), 5.91 (dd, *J* = 10.1 Hz, 3.4 Hz, 1 H, H-3'), 5.84 (t, *J* = 10.0 Hz, 1 H, H-4'), 5.72 (dd, *J* = 3.4, 1.9 Hz, 1 H, H-2'), 5.66 [s, 1 H, ArCH$(OR)_2$], 5.53 (d, *J* = 1.3 Hz, 1 H, H-1), 5.44 (d, *J* = 1.9 Hz, 1 H, H-1'), 4.89 (d, *J* = 12.3 Hz, 1 H, OCH_2Ar), 4.77 (d, *J* = 12.3 Hz, 1 H, OCH_2Ar), 4.54 (dd, *J* = 12.1, 2.8 Hz, 1 H, H-6'a), 4.42-4.38 (m, 2 H, H-6'b, H-4), 4.36-4.30 (m, 2 H, H-3, H-4), 4.25 (dd, *J* = 10.3, 4.6 Hz, 1 H, H-6a), 4.24-4.20 (m, 1 H, H-5'), 4.14 (dd, *J* = 3.1, 1.3 Hz, 1 H, H-2) 3.90 (t, *J* = 10.3 Hz, 1 H, H-6b) 2.36 (s, 3 H, CH_3Ar), 2.10 (s, 3 H, CH_3CO). ^{13}C NMR (150 MHz, $CDCl_3$): δ = 169.2 (CH_3C=O), 166.1, 165.5, 165.3 (ArC=O), 138.0, 137.4, 137.2, 133.4, 133.2, 133.0, 132.2, 129.9, 129.8, 129.7, 129.6, 129.2, 128.7, 128.4, 128.3, 128.0, 127.9, 125.9 (36 C, Ar), 101.2 [ArCH$(OR)_2$], 98.7 (C-1'), 86.8 (C-1), 78.9 (C-4), 78.8 (C-2), 74.2 (C-3), 72.6 (OCH_2Ar), 69.5 (2 C, C-5', C-2'), 69.4 (C-3'), 68.3 (C-6), 67.3 (C-4'), 65.1 (C-5), 63.5 (C-6'). ESI-TOF HRMS: *m/z* calcd for $C_{56}H_{52}O_{14}S$ [M+H$^+$]$^+$: 981.3151; found: 981.3155.

4-Methylphenyl 3,4,6-tri-*O*-benzoyl-α-D-mannopyranosyl-(1→3)-2-*O*-benzyl-4,6-*O*-benzylidene-1-thio-α-D-mannopyranoside (8)

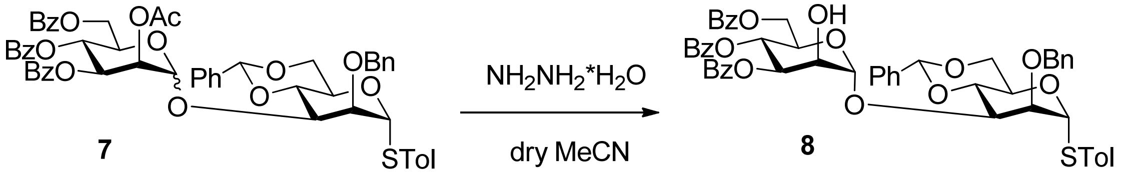

Hydrazine monohydrate (0.03 ml; 0.612 mmol; 4 eq.) was added to a stirred solution of **7** (α/β = 1/0.8; 0.15 g; 0.153 mmol) in dry MeCN (3 ml) under Ar and the reaction mixture was stirred at RT for 40 h. The solvent was evaporated and the crude product was purified by silica column chromatography (toluene/EtOAc = 30/1), which afforded the α-anomer **8** (75 mg; 52 %) as colorless amorphous solid as well as a fraction of the anomeric substrate mixture (45 mg; 30 %); $[\alpha]_D^{21}$ +60.5 (*c* 1.0, $CHCl_3$). ^1H NMR (600 MHz, $CDCl_3$): δ = 8.01-7.82 (m, 6 H, Ar), 7.50-7.11 (m, 23 H, Ar), 5.91 (t, *J* = 10.0 Hz, 1 H, H-4'), 5.82 (dd, *J* = 10.0, 3.2 Hz, 1 H, H-3'), 5.64 [s, 1 H, $ArCH(OR)_2$], 5.54 (d, *J* = 1.3 Hz, 1 H, H-1), 5.45 (d, *J* = 1.7 Hz, 1 H, H-1'), 4.89 (d, *J* = 12.2 Hz, 1 H, OCH_2Ar), 4.76 (d, *J* = 12.2 Hz, 1 H, OCH_2Ar), 4.54 (dd, *J* = 12.3, 3.0 Hz, 1 H, H-6a), 4.46-4.43 (m, 1 H, H-2'), 4.42 (dd, *J* = 12.3, 5.6 Hz, 1 H, H-6b), 4.38-4.33 (m, 3 H, H-3, H-4, H-5), 4.28-4.22 (m, 2 H, H-5', H-6'a), 4.17-4.16 (broad signal, 1 H, H-2), 3.92-3.87 (m, 1 H, H-6'b), 2.36 (s, 3 H, $SArCH_3$). ^{13}C NMR (150 MHz, $CDCl_3$): δ = 166.3, 165.5 (3 C, ArC=O), 138.1, 137.4, 137.3, 133.3, 133.0, 132.3, 129.9, 129.8, 129.7, 129.3, 129.1, 129.0, 128.6, 128.3, 128.4, 128.3, 128.2, 128.9, 126.0 (36 C, Ar), 101.6 [$ArCH(OR)_2$], 100.9 (C-1'), 86.7 (C-1), 79.1 (C-2), 78.9 (C-4), 74.6 (C-3), 72.6 (OCH_2Ar), 72.1 (C-3'), 69.4 (2 C, C-2', C-5'), 68.5 (C-6'), 67.2 (C-4'), 65.1 (C-5), 63.7 (C-6), 21.1 (CH_3ArS). ESI-TOF HRMS: *m/z* calcd for $C_{54}H_{50}O_{13}S$ $[M+H^+]^+$: 956.3310, found: 956.3355.

2-*O*-Acetyl-3,4,6-tri-*O*-benzoyl-α-D-mannopyranosyl-(1→2)-3,4,6-tri-*O*-benzoyl-D-mannopyranose 2,2,2-trichloroacetimidate (11)

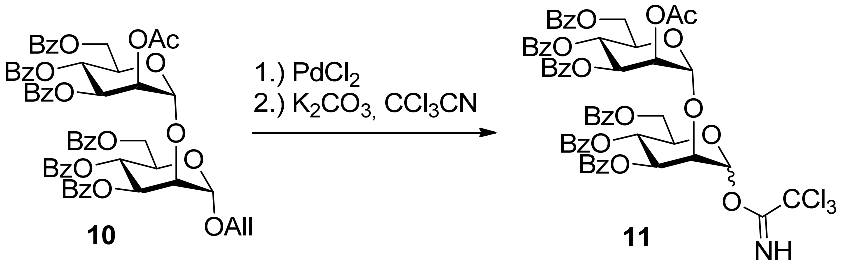

$PdCl_2$ (20 mg; 0.11 mmol; 0.1 eq.) was added to a solution of **10**[3,7,8] (1.19 g; 1.13 mmol) in dry MeOH (10 ml) and dry DCM (10 ml) under argon. The suspension was stirred at RT for 7 h, then filtered over a pad of Celite® and the solvent was removed *in vacuo*. The crude intermediate was dissolved in dry DCM (10 ml) under Ar, and solid K_2CO_3 (0.47 g; 3.40 mmol) was added. The suspension was stirred for 10 min at RT before CCl_3CN (0.6 ml; 4.54 mmol) was added dropwise and stirring was continued at RT until full conversion (16 h). The mixture was filtered over a pad of Celite® and the filtrate was concentrated. The crude product was purified by silica flash chromatography (toluene/EtOAc = 30/1) to give **11** (0.82 g; 63 %) as colorless syrup. ^{1}H NMR (600 MHz, $CDCl_3$): δ = 8.64 (s, 1 H, NH), 8.01-7.91 (m, 12 H, Ar), 7.53-7.31 (m, 18 H, Ar), 6.61 (d, *J* = 2.4 Hz, 1 H, H-1), 6.10 (t, *J* = 9.9 Hz, 1 H, H-4), 5.94-5.88 (m, 3 H, H-3, H-3', H-4'), 5.70 (dd, *J* = 2.9, 2.0 Hz, 1 H, H-2'), 5.20 (d, *J* = 2.0, 1 H, H-1'), 4.67 (dd, *J* = 12.1, 2.4 Hz, 1 H, H-6a), 4.65-4.55 (m, 4 H, H-2, H-5, H-5', H-6'a), 4.55 (dd, *J* = 11.2, 4.7 Hz, 1 H, H-6b), 4.50 (dd, *J* = 11.2, 5.5 Hz, 1 H, H-6'b), 2.05 (s, 3 H, CH_3CO). ^{13}C NMR (150 MHz, $CDCl_3$): δ = 169.2 (CH_3CO), 166.2 (ArCO), 166.1 (ArCO), 165.5 (ArCO), 165.1 (ArCO), 165.0 (ArCO), 159.9 (C=NH), 133.5, 133.4, 133.2, 133.0, 130.0, 129.9, 129.8, 129.7, 129.6, 128.8, 128.7, 128.6, 128.4, 128.3 (37 C, Ar, CCl_3), 99.3 (C-1'), 96.2 (C-1), 74.1 (C-5), 71.7 (C-5'), 70.5 (C-3), 70.1 (C-3'), 69.5 (C-2), 69.4 (C-2'), 66.8 (C-4'), 66.7 (C-4), 63.1 (C-6'), 63. (C-6), 20.5 ((CH_3CO).

4-Methylphenyl 2-*O*-acetyl-3,4,6-tri-*O*-benzoyl-α-D-mannopyranosyl-(1→2)-3,4,6-tri-*O*-benzoyl-α-D-mannopyranosyl-(1→2)-3,4,6-tri-*O*-benzoyl-α-D-mannopyranosyl-(1→3)-2-*O*-benzyl-4,6-*O*-benzylidene-1-thio-α-D-mannopyranoside (13)

A) 2+2 Coupling

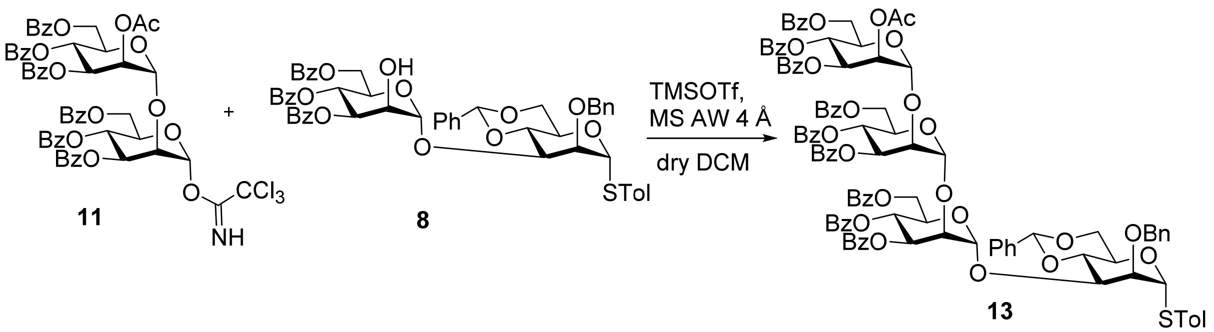

A suspension of **8** (0.82 g; 0.87 mmol), **11** (1.30 g; 1.13 mmol; 1.3 eq) and powdered acid-washed molecular sieves 4 Å (2 g) in dry DCM (10 ml) was stirred under Ar for 20 min at RT.

Then TMSOTf (0.015 ml; 0.09 mmol; 0.1 eq) was added dropwise and stirring was continued until complete consumption of the acceptor **8** (1 h). The reaction was quenched by the addition of 3 drops of NEt_3 and the mixture was filtered over a pad of Celite®. The filtrate was concentrated and the crude product was purified *via* silica flash chromatography (hexane/EtOAc = 2.5/1 → 1/1) to give tetrasaccharide **13** (1.51 g; 90 %) as colorless syrup; $[\alpha]_D^{21}$ +60.1 (*c* 1.1, $CHCl_3$). ^1H NMR (600 MHz, $CDCl_3$): δ = 8.12-8.01 (m, 6 H, Ar), 7.97-7.84 (m, 10 H, Ar), 7.79-7.75 (m, 2 H, Ar), 7.57-7.21 (m, 36 H, Ar), 7.16-7.10 (m, 4 H, Ar), 6.81-6.79 (m, 1 H, Ar), 5.99-5.89 (m, 4 H, H-3, H-3', H-4, H-4'), 5.82 (dd, *J* = 10.0, 3.4 Hz, 1 H, H-3''), 5.75 (t, *J* = 10.0 Hz, 1 H, H-4'''), 5.71 (d, *J* = 2.0 Hz, 1 H, H-1'), 5.61 (dd, *J* = 3.0, 2.2 Hz, 1 H, H-2'''), 5.52 (d, *J* = 1.0 Hz, 1 H, H-1), 5.42 (d, *J* = 1.8 Hz, 1 H, H-1''), 5.26 [s, 1 H, $CH(OR)_2Ar$], 4.94 (d, *J* = 12.0 Hz, 1 H, OCH_2Ar), 4.92 (s, 1 H, H-1'''), 4.91 (d, *J* = 12.0 Hz, 1 H, OCH_2Ar), 4.64 (dd, *J* = 12.3, 3.0 Hz, 1 H, H-6a''), 4.57 (dd, *J* = 3.0, 2.3 Hz, 1 H, H-2'), 4.53 (dd, *J* = 12.4, 6.5 Hz, 1 H, H-6b''), 4.48-4.45 (m, 2 H, H-3, H-2''), 4.38-4.24 (m, 5 H, H-5'', H-4, H-5''', H-5, H-5'), 4.18 (dd, *J* = 3.2, 1.2 Hz, 1 H, H-2), 4.16 (dd, *J* = 10.4, 4.9 Hz, 1 H, H-6a), 4.15-4.10 (m, 1 H, H-6a'''), 4.07 (dd, *J* = 12.6, 5.4 Hz, 1 H, H-6a'), 4.00 (dd, *J* = 12.4, 5.0 Hz, 1 H, H-6b'''), 3.95 (dd, *J* = 12.6, 2.3 Hz, 1 H, H-6b'), 3.76 (t, *J* = 10.4 Hz, 1 H, H-6b), 2.35 (s, 3 H, CH_3ArS), 2.03 (s, 3 H, CH_3CO). ^{13}C NMR (150 MHz, $CDCl_3$): δ = 169.0 (CH_3CO), 166.4, 166.0, 165.7, 165.6, 165.5, 165.4, 165.3, 165.2, 164.9 (9 C, ArCO), 138.2-126.0 (72 C, Ar), 101.6 [$CH(OR)_2Ar$], 99.8 (2 C, C1'', C-1'''), 99.3 (C-1'), 87.2 (C-1), 79.2 (2 C, C-2, C-5''), 77.5 (C-2''), 76.0 (C-2'), 73.2 (OCH_2Ar), 72.9 (C-3), 70.8 (C-3'), 70.5 (C-3''), 69.7 (C-2'''), 69.6 (C-3'''), 69.5 (2 C, C-5''', C-5), 69.4 (C-4), 68.4 (C-6), 67.7 (C-4''), 66.9 (2 C, C-4', C-4'''), 65.0 (C-5'), 64.0 (C-6''), 63.2 (C-6'), 63.0 (C-6'''), 21.1 (CH_3ArS), 20.6 (CH_3CO). ESI-TOF HRMS: *m/z* calcd for $C_{110}H_{96}O_{30}S$ $[M+H^+]^+$: 1946.6045; found: 1946.6079.

B) 3+1 coupling

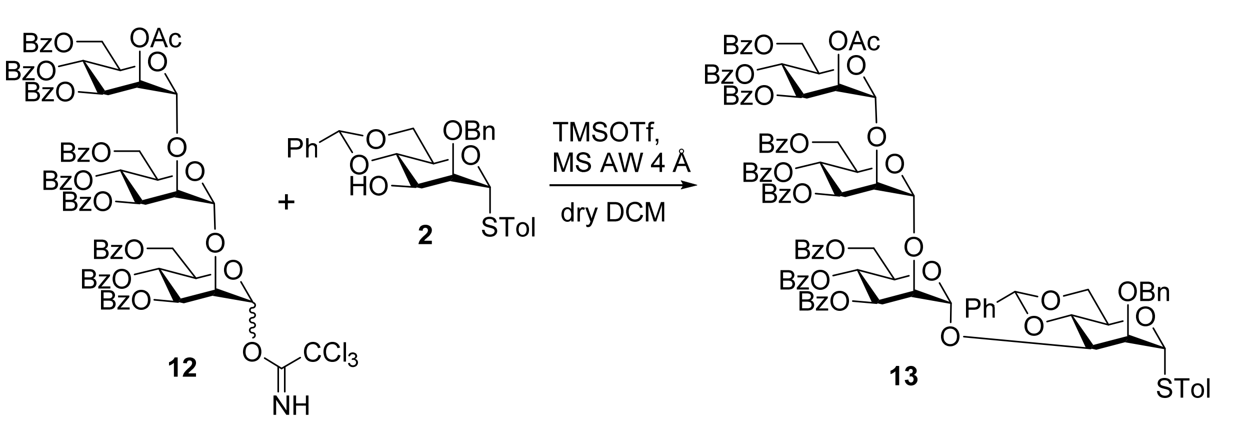

A suspension of **2** (0.38 g; 0.815 mmol), **12**[3] (1.46 g; 0.897 mmol; 1.1 eq.) and powdered acid-washed molecular sieves 4 Å (2 g) in dry DCM (20 ml) was stirred under Ar for 20 min at RT. Then TMSOTf (0.015 ml; 0.08 mmol; 0.1 eq.) was added dropwise and stirring was continued until complete consumption of the acceptor **8** (1 h). Work-up and chromatography as described above afforded **13** (1.45 g, 92%) as colorless syrup.

4-Methylphenyl 2-*O*-acetyl-3,4,6-tri-*O*-benzoyl-α-D-mannopyranosyl-(1→2)-3,4,6-tri-*O*-benzoyl-α-D-mannopyranosyl-(1→2)-3,4,6-tri-*O*-benzoyl-α-D-mannopyranosyl-(1→3)-2,4-di-*O*-benzyl-1-thio-α-D-mannopyranoside (14)

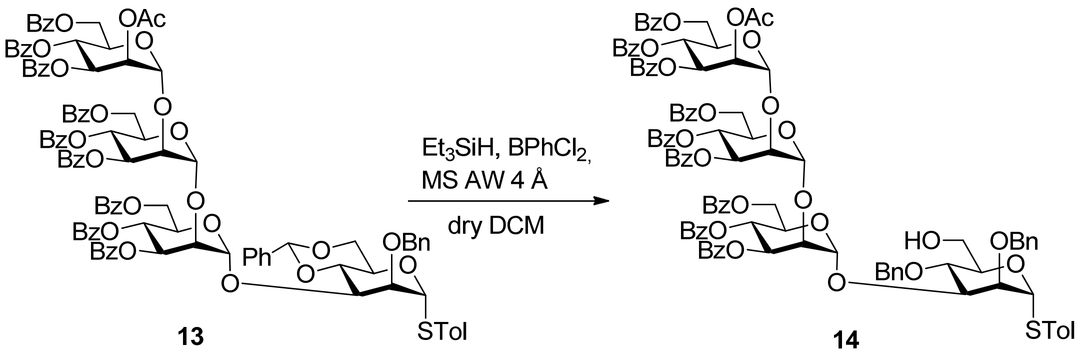

A suspension of **13** (107 mg; 0.055 mmol) and powdered acid-washed MS 4 Å in dry DCM (2 ml) was stirred under Ar for 30 min and then cooled down to -78 °C. Et_3SiH was added (0.027 ml; 0.166 mmol; 3 eq), followed by the dropwise addition of $BPhCl_2$ (0.025 ml; 0.188 mmol; 3.4 eq.). The mixture was stirred for 30 min at -78 °C and was then quenched by addition of NEt_3 followed by addition of MeOH. The solution was washed with aq satd $NaHCO_3$ and the aqueous phase was twice extracted with DCM. The combined organic phases were dried (Na_2SO_4), the solvent was removed *in vacuo* and the crude product was purified by silica flash chromatography (hexane/EtOAc = 2/1 → 1/1) to afford **14** (98 mg; 92%) as a colorless syrup; $[\alpha]_D^{21}$ +37.2 (*c* 1.1, $CHCl_3$); ^1H NMR (600 MHz, $CDCl_3$): δ = 8.10-8.03 (m, 6 H, Ar), 7.99-7.78 (m, 10 H, Ar), 7.54-7.21 (m, 40 H, Ar), 7.16-7.13 (m, 2 H, Ar), 7.08 (t, *J* = 7.5 Hz, 1 H, Ar), 6.06 (t, *J* = 10.0 Hz, 1 H, H-4''), 6.01 (t, *J* = 9.7 Hz, 1 H, H-4'), 5.95 (dd, *J* = 9.7, 2.8 Hz, 1 H , H-3'), 5.94 (dd, *J* = 10.0, 3.3 Hz, 1 H, H-3''), 5.87-5.82 (m, 2 H, H-4''', H-3'''), 5.62 (dd, *J* = 2.6, 2.3 Hz, 1 H, H-2'''), 5.60-5.57 (m, 2 H, H-1', H-1), 5.35 (d, *J* = 1.3 Hz, 1 H, H-1''), 4.91 (d, *J* = 1.3 Hz, 1 H, H-1'''), 4.90 (d, *J* = 11.4 Hz, 1 H, OC*H*$_2$Ar), 4.82 (d, *J* = 12.0 Hz, 1 H, OCH_2Ar), 4.68 (d, *J* = 11.4 Hz, 1 H, OC*H*$_2$Ar), 4.65 (d, *J* = 12.0 Hz, 1 H, OC*H*$_2$Ar), 4.59 (dd, *J* = 12.0, 2.7 Hz, 1 H, H-6a'), 4.53 (dd, *J* = 2.8, 2.3 Hz, 1 H, H-2'), 4.53-4.47 (m, 3 H, H-2'', H-5', H-5''), 4.46-4.38 (m, 3 H, H-6b', H-6a'', H-6b''), 4.33-4.24 (m, 3 H, H-4, H-5''', H-2), 4.19-4.13 (m, 2 H, H-3, H-5), 4.10 (d, *J* = 12.7 Hz, 1 H, H-

6a'''), 4.03 (d, J = 12.7 Hz, 1 H, H-6b'''), 3.80 (dd, J = 11.8, 5.1 Hz, 1 H, H-6a), 3.77-3.71 (m, 1 H, H-6b), 2.36 (s, 3 H, CH_3ArS), 2.04 (s, 3 H, CH_3C=O); ^{13}C NMR (150 MHz, $CDCl_3$): δ = 168.9 ($COCH_3$), 166.3, 166.2, 165.7, 165.6, 165.5, 165.2, 165.1, 164.9 (9 C, ArC=O), 138.2-127.5 (72 C, Ar), 100.6 (2 C, C-1', C-1''), 99.5 (C-1'''), 85.3 (C-1), 79.4 (C-2), 77.3 (C-2'), 76.6 (C-2''), 75.1 (OCH_2Ar), 74.6 (C-3), 73.1 (C-5), 71.6 (OCH_2Ar), 71.1 (C-3'), 70.6 (C-3''), 69.5 (3 C, C-5'', C-2''', C-4), 69.4 (2 C, C-5', C-3''), 69.3 (C-5'''), 67.4 (C-4'), 67.1 (2 C, C-4'', C-4'''), 63.8 (C-6'), 63.3 (C-6''), 62.8 (C-6'''), 61.9 (C-6), 21.1 (CH_3ArS), 20.5 (CH_3CO). ESI-TOF HRMS: *m/z* calcd for $C_{110}H_{98}O_{30}S$ $[M+NH_4^+]^+$: 1948.6202; found: 1948.6241.

4-Methylphenyl 2-*O*-acetyl-3,4,6-tri-*O*-benzoyl-α-D-mannopyranosyl-(1→2)-3,4,6-tri-*O*-benzoyl-α-D-mannopyranosyl-(1→2)-3,4,6-tri-*O*-benzoyl-α-D-mannopyranosyl-(1→3)-[2-*O*-benzoyl-3-*O*-benzyl-4,6-*O*-benzylidene-α-D-mannopyranosyl-(1→6)]-2,4-di-*O*-benzyl-1-thio-α-D-mannopyranoside (15)

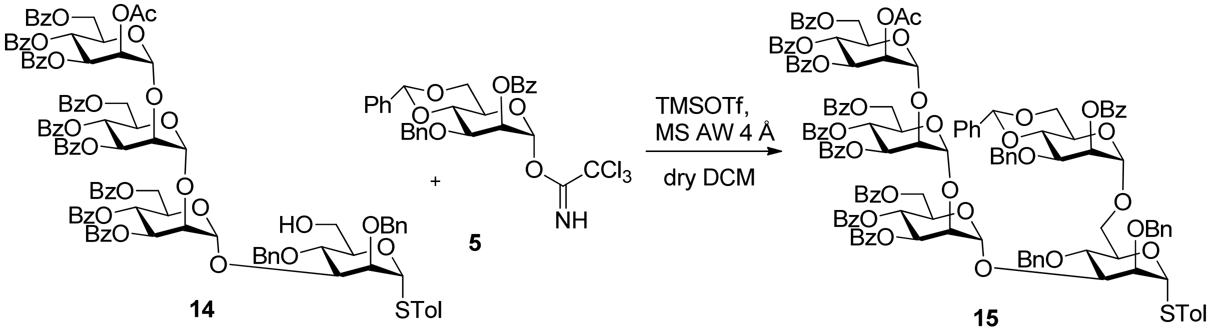

A mixture of **14** (0.317 g; 0.164 mmol), **5** (0.159 g; 0.263 mmol; 1.6 eq) and powdered acid washed molecular sieves 4 Å (0.4 g) in dry DCM (4 ml) was stirred for 20 min under Ar at RT. Then TMSOTf (3 μl; 0.016 mmol; 0.1 eq) was added and the suspension was stirred for 45 min at RT until complete consumption of the acceptor **14**. The reaction mixture was quenched by the addition of 3 drops of NEt_3, followed by filtration over a pad of Celite®. The filtrate was concentrated and the crude product was purified by silica flash chromatography (toluene/EtOAc = 20/1 → 10/1) to give **15** (0.355 g; 91 %) as colorless syrup; $[\alpha]_D^{21}$ +22.2 (*c* 1.0, in $CHCl_3$); ^{1}H NMR (600 MHz, $CDCl_3$): δ = 8.12-8.03 (m, 8 H, Ar), 7.96-7.78 (m, 12 H, Ar), 7.61-7.17 (m, 51 H, Ar), 7.12-7.04 (m, 3 H, Ar), 6.07 (t, J = 9.9 Hz, 1 H, H-4^C), 6.04 (t, J = 10.4 Hz, 1 H, H-4^B), 5.95 (dd, J = 9.4, 2.4 Hz, 1 H, H-3^B), 5.94 (dd, J = 10.0, 3.1 Hz, 1 H, H-3^C), 5.83 (t, J = 10.4 Hz, 1 H, H-4^D), 5.82 (dd, J = 10.4, 3.2 Hz, 1 H, H-3^D), 5.69-5.67 [m, 2 H, H-2^E, ArCH$(CHOR)_2$], 5.65 (s, 1 H, H-1^A), 5.61 (dd, J = 3.2, 2.5 Hz, 1 H, H-2^D), 5.57 (s, 1 H, H-1^B), 5.34 (s, 1 H, H-1^C), 4.97 (d, J = 1.7 Hz, 1 H, H-1^E), 4.95 (d, J = 11.5 Hz, 1 H, OCH_2Ar), 4.90 (d, J = 12.3 Hz, 1 H, OCH_2Ar),

4.88 (d, J = 1.6 Hz, 1 H, H-1^D), 4.66 (d, J = 12.2 Hz, 1 H, OCH_2Ar), 4.62-4.55 (m, 5 H, OCH_2Ar, OCH_2Ar, H-6a^C, H-2^B), 4.51-4.48 (m, 3 H, H-5^B, H-5^C, H-2^C), 4.48-4.41 (m, 3 H, H-6b^C, H-6a^B, H-6b^B), 4.32-4.23 (m, 5 H, H-6a^E, H-3^A, H-2^A, H-5^D, H-5^A), 4.18 (t, J = 9.6 Hz, 1 H, H-4^E), 4.12 (dd, J = 9.9, 3.3 Hz, 1 H, H-3^E), 4.06-4.00 (m, 3 H, H-4^A, H-6a^D, H-6b^D), 3.54 (ddd, J = 14.6 and 2 x 4.8 Hz, 1 H, H-5^E), 3.90-3.83 (m, 2 H, H-6b^E, H-6b^A), 3.65 (dd, J = 11.3, 1.8 Hz, 1 H, H-6a^A), 2.19 (s, 3 H, CH_3Ar), 2.03 (s, 3 H, CH_3C=O); ^{13}C NMR (150 MHz, $CDCl_3$): δ = 169.0 (CH_3*C*=O), 166.3, 166.2, 165.6, 165.5, 165.3, 165.3, 165.2, 165.0, 163.3 (10 C, Ar*C*=O), 138.2-125.2 (90 C, Ar), 101.6 [ArCH(CHOR)$_2$], 101.2 (C-1^B), 100.7 (C-1^C), 99.5 (C-1^D), 99.1 (C-1^E), 84.7 (C-1^A), 79.5 (C-2^A), 78.7 (C-4^E), 77.5 (C-2^B), 77.2 (C-2^C), 75.3 (O*C*H_2Ar), 74.7 (C-4^A), 73.8 (C-3^E), 72.1 (C-5^A), 71.9 (C-2^A), 71.3 and 71.2 (O*C*H_2Ar), 70.7 (C-3^B), 70.0 (C-2^E), 69.5 (C-3^D, C-2^D), 69.4 (C-5^B), 69.2 (C-5^C), 68.8 (C-6^E), 67.2 (C-5^D), 67.1 (C-4^D), 66.9 (2 C, C-4^C, C-6^A), 64.0 (C-5^E), 63.8 (C-6^C), 63.3 (C-6^B), 62.7 (C-6^D), 20.9 (*C*H_3ArS), 20.5 (*C*H_3C=O). ESI-TOF HRMS: *m/z* calcd for $C_{137}H_{122}O_{36}S$ [M+Na$^+$]$^+$: 2398.7362; found: 2398.7376.

3-Azido-1-propyl 2-*O*-acetyl-3,4,6-tri-*O*-benzoyl-α-D-mannopyranosyl-(1→2)-3,4,6-tri-*O*-benzoyl-α-D-mannopyranosyl-(1→2)-3,4,6-tri-*O*-benzoyl-α-D-mannopyranosyl-(1→3)-[2-*O*-benzoyl-3-*O*-benzyl-4,6-*O*-benzylidene-α-D-mannopyranosyl-(1→6)]-2,4-di-*O*-benzyl-D-mannopyranoside (16)

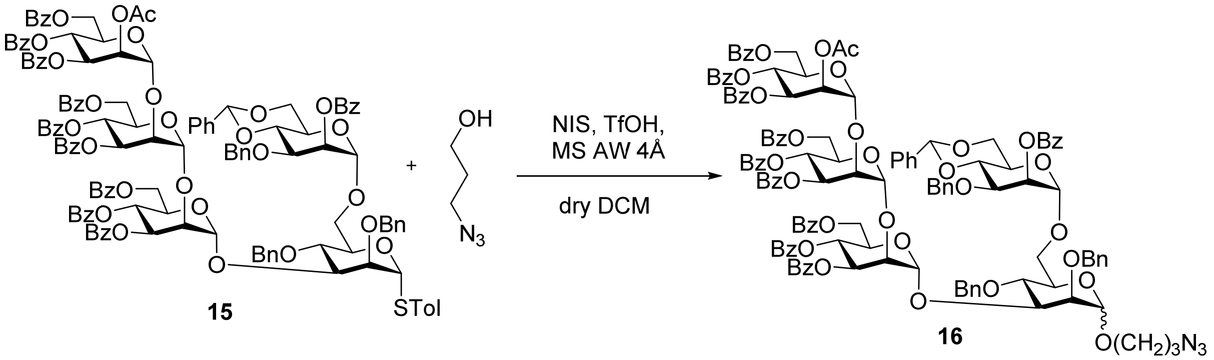

A mixture of **15** (0.200 g; 0.084 mmol), 3-azido-propan-1-ol (13 mg; 0.126 mmol; 1.5 eq) and powdered acid-washed molecular sieves 4 Å (0.4 g) in dry DCM (2 ml) was stirred for 30 min at room temperature under Ar. The suspension was cooled to 0 °C and NIS (25 mg; 0.109 mmol; 1.3 eq) was added followed by the addition of TfOH (1 μl diluted with 0.1 ml dry DCM; 0.08 mmol). The mixture was stirred for 2 h at 0 °C until complete consumption of the donor; then 3 drops of NEt_3 were added followed by filtration through a pad of Celite®. The filtrate was diluted with DCM and washed with aq $Na_2S_2O_3$. The organic phase was dried (Na_2SO_4) and the solvent was removed *in vacuo*. The crude product was purified by silica flash chromatography

(hexane/EtOAc = 2.5/1 → 3/2) to furnish an anomeric mixture (α/β=1/0.4) of **16** (0.165 g; 83 %) as colorless syrup. The mixture was used in the next step. ESI-TOF HRMS: *m/z* calcd for $C_{133}H_{121}N_3O_{37}$ [M+NH$_4^+$]$^+$: 2369.8017; found: 2369.8074.

3-Azido-1-propyl 2-*O*-acetyl-3,4,6-tri-*O*-benzoyl-α-D-mannopyranosyl-(1→2)-3,4,6-tri-*O*-benzoyl-α-D-mannopyranosyl-(1→2)-3,4,6-tri-*O*-benzoyl-α-D-mannopyranosyl-(1→3)-[2-*O*-benzoyl-3,6-di-*O*-benzyl-α-D-mannopyranosyl-(1→6)]-2,4-di-*O*-benzyl-β-D-mannopyranoside (17) and 3-azido-1-propyl 2-*O*-acetyl-3,4,6-tri-*O*-benzoyl-α-D-mannopyranosyl-(1→2)-3,4,6-tri-*O*-benzoyl-α-D-mannopyranosyl-(1→2)-3,4,6-tri-*O*-benzoyl-α-D-mannopyranosyl-(1→3)-[2-*O*-benzoyl-3,6-di-*O*-benzyl-α-D-mannopyranosyl-(1→6)]-2,4-di-*O*-benzyl-α-D-mannopyranoside (18)

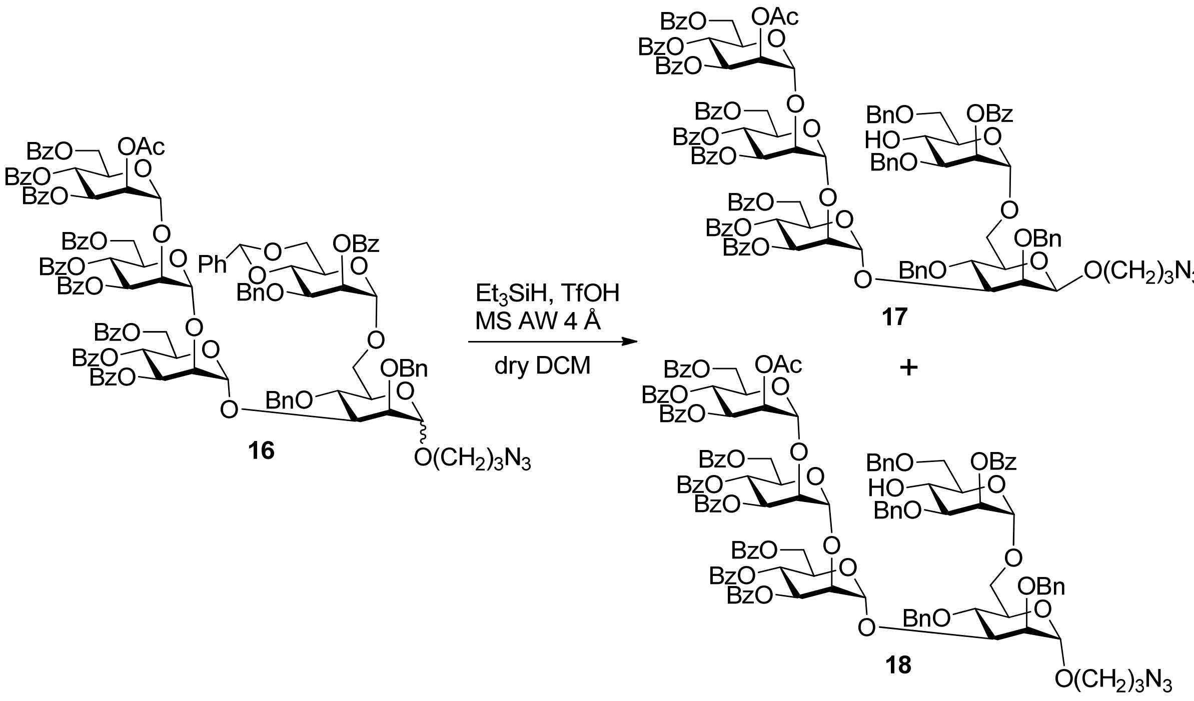

A suspension of **16** (0.164 g; 0.055 mmol; α/β = 1/0.5) and AW MS 4 Å in dry DCM (2 ml) under Ar was stirred for 1 h at RT and was then cooled to -78 °C. Subsequently Et_3SiH (0.033 ml; 0.209 mmol) was added followed by the dropwise addition of TfOH (21 µl; 0.237 mmol). The mixture was stirred for 2 h at -78 °C and after a second addition of TfOH (10 µl; 0.12 mmol) stirring was continued for 1 h. The reaction was quenched by the addition of NEt_3 followed by addition of MeOH. Satd aqu $NaHCO_3$ was added, the phases were separated and the aqueous phase was twice extracted with DCM. The combined organic phases were dried (Na_2SO_4) the solvent was removed *in vacuo* and the crude product was purified by HPLC (column: YMC-pack-

sil-06, toluene/EtOAc = 15/1 → 8/1) to give **17** (32 mg; 20 %) followed by **18** (85 mg; 52 %) as colourless syrups. Data for **17**: R_f = 0.21 (toluene/EtOAc = 10/1); $[\alpha]_D^{21}$ +1.6 (*c* 0.7, $CHCl_3$); δ = 8.05-7.76 (m, 17 H, Ar), 7.58-7.03 (m, 53 H, Ar), 6.02 (t, *J* = 10.1 Hz, 1 H, H-4^C), 5.99 (t, *J* = 10.0 Hz, 1 H, H-4^B), 5.93 (dd, *J* = 10.1, 3.1 Hz, 1 H, H-3^B), 5.90 (dd, *J* = 9.9, 3.2 Hz, 1 H, H-3^C), 5.82 (t, *J* = 9.4 Hz, 1 H, H-4^D), 5.79 (dd, *J* = 9.4, 3.8 Hz, 1 H, H-3^D), 5.64 (dd, *J* = 3.1, 2.1 Hz, 1 H, H-2^E), 5.60 (dd, *J* =2.6, 2.2 Hz, 1 H, H-2^D), 5.51 (d, *J* = 2.1 Hz, 1 H, H-1^B), 5.22 (d, *J* = 1.7 Hz, 1 H, H-1^C), 5.20 (d, *J* = 12.9 Hz, 1 H, OCH_2Ar), 5.01 (d, *J* = 2.0 Hz, 1 H, H-1^E), 4.87 (s, 1 H, H-1^D), 4.86 (d, *J* = 10.7 Hz, 1 H, OCH_2Ar), 4.78 (d, *J* = 12.9 Hz, 1 H, OCH_2Ar), 4.71 (d, *J* = 11.7 Hz, 1 H, OCH_2Ar), 4.66 (d, *J* = 11.8 Hz, 1 H, OCH_2Ar), 4.57-4.37 (m, 10 H, OCH_2Ar, H-6a^B, H-2^B, H-2^C, H-6a^C, H-6b^C, H-5^B, H-5^C, H-6b^B), 4.30 (s, 1 H, H-1^A), 4.23-4.18 (m, 1 H, H-5^D), 4.14 (ddd, *J* = 2 x 9.5, 2.0 Hz, 1 H, H-4^E), 4.03-3.94 (m, 3 H, H-2^A, H-6a^D, H-6b^D), 3.90 (t, *J* = 9.5 Hz, 1 H, H-4^A), 3.88-3.78 (m, 5 H, $OCH_2CH_2CH_2N_3$, H-3^E, H-5^E, H-6a^A, H-6a^E), 3.76 (dd, *J* = 2.9, 9.5 Hz, 1 H, H-3^A), 3.73 (dd, *J* = 2.3, 10.0 Hz, 1-H, H-6b^E), 3.66 (dd, *J* = 10.9, 2.0 Hz, 1 H, H-6b^A), 3.48 (ddd, *J* = 9.7, 6.9, 5.3 Hz, 1 H, $OCH_2CH_2CH_2N_3$), 3.36-3.27 (m, 2 H $OCH_2CH_2CH_2N_3$), 3.26 (ddd, *J* = 9.6, 5.9, 2.2 Hz, 1 H, H-5^A), 2.41 (d, *J* = 2.27, 1 H, OH), 2.01 (s, 3 H, CH_3C=O); ^{13}C NMR (150 MHz, $CDCl_3$): δ = 169.0 (CH_3C=O), 166.2, 165.7, 165.6, 165.5, 165.3, 165.2, 165.0 (10 C, ArC=O), 139.1-127.2 (84 C, Ar), 101.6 (C-1^A), 101.3 (C-1^B), 101.0 (C-1^C), 99.4 (C-1^D), 98.2 (C-1^E), 83.1 (C-3^A), 78.4 (C-2^A), 78.1 (C-2^B), 77.2 (C-3^E), 76.3 (C-2^C), 75.2 (OCH_2Ar), 74.8 (OCH_2Ar), 74.7 (C-5^A), 74.4 (C-4^A), 71.4 (C-5^E), 71.1 (C-3^B), 71.0 (OCH_2Ar), 70.8 (C-3^C), 69.8 (C-6^E), 69.75 (C-3^D), 69.5 (C-2^D, C-5^B or C-5^C), 69.2 (C-5^D), 68.1 (C-2^E), 67.3 and 67.2 (3 C, C-4^B, C-4^C, C-4^E), 66.7 (2 C, $OCH_2CH_2CH_2N_3$, C-6^A), 63.8 and 63.6 (C-6^B, C-6^C), 62.8 (C-6^D), 48.5 ($OCH_2CH_2CH_2N_3$), 29.4 ($OCH_2CH_2CH_2N_3$), 20.5 (CH_3C=O). ESI-TOF HRMS: *m/z* calcd for $C_{133}H_{123}N_3O_{37}$ $[M+NH_4^+]^+$: 2372.8207; found: 2372.8288. Data for **18**: R_f = 0.18 (toluene/EtOAc = 10/1); $[\alpha]_D^{21}$ +23.6 (*c* 1.0, $CHCl_3$); ^1H NMR (600 MHz, $CDCl_3$): δ = 8.07-7.75 (m, 20 H, Ar), 7.57-6.98 (m, 50 H, Ar), 6.09 (t, *J* = 10.0 Hz, 1 H, H-4^C), 6.06 (t, *J* = 10.7 Hz, 1 H, H-4^B), 5.96 (dd, *J* = 10.7, 3.0 Hz, 1 H, H-3^B), 5.92 (dd, *J* = 10.0, 3.2 Hz, 1 H, H-3^C), 5.84 (t, *J* = 9.7 Hz, 1 H, H-4^D), 5.80 (dd, *J* = 10.7, 3.2 Hz, 1 H, H-3^D), 5.68 (dd, *J* = 3.1, 1.9 Hz, 1 H, H-2^E), 5.59 (t, *J* = 2.6 Hz, 1 H, H-2^D), 5.51 (d, *J* = 1.6 Hz, H-1^B), 5.32 (d, *J* = 1.9 Hz, 1 H, H-1^C), 5.09 (d, *J* = 1.9 Hz, 1 H, H-1^E), 4.94 (d, *J* = 1.4 Hz, 1 H, H-1^A), 4.92 (d, *J* = 11.2 Hz, 1 H, OCH_2Ar), 4.89 (d, *J* = 12.1 Hz, 1 H, OCH_2Ar), 4.85 (d, *J* = 1.3 Hz, 1 H, H-1^D), 4.72-4.68 (m, 2 H, OCH_2Ar), 4.66 (d, *J* = 11.8 Hz, 1 H, H-6a^B), 4.60-4.52 (m, 5 H, H-2^B, H-2^C, H-5^B, OCH_2Ar), 4.49 (dd, *J* = 2.6, 1.9 Hz, 1 H, H-2^C), 4.48-4.44 (m, 2 H, H-5^C, H-6b^B), 4.41 (d, *J* = 11.4 Hz, 1 H, OCH_2Ar), 4.39-4.36 (m, 2 H, H-6a^C, H-6b^C), 4.28-4.21 (m, 2 H, H-5^D, H-3^A), 4.16 (ddd, *J* = 2 x 9.5, 2.0 Hz, 1 H, H-4^E), 4.06-3.97 (m, 4 H, H-4^A, H-2^A, H-6a^D, H-6b^D), 3.88 (dd, *J* = 3.1, 9.5 Hz, 1 H, H-3^E), 3.87 (dd, *J* = 4.9, 10.3 Hz, H-

6a^A), 3.84-3.74 (m, 2 H, H-5^E, H-6a^E), 3.76-3.70 (m, 4 H, H-6b^A, H-5^A, H-6b^E, OCH_2CH$_2$CH$_2$N$_3$), 3.43 (ddd, J = 10.1, 2 x 6.2 Hz, 1 H, OCH_2CH$_2$CH$_2$N$_3$), 3.33-3.25 (m, 2 H, OCH$_2$CH$_2$CH_2N$_3$), 2.48 (d, J = 2.0 Hz, 1 H, OH), 2.01 (s, 3 H, CH$_3$C=O); ^{13}C NMR (150 MHz, CDCl$_3$): δ = 169.0 (CH$_3$*C*=O), 166.3, 166.2, 165.7, 165.6, 165.5, 165.3, 165.1, 165.0 (10 C, Ar*C*=O), 138.5-127.4 (84 C, Ar), 101.3 (C-1^B), 100.8 (C-1^C), 99.5 (C-1^D), 98.3 (C-1^E), 96.9 (C-1^A), 81.4 (C-3^A), 78.3 (C-2^A), 77.6 (C-2^B), 77.1 (C-3^E), 76.7 (C-2^C), 75.3 (OCH$_2$Ar), 74.5 (C-4^A), 73.6 (OCH$_2$Ar), 72.1 (OCH$_2$Ar), 71.6 and 71.5 C-5^A, C-5^E), 71.4 (C-3^B), 71.0 (OCH$_2$Ar), 70.7 (C-3^C), 69.7 (C-6^E), 69.5 and 69.4 (C-2^D, C-3^D, C-5^B, C-5^C), 69.3 (C-5^D), 68.1 (C-2^E), 67.4 (C-4^B), 67.3 (C-4^E), 67.2 (2 C, C-4^B, C-4^C), 66.9 (C-4^D), 66.5 (C-6^A), 64.3 (O*C*H$_2$CH$_2$CH$_2$N$_3$), 63.8 (C-6^B), 63.1 (C-6^C), 62.6 (C-6^D), 48.4 (OCH$_2$CH$_2$*C*H$_2$N$_3$), 28.8 (OCH$_2$*C*H$_2$CH$_2$N$_3$), 20.6 (*C*H$_3$C=O). ESI-TOF HRMS: *m/z* $C_{133}H_{123}N_3O_{37}$ calcd for [M+NH$_4^+$]$^+$: 2372.8207; found: 2372.8181.

3-Azido-1-propyl 2-*O*-acetyl-3,4,6-tri-*O*-benzoyl-α-D-mannopyranosyl-(1→2)-3,4,6-tri-*O*-benzoyl-α-D-mannopyranosyl-(1→2)-3,4,6-tri-*O*-benzoyl-α-D-mannopyranosyl-(1→3)-[2-*O*-benzoyl-3,4-di-*O*-benzyl-α-D-mannopyranosyl-(1→6)]-2,4-di-*O*-benzyl-α-D-mannopyranoside (19) and 3-azido-1-propyl 2-*O*-acetyl-3,4,6-tri-*O*-benzoyl-α-D-mannopyranosyl-(1→2)-3,4,6-tri-*O*-benzoyl-α-D-mannopyranosyl-(1→2)-3,4,6-tri-*O*-benzoyl-α-D-mannopyranosyl-(1→3)-[2-*O*-benzoyl-3,4-di-*O*-benzyl-α-D-mannopyranosyl-(1→6)]-2,4-di-*O*-benzyl-β-D-mannopyranoside (20)

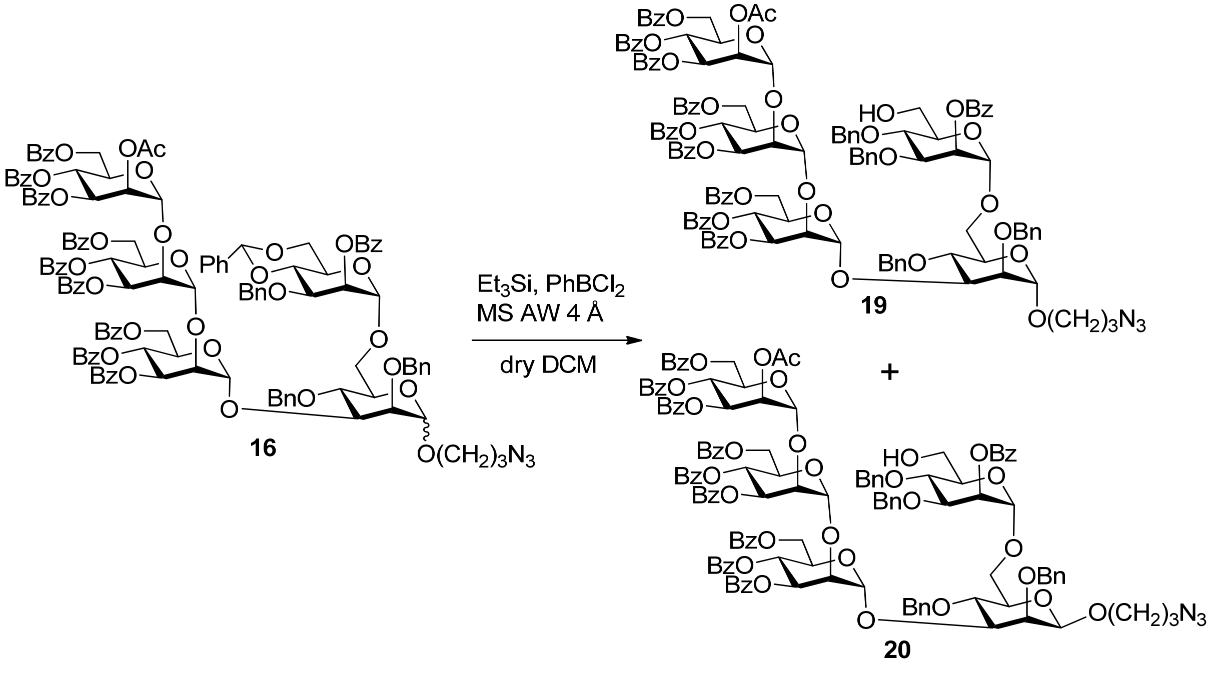

A suspension of **16** (0.30 g; 0.127 mmol; α/β = 1/0.5) and AW MS 4 Å in dry DCM (4 ml) under Ar was stirred for 1 h at RT and was then cooled down to -78 °C. Subsequently Et_3SiH (61 μl; 0.382 mmol; 3 eq.) was added followed by the dropwise addition of $BPhCl_2$ (56 μl; 0.433 mmol; 3.4 eq.). The mixture was stirred for 1 h at -78 °C and was then quenched by addition of NEt_3 followed by addition of MeOH. Satd aqu $NaHCO_3$ was added to the mixture, phases were separated and the aqueous phase was twice extracted with DCM. The combined organic phases were dried (Na_2SO_4), the solvent was removed *in vacuo*. The residue was purified by HPLC (column: YMC-pack-sil-06, toluene/EtOAc = 15/1 → 8/1) to give **19** (155 mg; 52 %) followed by **20** (62 mg; 21 %) as colourless syrups. Data for **19**: R_f = 0.13 (toluene/EtOAc = 10/1); $[\alpha]_D^{21}$ +30 (*c* 1.2, $CHCl_3$); ^1H NMR (600 MHz, $CDCl_3$): δ = 8.11-8.01 (m, 8 H, Ar), 7.97-7.77 (m, 12 H, Ar), 7.62-7.17 (m, 49 H, Ar), 7.02 (t, *J* = 8.3 Hz, 1 H, Ph), 6.09 (dd, *J* = 10.0 Hz, 1 H, H-4^C), 6.07 (t, *J* = 10.1 Hz, 1 H, H-4^B), 5.97 (dd, *J* = 10.1, 3.0 Hz, 1 H, H-3^B), 5.93 (dd, *J* = 9.9, 3.2 Hz, 1 H, H-3^C), 5.84 (t, *J* = 9.7 Hz, 1 H, H-4^D), 5.81 (dd, *J* = 9.7, 3.2 Hz, 1 H, H-3^D), 5.69 (t, *J* = 2.5 Hz, 1 H, H-2^E), 5.61 (dd, *J* = 3.2, 1.3 Hz, 1 H, H-2^D), 5.51 (d, *J* = 1.1 Hz, 1 H, H-1^B), 5.35 (d, *J* = 1.3 Hz, 1 H, H-1^C), 5.05 (d, *J* = 1.8 Hz, 1 H, H-1^E), 4.94 (br s, H-1^A), 4.94-4.89 (m, 3 H, OCH_2Ar, OCH_2Ar), 4.88 (d, *J* = 1.3 Hz, 1 H, H-1^D), 4.72 (d, J = 11.5 Hz, 1 H, OCH_2Ar), 4.71 (d, *J* = 12.0 Hz, 1 H, H-6_b), 4.66 (d, *J* = 11.1 Hz, 1 H, OCH_2Ar), 4.59-4.49 (m, 6 H, H-2^B, H-2^C, H-5^B, H-$6a^B$, H-$6a^C$, OCH_2Ar), 4.49-4.44 (m, 2 H, H-5^C, H-$6b^B$), 4.39-4.37 (m, 2 H, H-$6a^C$, H-$6b^C$), 4.28-4.24 (m, 2 H, H-3^A, H-5^D), 4.10 (dd, *J* = 9.3, 2.5 Hz, 1 H, H-3^E), 4.08-3.95 (m, 5 H, H-4^A, H-2^A, H-$6a^D$, H-$6b^D$, H-4^E), 3.82-3.72 (m, 6 H, H-5^E, H-5^A, H-$6a^E$, H-$6b^E$, H-$6a^A$, $OCH_2CH_2CH_2N_3$), 3.70 (dd, *J* = 11.7, 1.7 Hz, 1 H, H-$6b^A$), 3.43 (ddd, *J* = 11.7, 2 x 6.1 Hz, 1 H, $OCH_2CH_2CH_2N_3$), 3.34-3.24 (m, 2 H, $OCH_2CH_2CH_2N_3$), 2.03 (s, 3 H, CH_3C=O), 1.77 (ddd, *J* = 12.9, 6.5 Hz, 6.5 Hz, 2 H, $OCH_2CH_2CH_2N_3$); ^{13}C NMR (150 MHz, $CDCl_3$): δ = 169.0 (CH_3C=O), 166.2, 165.6, 165.5, 165.4, 165.3, 165.2, 165.1, 164.9 (10 C, ArC=O), 138.9-127.5 (84 C, Ar), 100.7 (C-1^B), 100.6 (C-1^C), 99.5 (C-1^D), 97.9 (C-1^E), 96.8 (C-1^A), 80.7 (C-3^A), 78.1 (C-2^A), 77.7 (C-3^E), 77.5 (C-2^B), 76.7 (C-2^C), 75.1 (2 C, 2 x OCH_2Ar), 74.6 and 74.0 (C-4^A, C-4^E), 72.0 (C-6^A), 71.9 (2 C, OCH_2Ar, C-5^E), 71.4 (C-5^A), 71.3 (C-5^E), 71.1 (C-3^B), 70.7 (C-3^C), 69.5 and 69.4 (4 C, C-3^D, C-5^B, C-5^C, C-5^D), 69.3 (C-2^D), 68.8 (C-2^E), 67.3 and 66.9 (C-4^B, C-4^C, C-4^D), 66.4 (C-6^A), 64.8 ($OCH_2CH_2CH_2N_3$), 63.7 (C-6^B), 63.1 (C-6^C), 62.6 (C-6^D), 62.0 (C-6^E), 48.3 $OCH_2CH_2CH_2N_3$), 28.7 ($OCH_2CH_2CH_2N_3$), 20.5 (CH_3C=O), ESI-TOF HRMS: *m/z* calcd for $C_{133}H_{123}N_3O_{37}$ $[M+Na^+]^+$: 2377.7761; found: 2377.7800. Data for **20**: R_f = 0.09 (toluene/EtOAc = 10/1); $[\alpha]_D^{21}$ +10.3 (*c* 1.0, $CHCl_3$); ^1H NMR (600 MHz, $CDCl_3$): δ = 8.10-7.78 (m, 20 H, Ar), 7.61-7.17 (m, 49 H, Ar), 7.08-7.05 (m, 1 H, Ar), 6.03 (t, *J* = 9.8 Hz, 1 H, H-4^B), 6.00 (t, *J* = 9.5 Hz, 1 H, H-4^C), 5.93 (dd, *J* = 9.8, 3.1 Hz, 1 H, H-3^B), 5.91 (dd, *J* = 9.8, 3.2 Hz, 1 H, H-3^C), 5.83 (t, *J* = 9.5 Hz, 1 H, H-4^D), 5.79 (dd, *J* = 9.5, 3.7 Hz,

1 H, H-3^D), 5.67 (dd, J = 3.1, 2.1 Hz, 1 H, H-2^E), 5.61 (dd, J = 2.7, 2.3 Hz, 1 H, H-2^D), 5.52 (d, J = 1.6 Hz, 1 H, H-1^B), 5.24 (d, J = 1.8 Hz, 1 H, H-1^C), 5.19 (d, J = 12.9 Hz, 1 H, OCH_2Ar), 4.97 (d, J = 2.2 Hz, 1 H, H-1^E), 4.92 (d, J = 10.9 Hz, 1 H, OCH_2Ar), 4.88 (s, 1 H, H-1^D), 4.87 (d, J = 11.7 Hz, 1 H, OCH_2Ar), 4.79 (d, J = 12.9 Hz, 1 H, OCH_2Ar), 4.72 (d, J = 11.6 Hz, 1 H, OCH_2Ar), 4.64 (d, J = 10.9 Hz, 1 H, OCH_2Ar), 4.57-4.36 (m, 10 H, H-5^B, H-5^C, H-2^B, H-2^C, H-6a^B, H-6b^B, H-6a^C, H-6b^C, OCH_2Ar), 4.29 (s, 1 H, H-1^A), 4.24-4.20 (m, 1 H, H-5^D), 4.06 (dd, J = 9.3, 3.1 Hz, 1 H, H-3^E), 4.04-3.97 (m, 3 H, H-2^A, H-6a^D, H-6b^D), 3.97 (t, J = 9.3 Hz, 1 H, H-4^E), 3.88 (t, J = 9.4 Hz, 1 H, H-4^A), 3.87-3.70 (m, 6 H, H-6a^A, H-5^E, $OCH_2CH_2CH_2N_3$, H-3^A, H-6a^E, H-6b^E), 3.64 (dd, J = 11.3, 2.0 Hz, 1 H, H-6b^A), 3.47 (ddd, J = 9.8, 7.1, 5.2 Hz, 1 H, $OCH_2CH_2CH_2N_3$), 3.32-3.22 (m, 3 H, H-5^A, $OCH_2CH_2CH_2N_3$), 2.01 (s, 3 H, CH_3C=O), 1.85-1.71 (m, 2 H, $OCH_2CH_2CH_2N_3$); ^{13}C NMR (150 MHz, $CDCl_3$): δ = 169.0 (CH_3C=O), 166.2, 165.7, 165.3, 165.2, 164.9 (10 C, ArC=O), 138.9-127.2 (84 C, Ar), 101.6 (C-1^A), 101.2 (C-1^B), 101.0 (C-1^C), 99.4 (C-1^D), 97.8 (C-1^E), 82.9 (C-3^A), 78.2 (C-2^A), 78.1 (C-2^B), 77.6 (C-3^E), 76.3 (C-2^C), 75.2 (OCH_2Ar), 75.1 (OCH_2Ar), 74.8 (OCH_2Ar), 74.6 (C-4^A), 74.2 (C-5^A), 73.9 (C-4^E), 71.9 (C-5^E), 71.1 (OCH_2Ar), 71.0 (C-3^C), 70.7 (C-3^B), 69.7 (C-5^C), 69.5 (3 C, C-5^B, C-3^D, C-2^D), 69.2 (C-5^D), 68.8 (C-2^E), 67.3 (2 C, C-4^C, C-4^B), 67.2 (C-4^D), 66.7 ($OCH_2CH_2CH_2N_3$), 66.5 (C-6^A), 63.7 and 63.6 (C-6^B, C-6^C), 62.8 (C-6^D), 62.0 (C-6^E), 48.4 ($OCH_2CH_2CH_2N_3$), 29.3 ($OCH_2CH_2CH_2N_3$), 20.5 (CH_3C=O). ESI-TOF HRMS: *m/z* calcd for $C_{133}H_{123}N_3O_{37}$ [M+NH_4^+]$^+$: 2372.8207; found: 2372.8145.

3-Azido-1-propyl α-D-mannopyranosyl-(1→2)-α-D-mannopyranosyl-(1→2)-α-D-mannopyranosyl-(1→3)-[α-D-mannopyranosyl-(1→6)]-α-D-mannopyranoside (NIT59A)

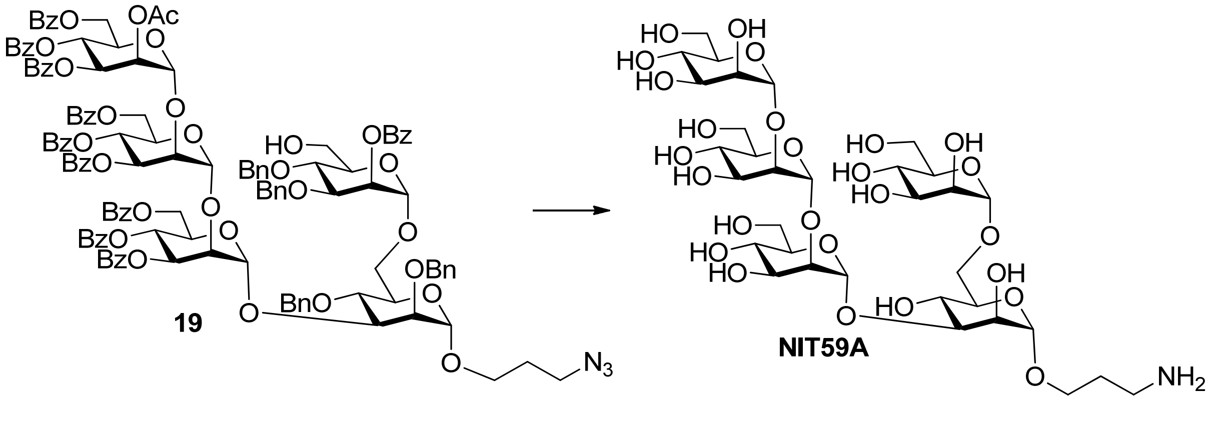

Deprotection was performed according to general method A using **19** (0.100 g; 0.042 mmol) in dry MeOH (2 ml) and NaOMe (1.7 ml; 0.17 mmol) for the deacylation and 10% Pd/C (46 mg) for the hydrogenation. Workup and purification gave **NIT59A** as colourless amorphous solid (37 mg;

99 %); $[\alpha]_D^{21}$ +80.6 (*c* 1.1, H_2O); ^1H NMR (600 MHz, D_2O): δ = 5.31 (d, *J* = 1.4 Hz, 1 H, H-1^B), 5.27 (d, *J* = 1.7 Hz, 1 H, H-1^C), 5.01 (d, *J* = 1.7 Hz, 1 H, H-1^D), 4.88 (d, *J* = 1.7 Hz, 1 H, H-1^E), 4.65 (s, 1 H, H-1^A); 4.11 (d, *J* = 2.7 Hz, 1 H, H-2^A), 4.08 (dd, *J* = 1.8, 3.2 Hz, 1 H, H-2^C), 4.07 (dd, *J* = 1.7, 3.2 Hz, 1 H, H-2^B), 4.05 (dd, *J* = 1.8, 3.4 Hz, 1 H, H-2^D), 3.97-3.92 (m, 4 H, H-3^B, OCH_2CH_2, H-2^E, H-$6a^A$), 3.92 (dd, *J* = 3.2, 9.6 Hz, 1 H, H-3^C), 3.88 -3.59 (m, 21 H), 3.52 (ddd, *J* = 1.9, 4.9, 9.9 Hz, 1 H, H-5^A), 3.11-3.06 (m, 2 H, $CH_2CH_2NH_2$), 1.99-1.93 (m, 2 H, $CH_2CH_2NH_2$). ^{13}C NMR (125 MHz, D_2O): δ = 103.0 (C-1^D), 101.6 and 101.5 (C-1^B, C-1^C), 100.7 (C-1^A), 100.3 (C-1^E), 81.7 (C-3^A), 79.5 and 79.3 (C-2^B, C-2^C), 75.0 (C-5^A), 74.2, 74.1, 74.0, 73.5 (C-5^B, C-5^C, C-5^D, C-5^E), 71.5, 71.2, 70.9 (d.i.), 70.8 (d.i.) and 70.7 (7 C, C-2^A, C-2^E, C-2^D, C-3^E, C-3^B, C-3^C, C-3^D), 68.2 (OCH_2CH_2), 67.8 (d.i.) and 67.6. (d.i., 4 C, C-4^B, C-4^C, C-4^D, C-4^E), 66.6 (C-4^A), 66.3 (C-6^A), 61.9 and 61.8 (4 C, C-6^B, C-6^C, C-6^D, C-6^E), 38.6 ($CH_2CH_2NH_2$), 27.9 ($CH_2CH_2NH_2$). ESI-TOF HRMS: *m/z* calcd for $C_{33}H_{59}NO_{26}$ $[M+H^+]^+$: 886.3398; found: 886.3391.

3-Azido-1-propyl α-D-mannopyranosyl-(1→2)-α-D-mannopyranosyl-(1→2)-α-D-mannopyranosyl-(1→3)-[α-D-mannopyranosyl-(1→6)]-β-D-mannopyranoside (NIT59B)

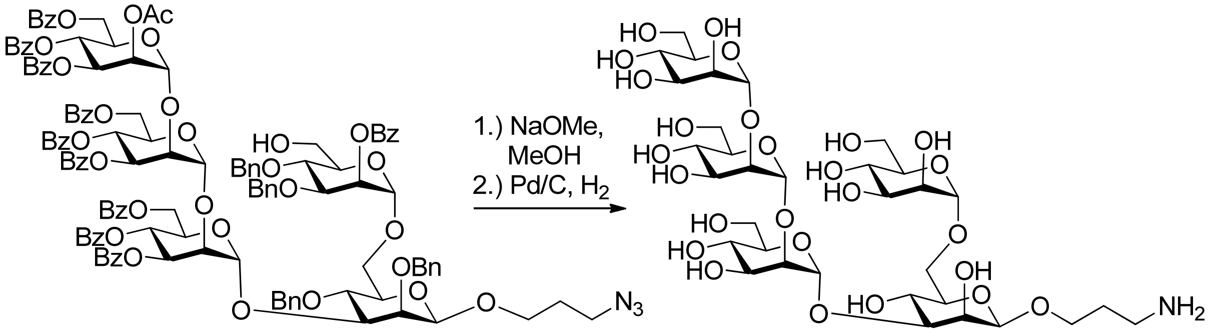

Deprotection was performed according to general method A using **20** (0.052 g; 0.022 mmol) in dry MeOH (2 ml) and NaOMe (0.9 ml; 0.088 mmol) for the deacylation and 10 % Pd/C (23 mg) for the hydrogenation. Workup and purification as described gave **NIT59B** as colourless amorphous solid (17 mg; 87 %); $[\alpha]_D^{21}$ +30.1 (*c* 1.0, H_2O); ^1H NMR (600 MHz, D_2O): δ = 5.31 (d, *J* = 1.4 Hz, 1 H, H-1^B), 5.27 (d, *J* = 1.7 Hz, 1 H, H-1^C), 5.01 (d, *J* = 1.7 Hz, 1 H, H-1^D), 4.88 (d, *J* = 1.7 Hz, 1 H, H-1^E), 4.65 (s, 1 H, H-1^A); 4.11 (d, *J* = 2.7 Hz, 1 H, H-2^A), 4.08 (dd, *J* = 1.8, 3.2 Hz, 1 H, H-2^C), 4.07 (dd, *J* = 1.7, 3.2 Hz, 1 H, H-2^B), 4.05 (dd, *J* = 1.8, 3.4 Hz, 1 H, H-2^D), 3.97-3.92 (m, 4 H, H-3^B, OCH_2CH_2, H-2^E, H-$6a^A$), 3.92 (dd, *J* = 3.2, 9.6 Hz, 1 H, H-3^C), 3.88 -3.59 (m, 21 H), 3.52 (ddd, *J* = 1.9, 4.9, 9.9 Hz, 1 H, H-5^A), 3.11-3.06 (m, 2 H, $CH_2CH_2NH_2$), 1.99-1.93 (m, 2 H, $CH_2CH_2NH_2$). ^{13}C NMR (125 MHz, D_2O): δ = 103.1 (C-1^D), 101.6 and 101.5 (C-1^B, C-1^C), 100.7

(C-1^A), 100.2 (C-1^E), 81.7 (C-3^A), 79.5 and 79.3 (C-2^B, C-2^C), 75.0 (C-5^A), 74.2, 74.1, 74.0, 73.5 (C-5^B, C-5^C, C-5^D, C-5^E), 71.5, 71.2, 70.9 (d.i.), 70.8 (d.i.) and 70.7 (7 C, C-2^A, C-2^D, C-2^E, C-3^B, C-3^C, C-3^D, C-3^E), 68.2 (O*C*$H_2$$CH_2$), 67.8 (d.i.) and 67.6. (d.i., 4 C, C-4^B, C-4^C, C-4^D, C-4^E), 66.6 (C-4^A), 66.3 (C-6^A), 61.9 and 61.8 (4 C, C-6^B, C-6^C, C-6^D, C-6^E), 38.6 (CH_2*C*H_2NH_2), 27.9 (*C*$H_2CH_2NH_2$). ESI-TOF HRMS: *m/z* calcd for $C_{33}H_{59}NO_{26}$ [M+H$^+$]$^+$: 886.3398; found: 886.3408.

3-Azido-1-propyl 2-*O*-acetyl-3,4,6-tri-*O*-benzoyl-α-D-mannopyranosyl-(1→2)-3,4,6-tri-*O*-benzoyl-α-D-mannopyranosyl-(1→2)-3,4,6-tri-*O*-benzoyl-α-D-mannopyranosyl-(1→3)-[2-*O*-acetyl-3,4,6-tri-*O*-benzoyl-α-D-mannopyranosyl-(1→2)-3,4,6-tri-*O*-benzoyl-α-D-mannopyranosyl-(1→4)-2-*O*-benzoyl-3,6-di-*O*-benzyl-α-D-mannopyranosyl-(1→6)]-2,4-di-*O*-benzyl-α-D-mannopyranoside (21)

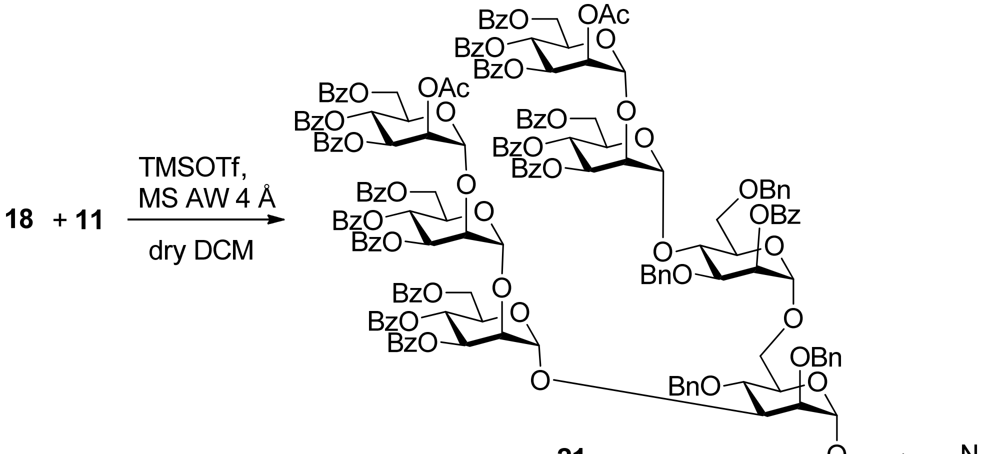

21

A suspension of **18** (50 mg; 0.021 mmol), **11** (37 mg; 0.032 mmol; 1.5 eq) and AW MS 4 Å in dry DCM (1 ml) under Ar was stirred for 20 min at RT. Then a solution of 0.2 M TMSOTf in dry DCM (10 µl; 0.1 eq) was added and the reaction mixture was stirred at RT for 3 h. The reaction was quenched by the addition of 3 drops of NEt_3 and the suspension was filtered over Celite®. The filtrate was concentrated and the residue was purified by flash chromatography (hexane/EtOAc = 3/1→1/1) to give **21** (48 mg; 75 %) as colorless syrup; $[\alpha]_D^{21}$ +24.2 (*c* 1.1, $CHCl_3$); ^{1}H NMR (600 MHz, $CDCl_3$): δ = 8.10-7.73 (m, 30 H, Ar), 7.60-7.00 (m, 70 H, Ar), 6.08 (t, *J* = 9.8 Hz, 1 H, H-4^C), 6.07 (t, *J* = 9.9 Hz, 1 H, H-4^B), 5.99 (dd, *J* = 9.9, 3.1 Hz, 1 H, H-3^B), 5.96 (t, *J* = 10.1 Hz, 1 H, H-4^F), 5.93 (dd, *J* = 9.9, 3.2 Hz, 1 H, H-3^C), 5.86-5.82 (m, 3 H, H-4^D, H-4^G, H-3^D), 5.81 (dd, *J* = 9.9, 3.1 Hz, 1 H, H-3^F), 5.77 (dd, *J* = 9.9, 3.2 Hz, 1 H, H-3^G), 5.67 (dd, *J* = 2.9, 2.0 Hz, 1 H, H-2^E), 5.61 (app t, *J* = 2.6 Hz, 1 H, H-2^D), 5.51 (d, *J* = 1.4 Hz, 1 H, H-1^B), 5.50 (dd, *J* = 3.1, 2.0 Hz, 1 H, H-2^G), 5.42 (d, *J* = 1.7 Hz, 1 H, H-1^F), 5.35 (d, *J* = 1.8 Hz, 1 H, H-1^C), 5.13 (d, *J* = 1.5 Hz, 1 H, H-

1^E), 5.01 (d, *J* = 1.2 Hz, 1 H, H-1^A), 4.97 (d, *J* = 11.6 Hz, 1 H, OCH_2Ar), 4.92 (d, *J* = 12.1 Hz, 1 H, OCH_2Ar), 4.90 (d, *J* = 1.6 Hz, 1 H, H-1^A), 4.71 (d, *J* = 9.4 Hz, 1 H, OCH_2Ar), 4.69 (d, *J* = 10.1 Hz, 1 H, OCH_2Ar), 4.63 (d, *J* = 11.6 Hz, 1 H, OCH_2Ar), 4.62-4.53 (m, 5 H, H-$6a^B$, H-2^B, H-5^B, $OC\mathit{H_2}Ar$), 4.51 (dd, *J* = 2.6, 1.8 Hz, 1 H, H-2^C), 4.48-4.25 (m, 15 H, H-1^G, H-$6b^C$, $OC\mathit{H_2}Ar$, H-5^C, H-$6a^B$, H-$6b^B$, H-5^E, H-3^A, H-4^E, H-5^D, H-5^F, H-5^G, H-$6a^G$, H-$6b^G$), 4.17 (dd, *J* = 12.3, 3.3 Hz, 1 H, H-$6a^F$), 4.12 (dd, *J* = 12.3, 3.2 Hz, 1 H, H-$6b^F$), 4.10 (dd, *J* = 9.4, 3.0 Hz, 1 H, H-3^E), 4.05-4.02 (m, 4 H, H-$6a^D$, H-$6b^D$, H-2^A, H-5^A), 4.00 (ddd, *J* = 1.6, 4.6, 9.8 Hz, 1 H, H-5^A), 3.90-3.86 (m, 2 H, H-$6a^A$, H-$6a^E$), 3.83-3.77 (m, 4 H, H-$6b^A$, H-$6b^E$, H-5^E, $OC\mathit{H_2}CH_2CH_2N_3$), 3.50 (ddd, *J* = 9.9, 6.2, 6.0 Hz, 1 H, $OC\mathit{H_2}CH_2$), 3.38-3.30 (m, 2 H, $CH_2C\mathit{H_2}N_3$), 2.02 (s, 3 H, $CH_3C{=}O$), 2.00 (s, 3 H, $CH_3C{=}O$), 1.86-1.81 (m, 2 H, 2 × $OCH_2C\mathit{H_2}CH_2N_3$); ^{13}C NMR (150 MHz, $CDCl_3$): δ = 169.0 (2 C, $CH_3C{=}O$), 166.3, 166.2, 166.1, 165.9, 165.6, 165.3, 165.2, 165.1, 165.0, 164.9 (16 C, ArC=O), 138.4-127.1 (120 C, Ar), 101.5 (C-1^F), 101.4 (C-1^B), 99.5 (C-1^C), 99.2 (2 C, C-1^D, C-1^G), 97.8 (C-1^E), 96.8 (C-1^A), 81.5 (C-3^A), 78.2 (C-2^A), 77.6 (C-2^B), 77.4 (C-3^E), 77.2 (C-4^E), 76.6 (C-2^C), 75.3 (OCH_2Ar), 74.5 (C-4^A), 73.3 (OCH_2Ar), 72.0 (OCH_2Ar), 71.6 (C-5^E), 71.5 (C-3^B), 71.1 (C-5^A), 70.7 (OCH_2Ar), 70.7 (C-3^C), 70.4 (C-3^D), 70.4 (C-5’), 69.8, 69.7, 69.5 (3 C), 69.4 and 69.3 (C-3^D, C-3^F, C-3^G, C-2^D, C-2^G, C-5^B, C-5^C, C-5^G, C-5^F, C-5^D, C-6^E), 68.3 (C-2^E), 67.4 (C-4^F), 67.3, 67.0, 66.9 and 66.7 (C-4^B, C-4^C, C-4^D, C-4^G), 66.4 (C-6^A), 64.8 ($O\mathit{C}H_2CH_2$), 63.7 (2 C, C-6^B, C-6^C), 63.1 (C-6^G), 62.7 and 62.5 (C-6^F, C-6^D), 48.4 ($CH_2\mathit{C}H_2N_3$), 28.8 ($OCH_2\mathit{C}H_2CH_2N_3$), 20.6 ($CH_3C{=}O$), 20.5 ($CH_3C{=}O$); ESI-TOF HRMS: *m/z* $C_{189}H_{169}N_3O_{54}$ calcd for $[M+2NH_4^+]^{2+}$: 1691.0656; found: 1691.0673.

3-Amino-1-propyl α-D-mannopyranosyl-(1→2)-α-D-mannopyranosyl-(1→2)-α-D-mannopyranosyl-(1→3)–[α-D-mannopyranosyl-(1→2)-α-D-mannopyranosyl-(1→4)-α-D-mannopyranosyl-(1→6)-α-D-mannopyranoside (NIT70A)

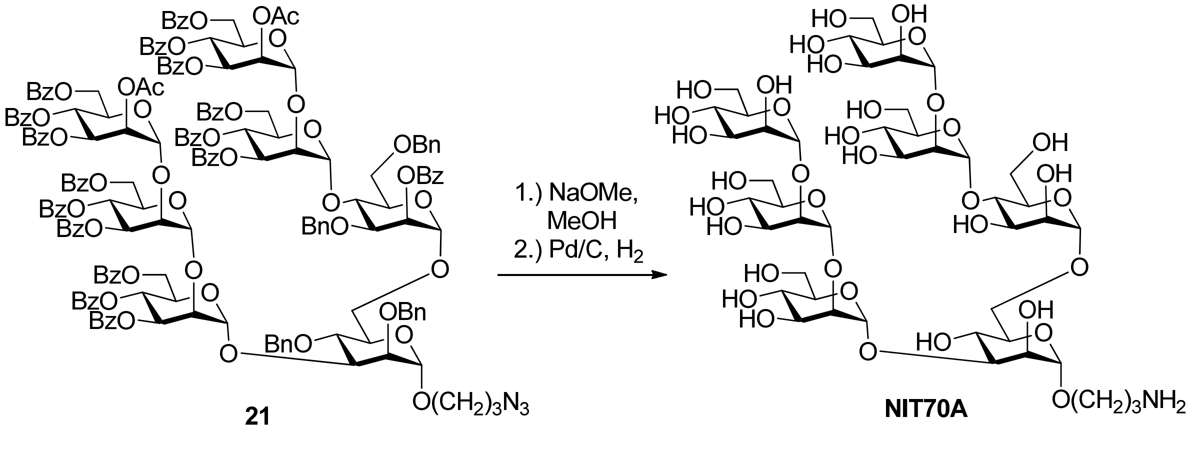

Deprotection was performed per general method A using **21** (62 mg; 0.019 mmol) in dry MeOH (2 ml) and 0.1 M NaOMe (1.1 ml; 0.111 mmol) for the deacylation and 10 % Pd/C (20 mg) for the hydrogenation. Workup and purification as described gave **NIT70A** as colourless amorphous solid (17 mg; 76 %); $[\alpha]_D^{21}$ +79 (*c* = 0.9, H_2O); ^1H, NMR (600 MHz, D_2O): δ = 5.45 (d, *J* = 1.7 Hz, 1 H, H-1^F), 5.32 (d, *J* = 1.6 Hz, 1 H, H-1^B), 5.27 (d, *J* = 1.6 Hz, 1 H, H-1^C), 5.01 and 5.00 (2 x d, *J* = 1.7 Hz, 1 H, H-1^G, H-1^D), 4.86 (d, *J* = 1.1 Hz, 1 H, H-1^E), 4.79 (d, *J* = 1.7 Hz, 1 H, H-1^A), 4.08-4.07 (m, 2 H, H-2^A, H-2^C), 4.06-4.05 (m, 2 H, H-2^B, H-2^F), 4.03-4.02 (m, 2 H, H-2^D, H-2^G), 3.97-3.59 (m, 38 H), 3.56 (ddd, *J* = 5.2, 6.9, 12.0 Hz, 1 H, OCH_2CH_2), 3.11-3.03 (m, 2 H, $CH_2CH_2NH_2$), 1.98-1.91 (m, 2 H, $CH_2CH_2NH_2$); ^{13}C NMR (150 MHz, D_2O): δ = 103.0 (2 C, C-1^D, C-1^G), 101.6 (C-1^B), 101.5 (C-1^C), 100.9 (C-1^F), 100.7 (C-1^A), 100.2 (C-1^E), 79.6, 79.5 (d.i.), 79.3 (C-3^A, C-2^B, C-2^F), 75.4 (C-4^E), 74.6 (C-5^A), 74.2, 74.1, 74.0 (6 C, C-5^B, C-5^C, C-5^D, C-5^E, C-5^F, C-5^G), 72.1, 72.0, 71.3, 71.2, 71.0, 70.9, 70.8, 70.5 (10 C, C-2^A, C-2^D, C-2^E, C-2^G, C-3^B, C-3^C, C-3^D, C-3^E, C-3^F, C-3^G), 67.9, 67.8, 67.7, 67.6 (5 C, C-4^B, C-4^C, C-4^D, C-4^F, C-4^G), 66.5 (C-4^A), 66.3 (C-6^A), 65.8 (OCH_2CH_2), 61.9, 61.8, 61.7 (6 C, C-6^B, C-6^C, C-6^D, C-6^E, C-6^F, C-6^G), 38.3 ($CH_2CH_2NH_2$), 27.9 ($CH_2CH_2NH_2$); ESI-TOF HRMS: *m/z* calcd for $C_{45}H_{79}NO_{36}$ $[M+H^+]^+$: 1210.4455; found: 1210.4455.

3-Azido-1-propyl 2-*O*-acetyl-3,4,6-tri-*O*-benzoyl-α-D-mannopyranosyl-(1→2)-3,4,6-tri-*O*-benzoyl-α-D-mannopyranosyl-(1→2)-3,4,6-tri-*O*-benzoyl-α-D-mannopyranosyl-(1→3)-[2-*O*-acetyl-3,4,6-tri-*O*-benzoyl-α-D-mannopyranosyl-(1→2)-3,4,6-tri-*O*-benzoyl-α-D-mannopyranosyl-(1→4)-2-*O*-benzoyl-3,6-di-*O*-benzyl-α-D-mannopyranosyl-(1→6)]-2,4-di-*O*-benzyl-β-D-mannopyranoside (22)

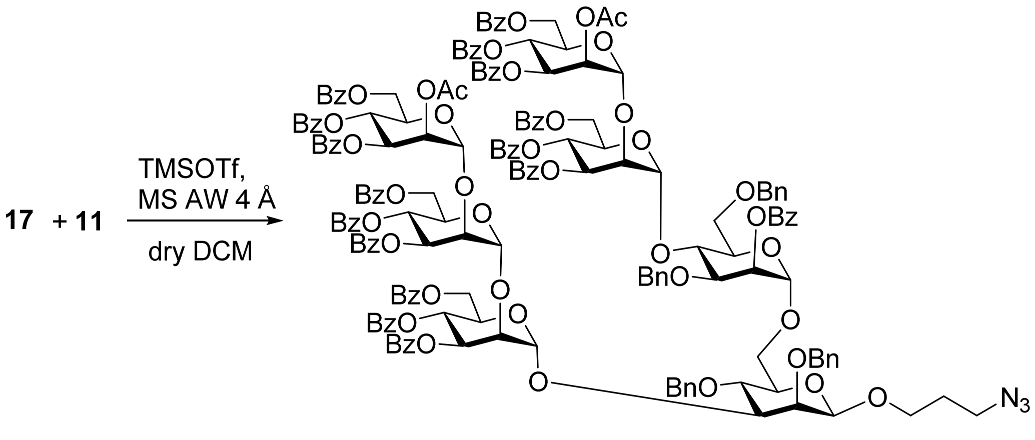

22

A suspension of **17** (68 mg; 0.029 mmol), **11** (50 mg; 0.043 mmol; 1.5 eq.) and AW MS 4 Å in dry DCM (1 ml) was stirred for 20 min at RT under Ar. Then a 0.29 M solution of TMSOTf in dry

DCM (10 µl) was added and the reaction mixture was stirred at RT for 2 h. The reaction was quenched *via* the addition of 3 drops of NEt_3, the suspension was filtered over Celite® and the filtrate was concentrated *in vacuo*. The crude product was purified *via* silica flash chromatography (hexane/EtOAc = 3/1 → 1/1) to give **22** (85 mg; 88%) as colorless foam; $\alpha]_D^{21}$ +12.9 (*c* = 1.1, $CHCl_3$); ^{1}H NMR (600 MHz, $CDCl_3$): δ = 8.05-8.00 (m, 32 H, Ar), 7.60-7.92 (m, 68 H, Ar), 6.05 (t, *J* = 9.8 Hz, 1 H, H-4^B), 6.00 (t, *J* = 9.7 Hz, 1 H, H-4^C), 5.97 (t, *J* = 9.8 Hz, 1 H, H-4^F), 5.97 (dd, *J* = 10.0, 3.2 Hz, 1 H, H-3^B), 5.92 (dd, *J* = 9.8, 3.1 Hz, 1 H, H-3^C), 5.86-5.79 (m, 4 H, H-4^D, H-4^G, H-3^D, H-3^F), 5.77 (dd, *J* = 10.0, 3.2 Hz, 1 H, H-3^G), 5.66 (app t, *J* = 2.2 Hz, 1 H, H-2^E), 5.62 (app t, *J* = 2.3 Hz, 1 H, H-2^D), 5.54 (d, *J* = 1.4 Hz, 1 H, H-1^B), 5.49 (dd, *J* = 3.0, 2.1 Hz, 1 H, H-2^G), 5.40 (d, *J* = 1.7 Hz, 1 H, H-1^F), 5.27 (br s, 1 H, H-1^C), 5.25 (d, 1 H, OCH_2Ar), 5.05 (d, *J* = 1.7 Hz, 1 H, H-1^E), 4.93 (d, *J* = 11.8 Hz, 1 H, OCH_2Ar), 4.91 (br s, 1 H, H-1^D), 4.82 (d, *J* = 12.7 Hz, 1 H, OCH_2Ar), 4.72 (d, *J* = 11.2 Hz, 1 H, OCH_2Ar), 4.61-4.54 (m, 4 H, 3 x OCH_2Ar, H-6a^B), 4.54 (dd, 1 H, H-2^B), 4.50-4.36 (m, 12 H, H-1^G, H-1^A, H-5^B, H-5^C, H-2^C, H-5^F, H-6a^C, H-6b^C, H-6b^B, H-6a^G, H-6b^G, 1 x OCH_2Ar), 4.31-4.22 (m, 4 H, H-5^G, H-2^F, H-4^E, H-5^D), 4.17 (dd, *J* = 12.3, 3.3 Hz, 1 H, H-6a^F), 4.12 (dd, *J* = 12.3, 3.2 Hz, 1 H, H-6b^F), 4.08 (dd, *J* = 9.4, 3.0 Hz, 1 H, H-3^E), 4.04 (br d, *J* = 2.9 Hz, 1 H, H-2^A), 4.04-3.96 (m, 4 H, H-6a^D, H-6b^D, H-5^E, OCH_2CH_2), 3.94 (t, *J* = 9.7 Hz, 1 H, H-4^A), 3.90 (dd, *J* = 4.8, 11.1 Hz, H-6a^E), 3.85 (dd, *J* = 6.1, 11.4 Hz, 1 H, H-6a^A), 3.82-3.79 (m, 2 H, H-3^A, H-6b^E), 3.74 (dd, *J* = 2.1,11.3 Hz, H-6b^A), 3.60 (ddd, *J* = 9.7, 5.2, 7.1 Hz, 1 H, OCH_2CH_2), 3.43-3.31 (m, 3 H, H-5^A, $CH_2CH_2N_3$), 2.01 (s, 3 H, CH_3C=O), 2.00 (s, 3 H, CH_3C=O), 1.98-1.89 (m, 2 H, 2 × $OCH_2CH_2CH_2N_3$); ^{13}C NMR (150 MHz, $CDCl_3$): δ = 169.0 (2 C, CH_3C=O), 166.3, 166.2, 166.1, 165.6, 165.5, 165.4, 165.2, 165.1, 164.9 (16 C, ArC=O), 138.9-127.2 (120 C, Ar), 101.6 (C-1^A), 101.5 (C-1^F), 101.3 (C-1^B), 99.3 (C-1^D), 99.2 (C-1^G), 97.9 (C-1^E), 83.0 (C-3^A), 78.3 (C-2^A), 77.9 (C-2^B), 77.5 (C-3^F), 77.1 (C-3^E), 76.7 (C-4^E), 76.2 (C-2^C), 75.2 (OCH_2Ar), 74.7 (2 C, C-5^A, C-4^A), 74.2 (OCH_2Ar), 73.2 (OCH_2Ar), 71.1 (C-3^B), 71.0 (C-5^E), 70.7 (2 C, C-3^C, OCH_2Ar), 70.4 (C-3^D), 68.8 and 69.7 (3 C, C-5^B, C-5^F, C-3^G), 69.4 (C-3^F, C-2^D, C-2^G, C-5^C, C-5^G), 69.3 (C-6^E), 69.2 (C-5^D), 68.2 (C-2^E), 67.3 and 67.1 (C-4^B, C-4^C, C-4^F), 66.8 and 66.7 (C-6^A, OCH_2CH_2), 66.6 (C-4^D, C-4^G), 63.7 and 63.6 (3 C, C-6^B, C-6^G, C-6^C), 62.8 (C-6^D), 62.4 C-6^F), 48.5 ($CH_2CH_2N_3$), 29.4 ($OCH_2CH_2CH_2N_3$), 20.6 (CH_3C=O), 20.5 (CH_3C=O); ESI-TOF HRMS: *m/z* calcd for $C_{189}H_{169}N_3O_{54}$ $[M-N_3+NH_4^+H^+]^{2+}$: 1662.0540; found: 1662.0548.

3-Amino-1-propyl α-D-mannopyranosyl-(1→2)-α-D-mannopyranosyl-(1→2)-α-D-mannopyranosyl-(1→3)–[α-D-mannopyranosyl-(1→2)-α-D-mannopyranosyl-(1→4)-α-D-mannopyranosyl-(1→6)-β-D-mannopyranoside (NIT70B)

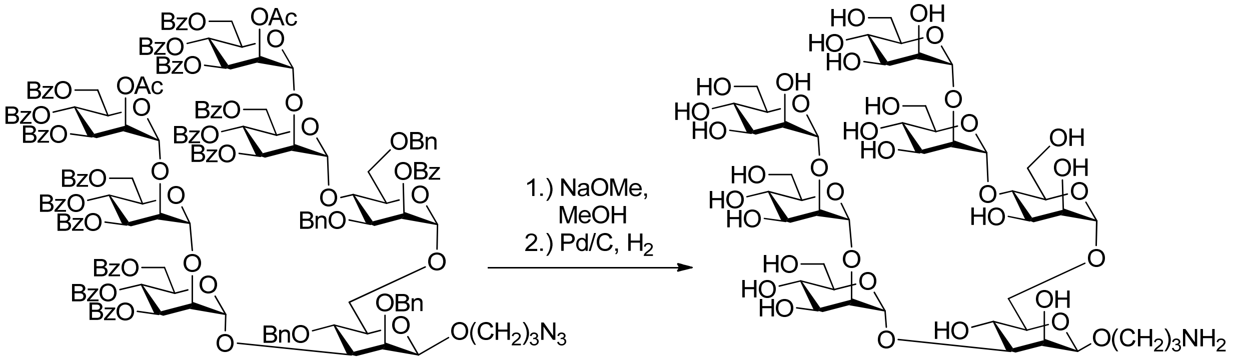

22 **NIT70B**

A solution of **22** (55 mg, 0.016 mml) in dry MeOH (2 ml) was stirred with 0.1 M sodium methoxide (1.0 ml) for 48 h at RT under Ar. DOWEX-50WX8 resin (H^+-form) was then added to give pH = 7. The resin was filtered off and the filtrate was co-evaporated with toluene in order to remove methyl benzoate. The crude product was dissolved in MeOH/H_2O/AcOH (1/1/0.05; 2 m1), 10% Pd/C (17 mg) was added and the suspension was stirred under hydrogen atmosphere for 48 h at RT. The catalyst was removed by filtration over Celite® and the filtrate was purified by gel chromatography (Sephadex LH-20; water/MeOH = 2/1). Product containing fractions were lyophilized to give amine **NIT70B** as colourless amorphous solid (13 mg, 65%); $[\alpha]_D^{21}$ +33.9 (*c* 1.1, H_2O); ^1H NMR (600 MHz, D_2O): δ = 5.46 (d, *J* = 1.4 Hz, 1 H, H-1^F), 5.31 (d, *J* = 1.3 Hz, 1 H, H-1^B), 5.27 (d, *J* = 1.5 Hz, 1 H, H-1^C), 5.01 and 5.00 (2 x d, *J* = 1.6 Hz, 1 H, H-1^G, H-1^D), 4.87 (br s, 1 H, H-1^E), 4.64 (br s, 1 H, H-1^A), 4.10 (br. d, *J* = 3.1 Hz, 1 H, H-2^A), 4.07 (dd , *J* = 3.1, 1.9 Hz 1 H, H-2^C), 4.06-4.05 (m, 2 H, H-2^B, H-2^F), 4.03-4.02 (m, 2 H, H-2^D, H-2^G), 3.97- 3.58 (m, 37 H), 3.50 (ddd, *J* = 1.9, 5.2, 9.9 Hz, 1 H, H-5^A), 3.56 (ddd, *J* = 5.2, 6.9, 12.0 Hz, 1 H, OCH_2CH_2), 3.16-3.08 (m, 2 H, $CH_2CH_2NH_2$), 2.02-1.91 (m, 2 H, $CH_2CH_2NH_2$); ^{13}C NMR (150 MHz, D_2O): δ = 103.0 (2 C, C-1^D, C-1^G), 101.5 (C-1^B), 101.4 (C-1^C), 100.8 (C-1^F), 100.6 (C-1^A), 100.2 (C-1^E), 81.6 (C-3^A), 79.5, 79.4 (C-2^B, C-2^F), 79.3 (C-2^C), 75.3 (C-4^E), 75.0 (C-5^A), 74.6, 74.2, 74.1, 74.0 (6 C, C-5^B, C-5^C, C-5^D, C-5^E, C-5^F, C-5^G), 72.1, 71.3, 71.2, 71.0, 70.9, 70.8, (10 C, C-2^A, C-2^D, C-2^E, C-2^G, C-3^B, C-3^C, C-3^D, C-3^E, C-3^F, C-3^G), 68.1 (OCH_2CH_2), 67.8, 67.7, 67.6, 67.5 (5 C, C-4^B, C-4^C, C-4^D, C-4^F, C-4^G), 66.6 (C-4^A), 66.4 (C-6^A), 61.9, 61.8, 61.7 (6 C, C-6^B, C-6^C, C-6^D, C-6^E, C-6^F, C-6^G), 38.6 ($CH_2CH_2NH_2$), 27.6 ($CH_2CH_2NH_2$); ESI-TOF HRMS: *m/z* calcd for $C_{45}H_{79}NO_{36}$ $[M+H^+]^+$: 1210.4455; found: 1210.4472.

3-Azido-1-propyl 2-*O*-acetyl-3,4,6-tri-*O*-benzoyl-α-D-mannopyranosyl-(1→2)-3,4,6-tri-*O*-benzoyl-α-D-mannopyranosyl-(1→2)-3,4,6-tri-*O*-benzoyl-α-D-mannopyranosyl-(1→3)-[2-*O*-acetyl-3,4,6-tri-*O*-benzoyl-α-D-mannopyranosyl-(1→2)-3,4,6-tri-*O*-benzoyl-α-D-

mannopyranosyl-(1→6)-2-*O*-benzoyl-3,4-di-*O*-benzyl-α-D-mannopyranosyl-(1→6)]-2,4-di-*O*-benzyl-α-D-mannopyranoside (**23**)

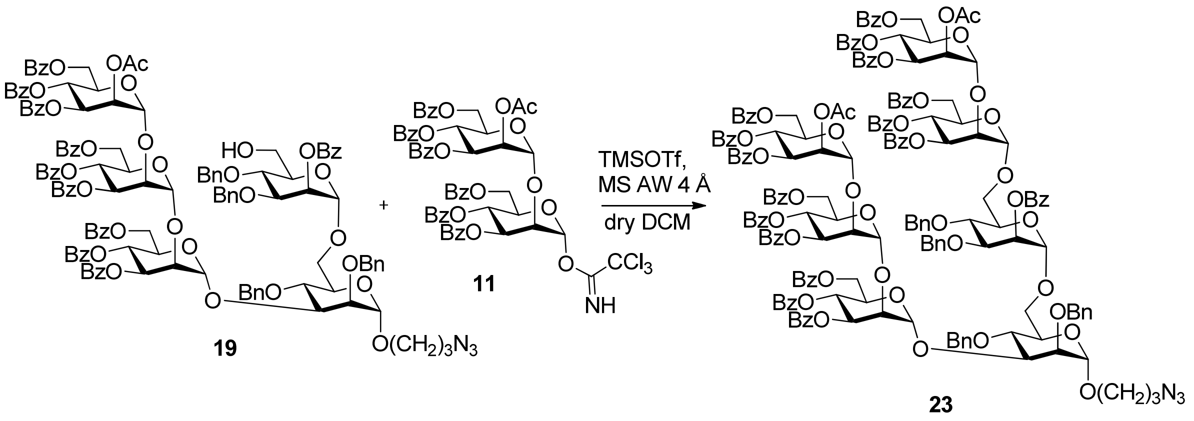

A suspension of **19** (33 mg; 0.014 mmol), **11** (24 mg; 0.021 mmol; 1.5 eq.) and AW MS 4 Å in dry DCM (1 ml) under Ar was stirred for 20 min at RT. A solution of 0.1 M TMSOTf (10 µl, 0.1 eq.) in dry DCM was added and the reaction mixture was stirred at RT for 3 h. The reaction was quenched by the addition of 3 drops of NEt_3. The suspension was filtered over Celite® and the solvent was removed *in vacuo*. The crude product was purified by flash chromatography (toluene/EtOAc = 15/1 →10/1) to give **23** (31 mg; 66 %) as colorless syrup; $[\alpha]_D^{21}$ +30.5 (*c* 1.2, $CHCl_3$); ^{1}H NMR (600 MHz, $CDCl_3$): δ = 8.08-7.74 (m, 31 H, Ar), 7.53-6.95 (m, 69 H, Ar), 6.08 (t, *J* = 9.8 Hz, 1 H, H-4^C), 6.06 (t, *J* = 9.9 Hz, 1 H, H-4^B), 5.99 (t, *J* = 10.2 Hz, 1 H, H-4^F), 5.97 (dd, *J* = 9.8, 3.1 Hz, 1 H, H-3^B), 5.91 (dd, *J* = 10.2, 3.1 Hz, 1 H, H-3^C), 5.87-5.83 (m, 3 H, H-3^F, H-4^D, H-4^G), 5.81 (dd, *J* = 3.2, 9.9 Hz, H-3^G), 5.80 (dd, *J* = 3.2, 10.0 Hz, H-3^D), 5.72 (app t, *J* = 2.3 Hz, 1 H, H-2^E), 5.60 (dd, *J* = 2.8, 2.3 Hz, 1 H, H-2^D), 5.57 (dd, *J* = 3.1 Hz, 2.1 Hz, 1 H, H-2^G), 5.50 (d, *J* = 1.2 Hz, 1 H, H-1^B), 5.32 (d, *J* = 1.4 Hz, 1 H, H-1^C), 5.28 (d, *J* = 1.6 Hz, 1 H, H-1^F), 5.15 (d, *J* = 1.7 Hz, H-1^E), 5.12 (d, *J* = 11.6 Hz, 1 H, OCH_2Ar), 4.98 (d, *J* = 1.0 Hz, 1 H, H-1^A), 4.93 (d, J = 11.6 Hz, 1 H, OCH_2Ar), 4.92 (d, *J* = 12.2 Hz, 1 H, OCH_2Ar), 4.86 (d, *J* = 1.1 Hz, 1 H, H-1^D), 4.74 (d, *J* = 11.7 Hz, 1 H, OCH_2Ar), 4.69 (d, *J* = 9.6 Hz, 1 H, OCH_2Ar), 4.68 (s, 1 H, H-1^G), 4.67 (d, *J* = 12.2 Hz, OCH_2Ar), 4.57 (d, J = 11.6 Hz, $OC\mathit{H_2}Ar$), 4.55-4.51 (m, 3 H, H-2^B, H-5^B, H-6a^B), 4.49-4.41 (m, 7 H, H-2^C, H-5^F, H-5^C, H-6b^B, H-6a^C, H-6b^C, $OC\mathit{H_2}Ar$), 4.39-4.32 (m, 4 H, H-6a^F, H-6b^F, H-6a^G, H-6b^G), 4.29 (dd, *J* = 1.7, 3.2 Hz, H-2^F), 4.28-4.23 (m, 3 H, H-3^A, H-5^D, H-5^G), 4.13 (t, *J* = 9.3 Hz, 1 H, H-4^A), 4.12-4.08 (m, 2 H, H-3^E, H-4^E), 4.05-3.99 (m, 3 H, H-2^A, H-6a^D, H-6b^D), 3.94 (dd, *J* = 11.5, 3.1 Hz, 1 H, H-6a^A), 3.85 (dd, *J* = 11.6, 4.3 Hz, 1 H, H-6a^E), 3.29 (ddd, *J* = 8.9, 2.5, 2.1 Hz, 1 H, H-5^A), 3.73-3.66 (m, 3 H, H-5^E, H-6b^E, $OC\mathit{H_2}CH_2$), 3.58 (dd, *J* = 11.5, 0.9 Hz, 1 H, H-6b^A), 3.39 (dt, *J* = 10.1, 6.1, 6.1 Hz, 1 H, $OC\mathit{H_2}CH_2$), 3.27-3.19 (m, 2 H, $CH_2C\mathit{H_2}N_3$), 2.02

(s, 3 H, $CH_3C=O$), 2.00 (s, 3 H, $CH_3C=O$), 1.73-1.68 (m, 2 H, $OCH_2CH_2CH_2N_3$); ^{13}C NMR (150 MHz, $CDCl_3$): δ = 169.0 (2 C, $CH_3C=O$), 166.3 166.2, 165.8, 165.6, 165.5, 165.4, 165.3, 165.1, 164.9 (16 C, ArC=O), 138.8-127.3 (120 C, Ar), 101.4 (C-1^B), 100.7 (C-1^C), 100.0 (C-1^G), 99.5 (C-1^D), 99.0 (C-1^F), 98.4 (C-1^E), 96.7 (C-1^A), 81.2 (C-3^A), 78.3 (C-2^A), 78.0 (C-2^F), 77.7 (C-3^E), 77.5 (C-2^B), 76.7 (C-2^C), 75.3 (OCH_2Ar), 75.0 (OCH_2Ar), 74.4 (C-4^E), 73.8 (C-4^A), 71.9 (OCH_2Ar), 71.6 (C-5^E), 71.3 (C-3^B), 71.2 (OCH_2Ar), 71.0 (C-5^A), 70.8 (C-3^F, C-3^C), 69.8, 69.6, 69.5 (6 C, C-3^D, C-3^G, C-2^D, C-2^G, C-5^C, C-5^G or C-5^D), 69.4 and 69.3 (C-5^B, C-5^F), 68.8. and 68.7 (C-5^G or C-5^D, C-2^E), 67.4 (C-4^F), 67.3 and 67.0 (C-4^C, C-4^B), 66.9 and 66.8 (C-4^G, C-4^D), 66.6 (C-6^E), 65.9 (C-6^A), 64.6 (OCH_2CH_2), 63.7 (C-6^B), 63.4, 63.1 and 62.9 (C-6^C, C-6^F, C-6^G), 62.5 (C-6^D), 48.3 ($CH_2CH_2N_3$), 28.8 ($OCH_2CH_2CH_2N_3$), 20.6 (2 C, $CH_3C=O$). ESI-TOF HRMS: *m/z* $C_{189}H_{169}N_3O_{54}$ calcd for $[M+ 2 NH_4^+]^{2+}$: 1691.0656; found: 1691.0717.

3-Amino-1-propyl α-D-mannopyranosyl-(1→2)-α-D-mannopyranosyl-(1→2)-α-D-mannopyranosyl-(1→3)–[α-D-mannopyranosyl-(1→2)-α-D-mannopyranosyl-(1→6)-α-D-mannopyranosyl-(1→6)-α-D-mannopyranoside (NIT68A)

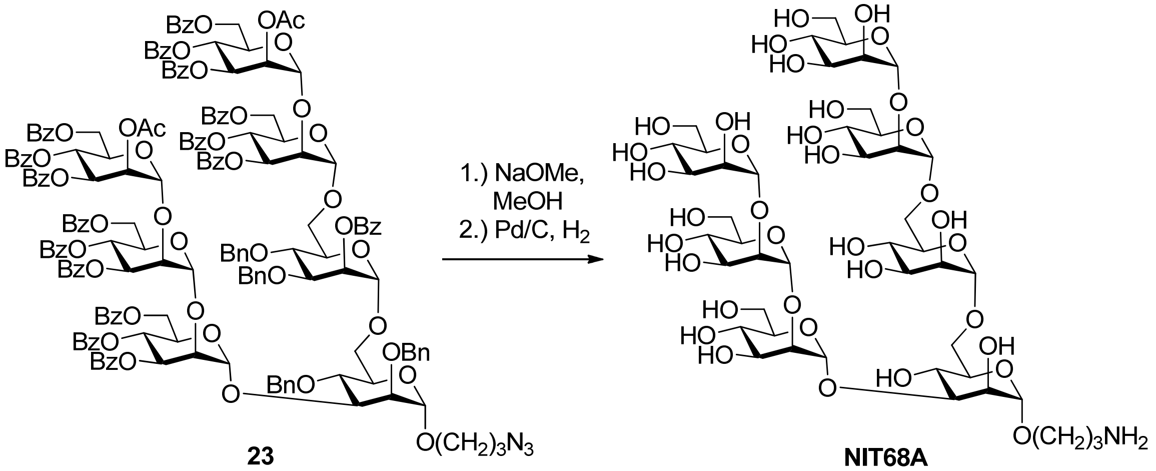

Deprotection was performed according to general method A using **23** (58 mg; 0.017 mmol) in dry MeOH (2 ml) and 0.1 M NaOMe (1 ml) for the deacylation and 10 % Pd/C (18 mg) for the hydrogenation. Workup and purification as described gave **NIT68A** as colourless amorphous solid (20 mg; 95 %); $[\alpha]_D^{21}$ +83.1 (*c* = 0.8, H_2O); ^{1}H NMR (600 MHz, D_2O): δ = 5.31 (d, *J* = 1.5 Hz, 1 H, H-1^B), 5.26 (d, *J* = 2.1 Hz, 1 H, H-1^C), 5.10 (d, *J* = 1.5 Hz, 1 H, H-1^F), 5.00 (d, *J* = 1.8 Hz, 1 H, H-1^D), 4.99 (d, *J* = 1.9 Hz, 1 H, H-1^G), 4.85 (d, *J* = 1.5 Hz, 1 H, H-1^E), 4.78 (d, *J* = 1.8 Hz, 1 H, H-1^A), 4.07-4.06 (m, 2 H, H-2^A, H-2^C), 4.04-4.02 (m, 3 H, H-2^B, H-2^D, H-2^G), 3.97 (dd, *J* = 1.7, 3.4 Hz, 1 H, H-2^F), 3.96-3.62 (m, 35 H), 3.60 and 3.58 (2 x t, *J* = 9.7 Hz, each 1 H, H-4^D, H-4^G), 3.56-3.52 (m, 1 H, OCH_2CH_2), 3.10-3.00 (m, 2 H, $CH_2CH_2NH_2$), 1.98-1.90 (m, 2 H,

$CH_2CH_2NH_2$); ^{13}C NMR (150 MHz, D_2O): δ = 103.1 and 103.0 (C-1^D, C-1^G), 101.5 (2 C, C-1^B, C-1^C), 100.7 (C-1^A), 100.4 (C-1^E), 99.0 (C-1^F), 79.7, 79.6, 79.5, 79.3 (C-3^A, C-2^B, C-2^C, C-2^F), 74.2, 74.1 (3 C), 74.0 (C-5^B, C-5^C, C-5^F, C-5^G, C-5^D), 73.6 (C-5^E), 72.0, 71.9, 71.7, 71.2 (2 C), 71.1, 70.9, 70.8 (2 C), 70.5 (C-2^A, C-2^D, C-2^E, C-2^G, C-3^D, C-3^B, C-3^C, C-3^E, C-3^F, C-3^G), 67.8, 67.7 and 67.3 (6 C, C-4^B, C-4^C, C-4^D, C-4^E, C-4^F, C-4^G), 67.7 (C-6^E), 66.5 (C-4^A), 65.9 (C-6^A) 65.8 (O*C*H_2), 62.3, 62.0, 61.9 (2 C) and 61.8 (C-6^B, C-6^C, C-6^D, C-6^F, C-6^G), 38.3 ($CH_2CH_2NH_2$), 27.9 ($CH_2CH_2NH_2$). ESI-TOF HRMS: *m/z* calcd for $C_{45}H_{79}NO_{36}$ $[M+H^+]^+$: 1210.4455; found: 1210.4462.

3-Azido-1-propyl 2-*O*-acetyl-3,4,6-tri-*O*-benzoyl-α-D-mannopyranosyl-(1→2)-3,4,6-tri-*O*-benzoyl-α-D-mannopyranosyl-(1→2)-3,4,6-tri-*O*-benzoyl-α-D-mannopyranosyl-(1→3)-[2-*O*-acetyl-3,4,6-tri-*O*-benzoyl-α-D-mannopyranosyl-(1→2)-3,4,6-tri-*O*-benzoyl-α-D-mannopyranosyl-(1→6)-2-*O*-benzoyl-3,4-di-*O*-benzyl-α-D-mannopyranosyl-(1→6)]-2,4-di-*O*-benzyl-β-D-mannopyranoside (24)

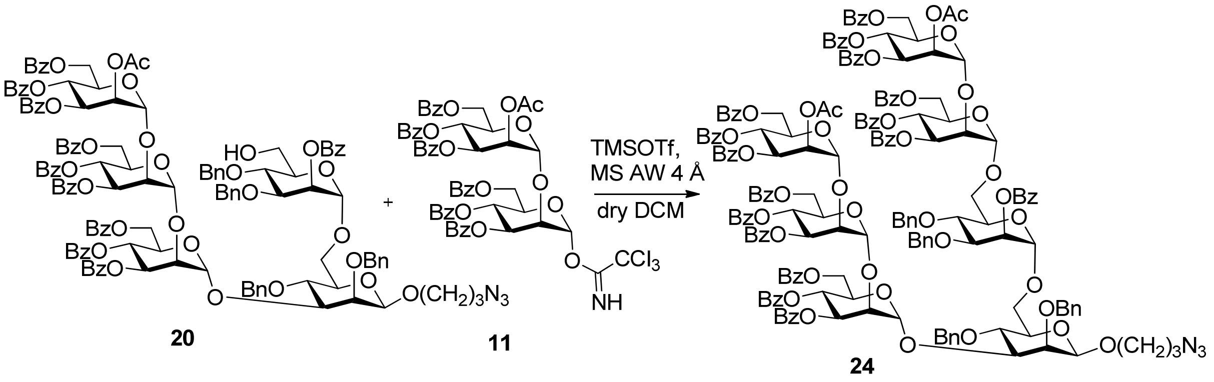

A suspension of **20** (48 mg; 0.020 mmol), **11** (35 mg; 0.031 mmol; 1.5 eq) and AW MS 4 Å in dry DCM (1 ml) under Argon was stirred for 20 min at RT. Then a solution of 0.2 M TMSOTf (10 µl; 0.1 eq) in dry DCM was added and the reaction mixture was stirred at RT for 3 h. The reaction was quenched by the addition of 3 drops of NEt_3. The mixture was filtered over Celite® and the filtrate was concentrated. The crude product was purified by flash chromatography (toluene/EtOAc = 15/1 → 10/1) to afford **24** (48 mg; 70 %) as colorless amorphous solid; $[\alpha]_D^{21}$ +17.5 (*c* 1.1, $CHCl_3$); ^1H NMR (600 MHz, $CDCl_3$): δ = 8.10-7.74 (m, 23 H, Ar), 7.60-7.01 (m, 77 H, Ar), 6.03 (t, *J* = 9.9 Hz, 1 H, H-4^C), 6.00 (t, *J* = 9.9 Hz, 1 H, H-4^B), 5.99 (t, *J* = 10.6 Hz, 1 H, H-4^G), 5.94 (dd, *J* = 9.9, 3.2 Hz, 1 H, H-3^C), 5.89-5.84 (m, 2 H, H-3^F, H-4^F), 5.83-5.78 (m, 3 H, H-3^G, H-3^D, H-4^D), 5.69 (app t, *J* = 2.6, H-2^E), 5.60 (app t, *J* = 2.4 Hz, 1 H, H-2^D), 5.55 (dd, *J* = 3.1, 2.2 Hz, 1 H, H-2^G), 5.50 (d, *J* = 1.5 Hz, 1 H, H-1^B), 5.29 (d, *J* = 1.4 Hz, 1 H, H-1^F), 5.22 (d, *J* = 12.8 Hz, 1 H, OCH_2Ar), 5.21 (d, *J* = 1.3 Hz, 1 H, H-1^C), 5.10 (d, *J* = 11.6 Hz, 1 H, OCH_2Ar), 5.06 (d, *J*

= 1.6 Hz, 1 H, H-1^{E}), 4.88 (d, *J* = 11.3 Hz, 1 H, OCH_2Ar), 4.87 (br s, 1 H, H-1^{D}), 4.78 (d, *J* = 12.8 Hz, 1 H, OCH_2Ar), 4.73 (d, *J* = 11.6 Hz, 1 H, OCH_2Ar), 4.71 (d, *J* = 11.5 Hz, 1 H, OCH_2Ar), 4.67 (d, *J* = 1.8 Hz, 1 H, H-1^{G}), 4.55-4.32 (m, 15 H, H-2^{B}, H-2^{C}, H-5^{B}, H-5^{C}, H-5^{F}, H-6a^{B}, H-6b^{B}, H-6a^{C}, H-6b^{C}, H-6a^{F}, H-6b^{F}, H-6a^{G}, H-6b^{G}, OCH_2Ar), 4.32 (br s, 1 H, H-1^{A}), 4.29 (dd, *J* = 3.2, 1.4 Hz, 1 H, H-2^{F}), 4.25 (ddd, *J* = 9.9, 4.1, 3.6 Hz, 1 H, H-5^{G}), 4.21 (dt, *J* = 8.5, 4.1 Hz, 1 H, H-5^{D}), 4.13 (t, *J* = 9.5 Hz, 1 H, H-4^{E}), 4.08 (dd, *J* = 9.5, 3.0 Hz, 1 H, H-3^{E}), 4.02 (br d, *J* = 2.9 Hz, H-2^{A}), 4.02-3.98 (m, 2 H, H-6a^{D}, H-6b^{D}), 3.97 (t, *J* = 9.6 Hz, 1 H, H-4^{A}), 3.93 (dd, *J* = 11.6, 2.9 Hz, 1 H, H-6a^{A}), 3.87-3.77 (m, H-6a^{E}, H-5^{E}, H-3^{A}, $OC\mathit{H_2}CH_2$), 3.68 (br d, *J* = 9.5 Hz, 1 H, H-6b^{E}), 3.57 (br d, *J* = 10.2 Hz, 1 H, H-6b^{A}), 3.45 (ddd, *J* = 9.8, 5.3, 7.1 Hz, 1 H, $OC\mathit{H_2}CH_2$), 3.32-3.22 (m, 3 H, H-5^{A}, $CH_2C\mathit{H_2}N_3$), 2.00 (s, 3 H, $CH_3C{=}O$), 1.99 (s, 3 H, $CH_3C{=}O$) , 1.84-1.72 (m, 2 H, $OCH_2CH_2CH_2N_3$); ^{13}C NMR (150 MHz, $CDCl_3$): δ = 169.1, 169.0 ($CH_3C{=}O$), 166.3, 166.2, 166.1, 165.9, 165.6, 165.3, 165.2, 165.1, 165.0, 164.9 (16 C, ArC=O), 139.1-126.1 (120 C, Ar), 101.6 (C-1^{A}), 101.3 (C-1^{B}), 100.9 (C-1^{C}), 100.0 (C-1^{G}), 99.4 (C-1^{D}), 99.0 (C-1^{F}), 98.0 (C-1^{E}), 83.3 (C-3^{A}), 78.4 (C-2^{A}), 78.2 (C-2^{B}), 78.0 (C-2^{F}), 77.7 (C-3^{F}), 76.3 (C-2^{C}), 75.1 ($O\mathit{C}H_2Ar$), 75.0 ($O\mathit{C}H_2Ar$), 74.8 (C-5^{A}), 74.6 (C-4^{A}), 74.2 ($O\mathit{C}H_2Ar$), 73.7 (C-4^{E}), 71.2 and 71.1 (2 C, C-5^{E}, C-3^{C}), 71.0 ($O\mathit{C}H_2Ar$), 70.7 (2 C, C-3^{B}, C-3^{F}), 69.8, 69.7, 69.5, 69.4 and 69.2 (8 C, C-2^{D}, C-2^{G}, C-3^{D}, C-3^{G}, C-5^{B}, C-5^{C}, C-5^{F}, C-5^{D}), 68.7 and 68.6 (C-2^{E}, C-5^{G}), 67.3. and 67.1 (3 C, C-4^{B}, C-4^{C}, C-4^{G}), 66.8 (C-4^{F}, C-4^{D}), 66.8 ($O\mathit{C}H_2CH_2$), 66.7 (C-6^{E}), 65.9 (C-6^{A}), 63.7 (C-6'), 63.5, 63.4, 62.9 (C-6^{C}, C-6^{G}, C-6^{F}), 62.7 (C-6^{D}), 48.4 ($CH_2\mathit{C}H_2N_3$), 29.3 ($OCH_2\mathit{C}H_2CH_2N_3$), 20.6, ($CH_3C{=}O$), 20.5 ($CH_3C{=}O$). ESI-TOF HRMS: *m/z* $C_{189}H_{169}N_3O_{54}$ calcd for $[M + 2NH_4^+]^{2+}$: 1690.0623; found: 1690.0599.

3-Amino-1-propyl α-D-mannopyranosyl-(1→2)-α-D-mannopyranosyl-(1→2)-α-D-mannopyranosyl-(1→3)–[α-D-mannopyranosyl-(1→2)-α-D-mannopyranosyl-(1→6)-α-D-mannopyranosyl-(1→6)-β-D-mannopyranoside (NIT68B)

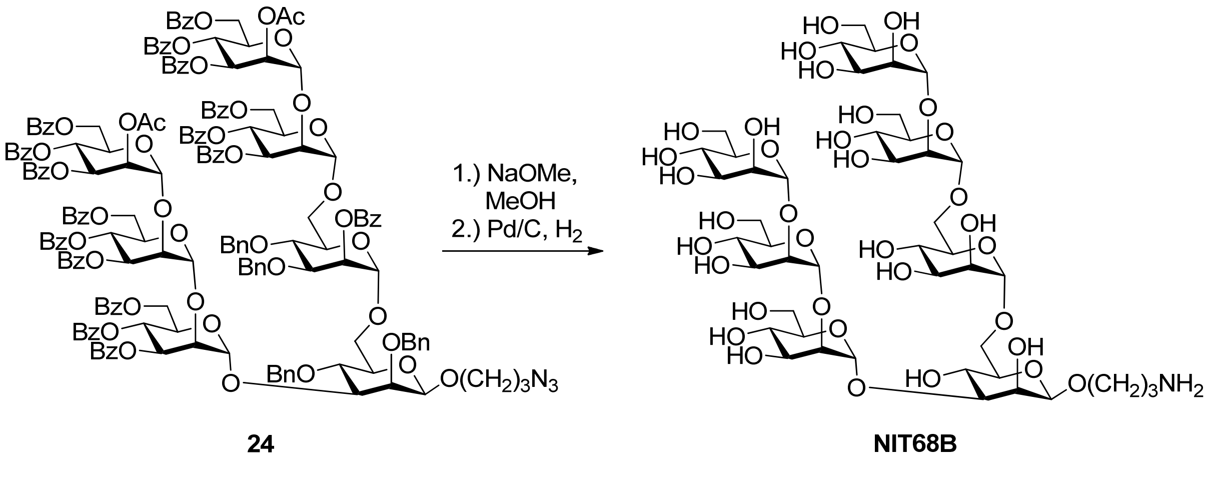

24 **NIT68B**

Deprotection was performed according to general method A using **24** (38 mg; 0.011 mmol) in dry MeOH (2 ml) and 0.1 M NaOMe (0.7 ml) for the deacylation and 10% Pd/C (11 mg) for the hydrogenation. Workup and purification as described gave **NIT68B** as colourless amorphous solid (13 mg; 95 %); $[\alpha]_D^{21}$ +50.6 (*c* 0.7, H_2O); ^{1}H NMR (600 MHz, D_2O): δ = 5.30 (d, *J* = 1.5 Hz, 1 H, H-1^B), 5.27 (d, *J* = 1.6 Hz, 1 H, H-1^C), 5.11 (d, *J* = 1.7 Hz, 1 H, H-1^F), 5.01 and 5.00 (2 x d, *J* = 1.8 Hz, each 1 H, H-1^D, H-1^G), 4.87 (d, *J* = 1.7 Hz, 1 H, H-1^E), 4.64 (s, 1 H, H-1^A), 4.11 (br d, *J* = 3.2 Hz, 1 H, H-2^A), 4.07 (dd, *J* = 1.8, 3.2 Hz, 1 H, H-2^C), 4.05 (dd, *J* = 1.8, 3.2 Hz, 1 H, H-2^B), 4.04 and 4.03 (2 x dd, *J* = 1.8, 3.3 Hz, each 1 H, H-2^D, H-2^G), 3.98 (dd, *J* = 1.8, 3.3 Hz, 1 H, H-2^F), 3.97-3.90 (m, 8 H, H-2^E, H-3^B, H-3^F, H-3^C, OCH_2CH_2, H-6a^A, H-6a^E, H-6b^E), 3.88-3.64 (m, 30 H), 3.60 and 3.59 (2 x t, *J* = 9.6 and 9.7 Hz, each 1 H, H-4^G, H-4^D), 3.52 (ddd, *J* = 2.0. 4.8, 9.9 Hz, 1 H, H-5^A), 3.11-3.06 (m, 2 H, $CH_2CH_2NH_2$), 1.98-1.91 (m, 2 H, $CH_2CH_2NH_2$); ^{13}C NMR (150 MHz, D_2O): δ = 103.1, 103.0 (C-1^D, C-1^G), 101.5, 101.4 (C-1^B, C-1^C), 100.8 (C-1^A), 100.3 (C-1^E), 99.0 (C-1^F), 81.7 (C-3^A), 79.5 (2 C, C-2^B, C-2^C), 79.3 (C-2^F), 74.9 (C-5^A), 74.2, 74.1, 74.0 (5 C, C-5^B, C-5^C, C-5^F, C-5^G, C-5^D), 73.6 (C-5^E), 71.9, 71.7, 71.2 (2 C), 71.1, 70.9 (2 C), 70.8 (2 C) and 70.7 (C-2^A, C-2^D, C-2^E, C-2^G, C-3^D, C-3^B, C-3^C, C-3^E, C-3^F, C-3^G), 68.2 (OCH_2CH_2), 67.8, 67.7 and 67.6 (6 C, C-4^B, C-4^C, C-4^D, C-4^E, C-4^F, C-4^G), 67.3 (C-4^A), 66.7 (C-6^E), 66.2 (C-6^A), 62.0, 61.9, 61.8 (5 C, C-6^B, C-6^C, C-6^D, C-6^G, C-6^F), 38.5 ($CH_2CH_2NH_2$), 27.9 ($CH_2CH_2NH_2$); ESI-TOF HRMS: *m/z* calcd for $C_{45}H_{79}NO_{36}$ $[M+H^+]^+$: 1210.4455; found: 1210.4444.

SUPPLEMENTARY REFERENCES

1 Patil, P. S., Lee, C. C., Huang, Y. W., Zulueta, M. M. & Hung, S. C. Regioselective and stereoselective benzylidene installation and one-pot protection of D-mannose. *Org Biomol Chem* **11**, 2605-2612, doi:10.1039/c3ob40079d (2013).

2 Tam, P. H. & Lowary, T. L. Synthesis of deoxy and methoxy analogs of octyl α-D-mannopyranosyl-(1→6)-α-D-mannopyranoside as probes for mycobacterial lipoarabinomannan biosynthesis. *Carbohydr Res* **342**, 1741-1772, doi:10.1016/j.carres.2007.05.001 (2007).

3 Wang, J., Li, H., Zou, G. & Wang, L. X. Novel template-assembled oligosaccharide clusters as epitope mimics for HIV-neutralizing antibody 2G12. Design, synthesis, and antibody binding study. *Org Biomol Chem* **5**, 1529-1540 (2007).

4 Bailey, J. J. & Bundle, D. R. Synthesis of high-mannose 1-thio glycans and their conjugation to protein. *Org Biomol Chem* **12**, 2193-2213, doi:10.1039/c3ob42194e (2014).

5 Yashunsky, D. V., Borodkin, V. S., Ferguson, M. A. & Nikolaev, A. V. The chemical synthesis of bioactive glycosylphosphatidylinositols from *Trypanosoma cruzi* containing an unsaturated fatty acid in the lipid. *Angew Chem Int Ed Engl* **45**, 468-474, doi:10.1002/anie.200502779 (2006).

6 Heng, L., Ning, J. & Kong, F. Synthesis of a mannotetraose—the repeating unit of the cell-wall mannans of *Microsporum gypseum* and related species of *Trychophyton*. *J Carbohydr Chem* **20**, 285-296, doi:10.1081/CAR-100104864 (2001).

7 Ma, Z., Zhang, J. & Kong, F. Synthesis of two oligosaccharides, the GPI anchor glycans from *S. cerevesiae* and *A. fumigatus*. *Carbohydr Res* **339**, 29-35, doi:10.1016/j.carres.2003.09.030 (2004).

8 Zhu, Y. & Kong, F. Highly efficient synthesis of the mannose nonasaccharide of the N-glycan expressed on the HIV glycoprotein gp120. *Synlett* **2001**, 1217-1220, doi:10.1055/s-2001-16062 (2001).

9 Osborn, M. J. *et al.* High-affinity IgG antibodies develop naturally in Ig-knockout rats carrying germline human IgH/Igκ/Igλ loci bearing the rat CH region. *J Immunol* **190**, 1481-1490, doi:10.4049/jimmunol.1203041 (2013).

10 Ma, B. *et al.* Human antibody expression in transgenic rats: Comparison of chimeric IgH loci with human V_H, D and J_H but bearing different rat C-gene regions. *J Immunol Methods* **400–401**, 78-86 (2013).

11 Popov, A. V., Zou, X., Xian, J., Nicholson, I. C. & Brüggemann, M. A human immunoglobulin λ locus is similarly well expressed in mice and humans. *J Exp Med* **189**, 1611-1620, doi:10.1084/jem.189.10.1611 (1999).

12 Korber, B., Foley, B., Kuiken, C., Pillai, S. & Sodroski, J. in *Human Retroviruses and AIDS 1998: A compilation and analysis of nucleic acid and amino acid sequences* (eds B. Korber *et al.*) Ch. PART III. Analyses, 102-111 (Theoretical Biology and Biophysics Group, Los Alamos National Laboratory, 1998).
